# Genome-Wide Identification of RNA Silencing-Related Genes and Their Expressional Analysis in Response to Heat Stress in Barley (*Hordeum vulgare* L.)

**DOI:** 10.3390/biom10060929

**Published:** 2020-06-18

**Authors:** Éva Hamar, Henrik Mihály Szaker, András Kis, Ágnes Dalmadi, Fabio Miloro, György Szittya, János Taller, Péter Gyula, Tibor Csorba, Zoltán Havelda

**Affiliations:** 1 Department of Plant Biotechnology, Agricultural Biotechnology Institute, National Agricultural Research and Innovation Centre, 2100 Gödöllő, Hungary; hamar.eva@abc.naik.hu (É.H.); szaker.henrik.mihaly@abc.naik.hu (H.M.S.); kis.andras@abc.naik.hu (A.K.); dalmadi.agnes@abc.naik.hu (Á.D.); fabio.miloro@abc.naik.hu (F.M.); szittya.gyorgy@abc.naik.hu (G.S.); 2 Department of Plant Sciences and Biotechnology, Georgikon Faculty, University of Pannonia, 8360 Keszthely, Hungary; taller@georgikon.hu; 3 Doctoral School of Biology, Faculty of Natural Sciences, Eötvös Loránd University, 1053 Budapest, Hungary

**Keywords:** barley, RNA silencing, Dicer-like (DCL), Argonaute (AGO), RNA-dependent RNA polymerase (RDR), heat stress

## Abstract

Barley (*Hordeum vulgare* L.) is an economically important crop cultivated in temperate climates all over the world. Adverse environmental factors negatively affect its survival and productivity. RNA silencing is a conserved pathway involved in the regulation of growth, development and stress responses. The key components of RNA silencing are the Dicer-like proteins (DCLs), Argonautes (AGOs) and RNA-dependent RNA polymerases (RDRs). Despite its economic importance, there is no available comprehensive report on barley RNA silencing machinery and its regulation. In this study, we in silico identified five DCL (*HvDCL*), eleven AGO (*HvAGO*) and seven RDR (*HvRDR*) genes in the barley genome. Genomic localization, phylogenetic analysis, domain organization and functional/catalytic motif identification were also performed. To understand the regulation of RNA silencing, we experimentally analysed the transcriptional changes in response to moderate, persistent or gradient heat stress treatments: transcriptional accumulation of siRNA- but not miRNA-based silencing factor was consistently detected. These results suggest that RNA silencing is dynamically regulated and may be involved in the coordination of development and environmental adaptation in barley. In summary, our work provides information about barley RNA silencing components and will be a ground for the selection of candidate factors and in-depth functional/mechanistic analyses.

## 1. Introduction

Barley (*Hordeum vulgare* L.) is the fourth most important crop in the world in terms of cultivated area (50 MHa) and grain yield produced (140 Mt) [1]. Barley grain has a high nutritional component content [2] and is used as a human food and animal feed. Grain germination speed and consistency of endosperm cell wall breakdown into fermentable sugars makes it a primal raw material for the production of alcoholic beverages or biofuels [1,3,4]. Barley straw contains a high level of lignocellulose, that may also be used as a form of renewable energy [4]. Beyond the obvious economic importance, studying barley is useful for understanding and increasing crop resilience. Barley domestication over ten thousand years ago offers strong evidence of its capacity to acclimate to a wide range of environments; indeed, barley was proposed as a good model for adaptation studies [5]. Although relatively tolerant to abiotic stresses among cereal crops, heat stress during reproductive phase negatively affects barley grain yield and quality [6,7]. Barley genetic variation may be employed for the development of efficient strategies to enhance its productivity under diverse climatic conditions. There are over 400,000 barley accessions available [8]. Germplasm availability and expansion may be critical for the sustained high yield of crops under climate change and global warming conditions.

RNA silencing is an evolutionary conserved sequence-specific gene-inactivation pathway. Originally evolved as an immunity system [9,10], RNA silencing acquired several cellular roles including developmental regulation, stress responses, or chromatin organisation. RNA silencing may act both at transcriptional (Transcriptional Gene Silencing, TGS) or post-transcriptional (Post-Transcriptional Gene Silencing, PTGS) levels [11,12]. First discovered in plants, it was later described in many other eukaryotic organisms [13,14,15].

Mechanistically, RNA silencing pathway can be divided into distinct phases, such as the initiation phase, effector phase, and under specific circumstances, amplification phase. The trademark molecules of RNA silencing are the small RNAs (sRNAs) [16,17]. These are 21–24 nucleotide (nt)-long double-stranded, 2-nt 3′-overhang RNA molecules generated by the RNase III-type enzymes, called DICERs, in plants DICER-like proteins (DCLs) [18,19,20]. The most completely described plant dicot model *Arabidopsis thaliana* genome encodes four DCLs (*At*DCL1–4), each having specialised functions. *At*DCL1 generates micro RNAs (miRNAs) that function primarily in development and environmental adaptation, *At*DCL2 and *At*DCL4 are required for vegetative phase change and disease resistance through phased small interfering RNAs (pha-siRNAs) and/or trans-acting siRNAs (ta-siRNAs) generation, while *At*DCL3 produces 24-nt heterochromatic siRNAs (hc-siRNAs) that directs RNA-directed DNA methylation (RdDM)-mediated epigenetic regulation [17,21,22]. *At*DCL2, *At*DCL3 and *At*DCL4 have partially redundant function in antiviral defence and genome maintenance [23,24]. Monocots evolved a further DCL, the DCL5 (formerly named DCL3b) via sub-functionalization of DCL3 (in monocots formerly DCL3a). The main function of DCL5 is the production of monocot-specific 24-nt pha-siRNAs during generative organ development [25,26,27,28,29,30]. DCL endonucleases contain several characteristic domains, including DEAD, Helicase-C, Dicer dimer (DUF283), PAZ, RNase III (A and B, essential for endonuclease activity) and double-stranded RNA binding motif (DSRMa and DSRMb) domains [28,31]. DCL substrates are either long double-stranded RNA (dsRNA) precursors or single-stranded RNAs containing hairpin structures. Following their biogenesis, sRNAs associate with AGO proteins, RNase H-type endonucleases [32,33].

AGOs, alongside the incorporated sRNA (and other associated proteins), form the RNA-induced Silencing Complex (RISC). AGOs are the executor components, while sequence-specificity of RISC activity is provided by the sRNA. Domain structures of the previously characterised AGOs revealed the conservation of four units: N-terminal, PAZ, MID and PIWI domains. N-terminal domain plays a critical role in target cleavage and dissociation of the cleaved RNA strand, PAZ is essential for sRNA’s 2-nt 3′ overhang binding, MID anchors sRNA’s 5′ phosphate, while PIWI of certain AGOs has RNase activity [33,34]. The four metal-coordinating catalytic residues aspartic acid, glutamic acid, aspartic acid, aspartic acid/histidine (D760, E788, D845 and D/H986, DED[D/H]) tetrad and a further conserved histidine residue (H798 in *At*AGO1) within the PIWI domain are crucial for endonuclease/effector capacity of AGOs in vitro and in vivo [35,36,37]. The presence of the catalytic core, in some specific cases, is not sufficient for target cleavage ability [38,39]. RISCs find their target RNAs via sequence complementarity, then cleave or repress their translation during PTGS, or block their transcription due to DNA methylation and heterochromatin formation during TGS [9,40].

Aberrant cellular RNAs lacking a cap structure or poly-A tail trigger RNA silencing through the activity of RNA-dependent RNA polymerases (RDRs) [41,42,43], the third main class of RNA silencing *trans* factors. RDR enzymes recognise aberrant RNAs (or maybe tethered by AGOs bound to the cleaved substrates) and convert them into dsRNAs, providing new substrates for DCLs. RDRs, therefore, can both initiate and amplify the silencing of genes/transcripts homologous to the trigger itself [44,45,46]. Domain organisation of RDRs is relatively simple, as they consistently possess an RNA-dependent RNA polymerase (RdRP) domain [41,43]. There are three major groups of eukaryotic RDRs: RDRα, RDRβ and RDRγ. In the plant lineage, RDRβ group has been lost [41,43]. In *A. thaliana*, RDRα has three (*At*RDR1, 2 and 6), and RDRγ also three representatives (*At*RDR3, 4 and 5, due to high similarity these were renamed as RDR3a, 3b and 3c, respectively). RDRα proteins all share the conserved C-terminal canonical DLDGD catalytic core, while RDRγ proteins possess an atypical DFDGD motif [41,43]. Additionally, RDRα proteins have an N-terminal RNA-recognition motif (RRM). RDRα enzymes were shown to be involved in endogenous gene regulation by the ta-siRNA and pha-siRNA pathways [22,29], antiviral silencing [24,47,48,49], heterochromatin organisation, and genome defence [17,21] while the roles of RDRγ are presently unknown.

Environmental factors, such as extreme hot temperature, cause severe damage to the plant cells and organism by reducing fitness, endangering survival and propagation. Heat stress during cereal grain filling period causes a decrease in the synthesis of storage proteins and starch and leads to grain abortion [50]. There are a number of signal transduction pathways that initiate cellular responses in order to repair or prevent further damage [51,52]. sRNAs have been proposed to actively take part in fine-tuning the balance between development and stress responses during stress and in the post-stress period [53,54,55,56,57].

Accumulation of certain sRNAs and expression of RNA silencing factors is dynamically modulated during development and stress, suggesting that RNA silencing regulation is required for efficient response to environmental cues. Specific barley miRNAs are transcriptionally and post-transcriptionally regulated by heat [58]. *A. thaliana At*AGO proteins have varying levels and patterning during different developmental stages and/or organs [59]. *Brachypodium distachyon BdAGOs* and *BdDCLs* are expressed in an organ- and developmental stage-specific manner [27,60,61]. Similarly, microarray-based profiling of rice (*Oryza sativa*) *OsDCLs*, *OsAGOs* and *OsRDRs* during vegetative and reproductive stage or in response to abiotic stresses revealed their specific and flexible regulation [25]. Maize (*Zea mays*) *Zm*AGO proteins are differentially produced under a variety of abiotic stresses [62]. These findings strongly suggest a perpetual and effective regulation of RNA silencing machinery at the transcriptional level.

*DCL*, *AGO*, and *RDR* gene families have several identified members in different crop species e.g., rice, maize, tomato, cucumber, grapevine, foxtail millet, and pepper [26,63,64,65,66,67]. Although barley is among the first cultivated plants with great economic importance and with a fully sequenced genome, strikingly, there is still only scattered information available on its RNA silencing machinery and its transcriptional regulation. In the present work, we identified the members of barley *DCL (HvDCL)*, *AGO (HvAGO)*, and *RDR (HvRDR)* gene families, analysed their phylogenetic relationship to model and crop plants, investigated their domain architecture and core catalytic motifs/regions. Transcriptional changes of RNA silencing *trans* factors suggests that siRNA-based silencing may play a role in adaptation to heat stress conditions.

## 2. Materials and Methods

### 2.1. Identification of HvDCL, HvAGO and HvRDR Homologues

Amino acid (aa) sequences of *A. thaliana* DCL, AGO, and RDR proteins (*At*DCLs, *At*AGOs, *At*RDRs) were obtained from the UniProt database (www.uniprot.org) [68]. Hidden Markov-model (HMM) profiles were generated with HMMer tool (https://www.ebi.ac.uk/Tools/hmmer) [69]. A profile-based search was performed with default parameters against the Ensembl Genomes Plants database (http://ensemblgenomes.org/node/114254) [70]. Significant matches for *H. vulgare* were listed and filtered manually to keep only those sequences which are greater than 50 kDa and showed complex domain organisation, typical to respective protein families according to Pfam (https://pfam.xfam.org/) [71]. Nucleotide and amino acid sequences of *Hv*DCL, *Hv*AGO, and *Hv*RDR candidates were downloaded from Ensembl Plants database (https://plants.ensembl.org/) [71]. Predicted isoelectric points of respective amino acid sequences were calculated with ExPasy (https://web.expasy.org/compute_pi/) [72].

### 2.2. Phylogenetic Analysis, Chromosomal Localisation and Identification of Conserved Motifs

To compare the predicted *Hv*DCL, *Hv*AGO and *Hv*RDR homologues with other monocotyledonous models and the dicotyledonous *A. thaliana*, amino acid sequences of *Z. mays* and *O. sativa* DCL, AGO, and RDR (*Zm*- and *Os*AGO, -DCL and -RDR) proteins were downloaded from UniProt database (www.uniprot.org) [68]. MEGA X software v10.1.8 [73] was employed to perform multiple alignments using the Clustal W algorithm [74] and generate neighbour-joining [75] phylogenetic trees with 1000 bootstrap replicates [76]. Barley’s predicted RNA silencing components were named according to their phylogenetic relationship with the previously identified members of the same protein family. The chromosomal localisation of *HvDCL, HvAGO* and *HvRDR* genes were obtained from Ensembl Plants database [70]. SMART web server (http://smart.embl-heidelberg.de) was used to search for known protein domains. MEME web server (http://meme-suite.org/tools/meme) [77] was employed for conserved motif prediction. Search parameters were the following: optimum motif width 6 ≤ *n* ≤ 200, maximum number of motifs: 20. Those motifs, which did not belong to structural domains of the analysed protein family were rejected. The identified motifs were annotated using Pfam [71]. The percentage of similarity and identity of respective protein sequences were calculated with Ident and Sim online Sequence manipulation tool (http://www.bioinformatics.org/sms2/ident_sim.html) [78].

### 2.3. Plant Material and Abiotic Stress Treatments

Barley (*H. vulgare* L. cv. Golden promise) plants were grown in growth chambers at 18 °C, 16 h daylength. For expression analysis of RNA silencing-related genes, roots and leaves of two-weeks-old plants were collected and frozen in liquid nitrogen. For the heat stress (HS) treatments, 16-day-old barley plants were subjected to persistent heat (40 °C for 24 h), or to mimic natural hot temperature conditions, gradient heat stress (gHS) was employed, i.e., temperature was elevated from 21 °C to 37 °C in a course of 4 h as described before [55]. Leaf samples were collected from control and treated plants immediately following the treatments, frozen in liquid nitrogen and stored at −70°C until use.

### 2.4. RNA Isolation, RT-PCR, Semiquantitative RT-PCR and RT-qPCR Analysis

Frozen leaf samples were homogenised in sterile mortars. RNA was isolated with Trizolate reagent (UD GenoMed Ltd., Debrecen, Hungary) according to the manufacturer’s instructions. The RNA concentration and 260/280 ratio were determined with NanoDrop spectrophotometer (Thermo Fisher Scientific Inc. Waltham, MA, USA).

First-strand cDNA was synthesised from 4 μg of DNase I (Thermo Fisher Scientific Inc., Waltham, MA, USA ) treated total RNA using RevertAid kit (Thermo Fisher Scientific Inc., Waltham, MA, USA) according to the manufacturer’s protocol. Every putative *HvDCL*, *HvAGO* and *HvRDR* genes were subjected to RT-PCR and semiquantitative PCR with manually designed gene-specific primers (Table S1). *HvACTIN* (GeneBank ID: AY145451) was used as an internal control. For the PCRs, Phire II hot-start polymerase (Thermo Fisher Scientific Inc., Waltham, MA, USA) was used following the manufacturer’s instructions. The reaction profile was the following: initial denaturation at 98 °C for 3 min, then 35 cycles of three steps consisting of denaturation at 98 °C for 5 sec, annealing of primers at a primer specific annealing temperature for 30 sec, elongation at 72 °C for 8–20 sec, and final elongation at 72 °C for 5 min.

For semiquantitative RT-PCR, the number of cycles was adjusted to 30.5 μL of the reactions were separated on 1.2% agarose gel (Lonza Inc. Rockland, ME, USA) and analysed with a ChemiDoc gel imaging system (Bio-Rad, Hercules, CA, USA) using ImageLab software (Bio-Rad, Hercules, CA, USA).

For RT-qPCR assays, 1 μg of DNase-treated total RNA and random primer was used for the first-strand complementary DNA reaction according to the manufacturer’s instructions (New England Biolabs, Ipswich, MA, USA). RT-qPCRs were done using qPCR Master Mix (NEB, M3003S, www.neb.com) according to the manufacturer’s instructions. RT-qPCR reactions were run in a LightCycler^®^ 96 Real-Time PCR machine (Roche, Basel, Switzerland). The reaction profile was the following: preincubation: 1 min 95 °C; amplification: 45 cycles of 15 sec 95 °C and 30 sec 60 °C; melting: 10 sec 95 °C, 60 sec 65 °C and continuous heating to 97 °C; cooling: 30 sec 37 °C. At least three biological and three technical replicates were assessed in each experiment and standard error bars are shown. *p*-values were calculated using unpaired two-tailed Student t-test to assess the significance of differences between the means of the treated and untreated samples. For a list of primers please see Table S2.

### 2.5. RNA-Seq Analysis

Sequence data associated with the study by Pacak et al., [79] were downloaded from the NCBI Gene Expression Omnibus database (accession number: GSE82134). The sequences were aligned to the *H. vulgare* IBSC v2 transcriptome and the normalised expression values (transcript per million, TPM) were calculated with kallisto v0.44.0, normalised between samples with sleuth v0.29.0 [80] and represented on heat maps that were prepared with the R package pheatmap [81]. Differential expression analysis was performed with sleuth v0.29.0 [80]. To test whether there is a significant difference between the means of the heat-stressed (HS) and the not-treated (NT) samples, a Wald-test was applied. The calculated *p*-values were corrected for multiple testing using the Benjamini-Hochberg method [82]. A gene was considered significantly differentially expressed if the *Q*-value was lower than 0.05 (5% false discovery rate).

## 3. Results

### 3.1. Identification and Structural Analysis of DCL, AGO and RDR Genes in Barley

**Figure 1:**
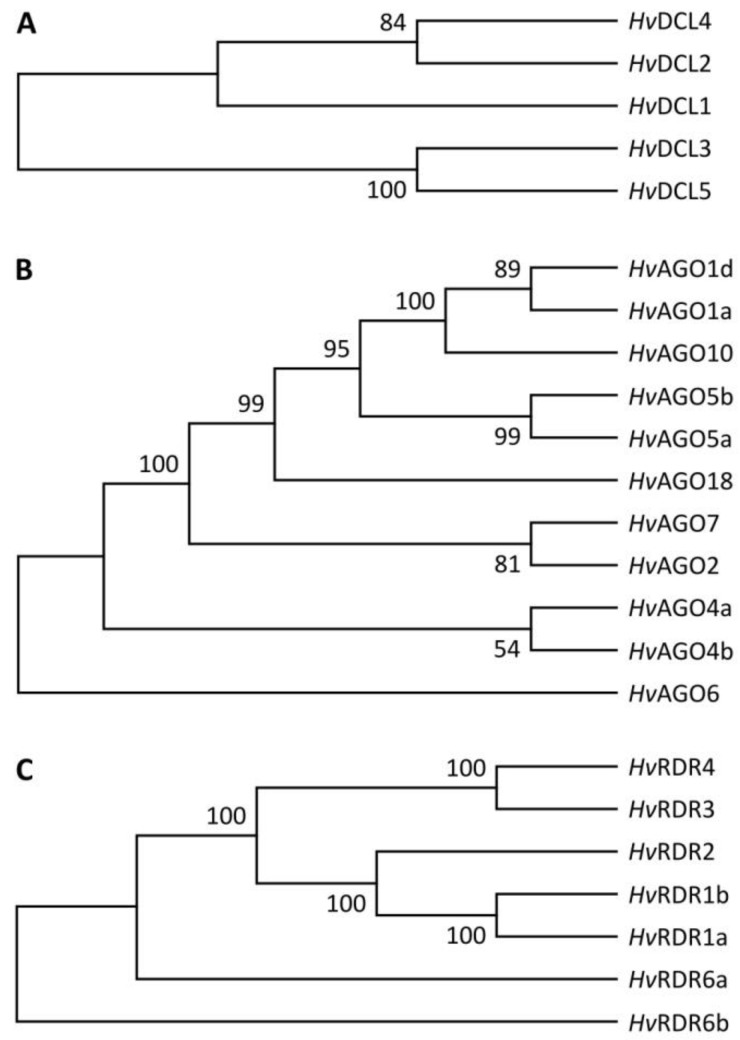
Phylogenetic relationship of the predicted barley DCL (**A**), AGO (**B**), and RDR (**C**) proteins. The evolutionary history was inferred using the Neighbor-Joining method [75]. The bootstrap consensus tree inferred from 1000 replicates [76] is taken to represent the evolutionary history of the taxa analysed. Branches corresponding to partitions reproduced in less than 50% bootstrap replicates are collapsed. The percentage of replicate trees in which the associated taxa clustered together in the bootstrap test (1000 replicates) are shown next to the branches. The evolutionary distances were computed using the Poisson correction method and are in the units of the number of amino acid substitutions per site. All ambiguous positions were removed for each sequence pair (pairwise deletion option). There were a total of 2145 (**A**), 1302 (**B**), and 1393 (**C**) positions in the final dataset. Evolutionary analyses were conducted in MEGA X [73].

To identify barley’s RNA silencing associated genes, the *A. thaliana* DCL, AGO and RDR protein’s amino acid sequences were used as queries to build a Hidden Markov-model (HMM). According to HMM profile analysis, a total number of five *HvDCL*s, eleven *HvAGO*s and seven *HvRDR* genes were predicted in the barley genome. *H. vulgare* silencing components were named based on the closest relative in *A. thaliana*, or monocot species *O. sativa* and *Z. mays* (Figure 1).

The putative RNA silencing gene chromosomal location, structure and encoded protein predicted features are listed in Table 1. Exon number of *HvDCLs* was approximately the same as their *A. thaliana* homologues (Table 1 and Table S3). Genes encoding DCL homologues in barley possess ORFs ranging from 4428 to 5994 bp. *HvAGO* ORF lengths are shorter, ranging from 2451 to 3654 bp. *HvAGO2* and *HvAGO7* possess only three exons while the other *HvAGO*s have a significantly higher number of exons (21–23); this is similar to *A. thaliana* orthologues (Table 1 and Table S3). The exon number of *HvRDR* genes showed great variety between paralogues (e.g., *HvRDR6a* has two exons, *HvRDR3* and *4* has 19), but are similar in the corresponding orthologues (Table 1 and Table S3). *HvRDRs* have rather uniform ORF lengths, ranged between 3348 and 3684 bp. Isoelectric point and molecular weight of putative DCL, AGO, and RDR proteins were also estimated (Table 1).

To explore the evolution of main RNA silencing gene families in barley, we analysed the genomic distribution by localising the genes on chromosomes. The exact chromosomal sites of *HvDCL*, *HvAGO* and *HvRDR* genes were obtained from Ensembl Plants barley (IBSC_v2) database (Table 1). The RNA silencing associated genes are distributed unevenly on the seven haploid chromosomes of barley, which is similar to RNA silencing genes in multiple monocot genomes [25,26].

The five *HvDCL* homologues are distributed on five chromosomes. In monocots, *DCL3* gene has been duplicated: *DCL3* paralogous gene duplication was suggested to occur before the common ancestor of barley and rice (ca. 60 mya) since *DCL3* and *DCL5* orthologues could be detected in rice, maize, and *Aegilops tauschii*, a progenitor of wheat [28]. *HvAGO* homologues are present on chromosomes 2, 3, 4, 6, 7; Besides this, *HvAGO6* and *HvAGO7* are located on the uncharacterised chromosome, their exact location is still uncertain, according to the latest barley genome assembly. To note, *HvAGO1d* is localised in close proximity of chromosome 7 telomere region. *HvRDR* homologous genes of barley were detected on chromosomes 2, 3, and 6, respectively. Chromosome 3 hosts four putative barley *RDR*s (*HvRDR3, HvRDR4, HvRDR6a*, and *HvRDR6b*). Interestingly, *HvRDR1a* and *HvRDR1b* are located in close proximity to each other, only three predicted genes separate them on chromosome 6. As they share about 81% sequence identity, they can be the result of gene duplication (Supplementary Material 1). It was suggested previously that duplication of *HvRDR1* and *HvRDR6* possibly reflects the divergence in disease resistance between monocots and dicots [83].

### 3.2. Domain Analysis and Phylogenetic Relationship of Identified Barley DCL, AGO and RDR Proteins

**Figure 2:**
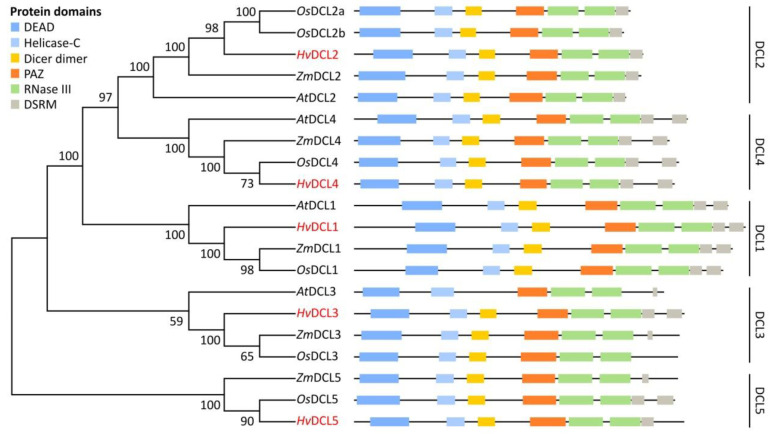
Phylogenetic relationship and conserved domains of *A. thaliana*, *O. sativa, Z mays* and *H. vulgare* DCL proteins. Unrooted NJ trees were constructed with MEGA X software as described in Figure 1 legend. The bootstrap values are shown next to the branches. Conserved domains were identified with the SMART server [84]. The protein domain abbreviations are the followings: DEAD (DEAD-like helicases superfamily), Helicase-C (helicase superfamily C-terminal domain), Dicer dimer (Dicer dimerisation domain, or DUF283), PAZ (Piwi, Argonaute, Zwille), RNAse III (Ribonuclease III family), DSRM (Double-stranded RNA binding motif). The barley DCLs identified in this study are marked with red.

To investigate their potential functionality, we examined the presence and order of characteristic domains within the barley silencing proteins. For this, we performed domain search with the SMART tool [84]. All important regions of DCL proteins, including DEAD, Helicase-C, Dicer dimer (DUF283), PAZ, RNase III and dsRNA-binding motif (DSRM) were found in all *Hv*DCLs. The domain order was consistent with DCLs of *A. thaliana* or other monocot species (Figure 2). Domain search with MEME tool using the amino acid sequences of *Hv*DCL candidates predicted the same domains and unravelled small variations between putative paralogs/orthologs, however, the domains were only partially recovered (Tables S4 and S5).

**Figure 3:**
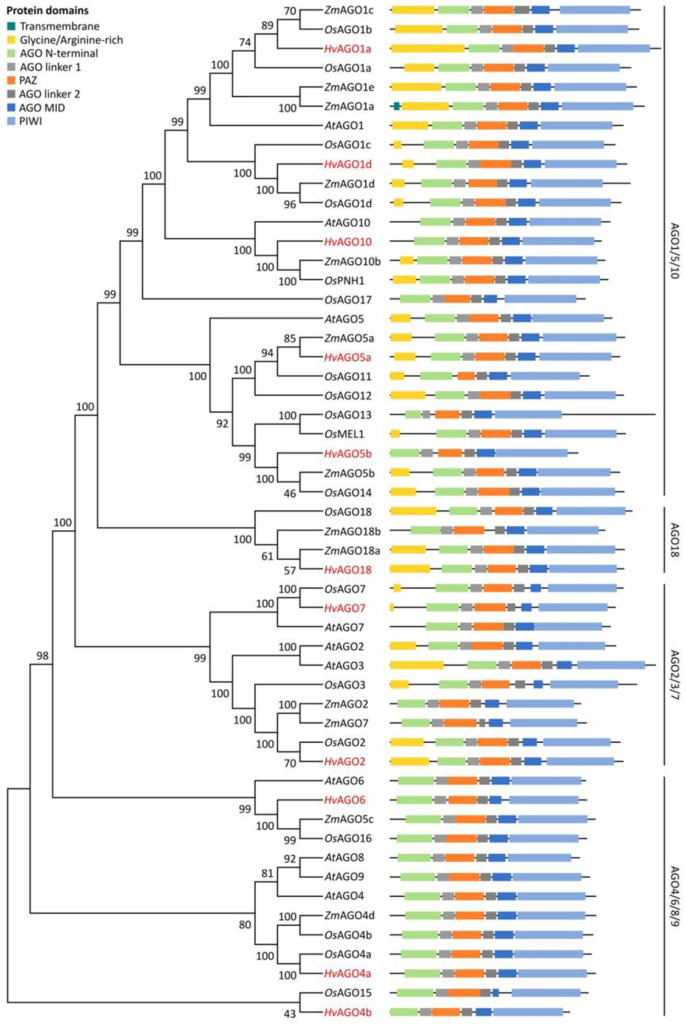
Phylogenetic relationship and conserved domains of *A. thaliana*, *O. sativa*, *Z mays* and *H. vulgare* AGO proteins. Phylogenetic trees were constructed as described in Figure 1 legend. The bootstrap values are shown next to the branches. Conserved domains were identified with the SMART server [84]. The barley AGOs identified in this study are marked with red.

Similarly, all predicted *Hv*AGO proteins contained the characteristic domains in the precise order, namely N-terminal, Argonaute linker 1 (L1), PAZ, L2, MID and PIWI (Figure 3, Table S4), as the previously characterised AGO proteins [33,37,85]. Besides these, we mined for glycine and arginine (GR)-rich domains upstream to the AGO N-domains, based on information from *A. thaliana* and *Chlamydomonas reinhardtii* systems [86]. *Hv*AGO1a, 1d, 5a, 18, 2 and 7 were found to contain GR-rich regions consistent with previous data [86]. GR-rich regions may facilitate interaction with ribosomes to enable AGO tethering for the effector phase of silencing [86]. The observation that species including *O. sativa, Z. mays, T. aestivum* or *B. distachyon* also encode at least one AGO protein having GR-rich region [86], suggests that targeting of specific AGOs to translating mRNA targets is a widespread phenomenon in monocots. MEME search revealed complementary results with slight differences within certain AGO homologs (Tables S4 and S6).

**Figure 4:**
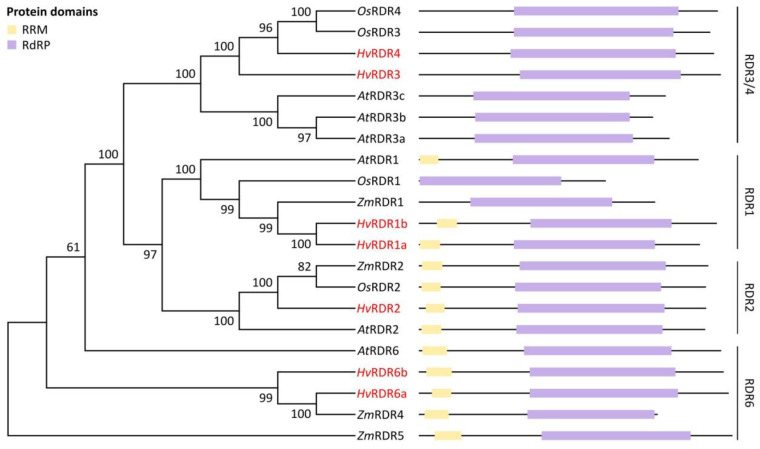
Phylogenetic relationship and conserved domains of *A. thaliana*, *O. sativa*, *Z mays* and *H. vulgare* RDR proteins. Unrooted NJ trees were constructed with MEGA X software as described in Figure 1 legend. The bootstrap values are shown next to the branches. Conserved domains were identified with the SMART server [84]. The protein domain abbreviations are the followings: RRM (RNA-recognition motif), RdRP (RNA-dependent RNA polymerase domain). The barley RDRs identified in this study are marked with red.

RDR proteins are defined by the presence of the RdRP domain. Additionally, RDRα class proteins contain an RNA-recognition motif (RRM). We have found the RdRP domain in all predicted *Hv*RDRs and the RRM domain in RDR1a, 1b, 2, 6a and 6b proteins by SMART search (Figure 4). The *de novo* MEME prediction recovered the RdRP but not the RRM motifs (Tables S4 and S7).

To investigate evolutionary relationship between the dicotyledonous model *A. thaliana* and the monocotyledonous crops rice, maize and barley RNA silencing proteins, unrooted NJ phylogenetic trees were constructed for each protein family from multiple alignments of full-length amino acid sequences (Figure 2, Figure 3 and Figure 4). Full alignment of all *A. thaliana* and barley DCL, AGO and RDR proteins can be found in supplementary materials (Figures S1–S3, respectively).

According to the phylogenetic analysis, the examined DCL sequences were grouped into clades with well-supported bootstrap values (Figure 1 and Figure 2). Besides the four clades described in dicots (DCL1–4 clades), monocots evolved a functionally different DCL, the DCL5 [22,30]. This sub-branch of DCL3 family was therefore assigned in a fifth clade (DCL5 clade). The five candidate *Hv*DCLs were divided into these clades and named as *Hv*DCL1, *Hv*DCL2, *Hv*DCL3, *Hv*DCL4, and *Hv*DCL5 based on their high level of sequence similarities with the other members of the same taxonomic group (Figure 2, Supplementary Material 1).

The unrooted NJ tree generated from aligned full-length *A. thaliana*, rice, maize and barley AGO protein sequences were separated into four clades, namely AGO1/5/10, AGO18, AGO2/3/7 and AGO4/6/8/9 (Figure 3), from which three correspond to the *A. thaliana* AGO funding clades [87]. Five *Hv*AGOs, namely *Hv*AGO1a, *Hv*AGO1d, *Hv*AGO10, *Hv*AGO5a and *Hv*AGO5b were clustered into AGO1/5/10 clade with respective members of *A. thaliana*, rice and maize AGOs. Additionally, a further sub-branch of this group evolved in grasses, the AGO18 clade [87]. Accordingly, *Hv*AGO18 grouped together with *Os*AGO18, *Zm*AGO18a and *Zm*AGO18b, and did not contain any *At*AGO protein. The AGO2/3/7 clade contained two barley proteins, namely *Hv*AGO2 and *Hv*AGO7 which had 50% and 66%, and 82% and 66% sequence similarity with respective *A. thaliana* and rice proteins. Three putative barley proteins were clustered into the AGO4/6/8/9 clade, which were designated *Hv*AGO4a, *Hv*AGO4b and *Hv*AGO6 based on their high level of sequence similarity (75%, 59% and 68%) with respective *A. thaliana* proteins (Figure 3 and Supplementary Material 1).

According to the phylogenetic analysis, RDR proteins of *A. thaliana*, maize, rice and barley were divided into four clades (Figure 4). *At*RDR1, *Zm*RDR1 and *Os*RDR1 were clustered together in the first subfamily with two barley proteins designated *Hv*RDR1a and *Hv*RDR1b based on sequence similarity with *At*RDR1 (RDR1 clade). Next clade contains one barley member, *Hv*RDR2 (RDR2 clade). In the third clade, a total of six *A. thaliana*, maize and rice proteins were clustered together with two barley RDRs.

As sequence identity to each other was 42%, similarity 56%, these were named *Hv*RDR3 and *Hv*RDR4 (RDR3/4 clade). The last clade contained RDRs similar to *At*RDR6. Two barley members of this group were designated *Hv*RDR6a and *Hv*RDR6b, according to their high sequence similarity to *At*RDR6 (63% and 64%) and to each other (70%) (RDR6 clade) (Figure 4, Supplementary Material 1).

### 3.3. Functionally Conserved Amino Acid Residues in Domains of the Identified RNA Silencing Factors

To further corroborate the potential functionality and get an insight into the possible mechanistic activities of the identified barley silencing proteins, we analysed the conservation of regions involved in RNA-binding, enzyme catalysis or other essential features [31,88,89,90].

**Figure 5:**
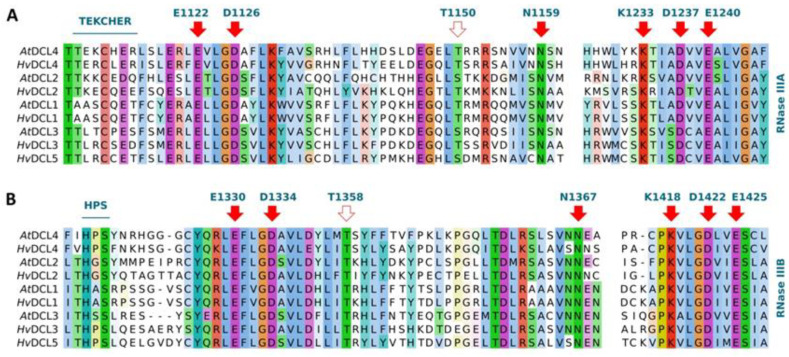
Conservation of functionally critical amino acids between *A. thaliana* and barley DCL proteins. Conserved residues involved in enzyme catalysis within (**A**) Rnase IIIA (E1122, D1126, N1159, K1233, D1237, E1240, red arrow), (**B**) RNase IIIB (E1330, D1334, N1367, K1418, D1422, E1425, red arrow), and (**A**,**B**) RNA-binding motifs (TEKCHER, HPS loops and T1150, T1358 residues, blue line and open arrows). Residue numbers correspond to AtDCL4 amino acid positions. For full-length alignment of DCLs see Figure S1.

In-depth structural data on plant DCLs are very limited and comprises only domains outside the processing centre [31,89]. The catalytic core of *At*DCL4, however, was computationally modelled, providing information about the amino acids involved in dsRNA recognition, binding, or cleavage [90]. Based on these data, we thoroughly characterised barley DCLs (Figure 5 and Figure S4). Both the surrounding region and the catalytic residues directly involved in dsRNA cleavage of RNase IIIA (E1122, D1126, N1159, K1233, D1237 and E1240) and IIIB (E1330, D1334, N1367, K1418, D1422 and E1425) were found to be well conserved between all *A. thaliana* and barley DCLs (Figure 5A,B) [88,90]. Connector helix core L/IPSI/L/MM(X)_11_LK/R was also conserved, except in *Hv*DCL3 (Figure S4). The 3′ RNA binding pocket of PAZ and 5′ RNA binding pocket have high variability among the studied structures; accordingly, we could not locate these unambiguously. N-terminal part of PAZ-loop (NLL motif) responsible for dsRNA binding, was conserved, except in *Hv*DCL2 (Figure S4). The *At*DCL4 RNase IIIA TEKCHER motif and RNase IIIB analogous HPSYN loop were predicted to interact with dsRNA; only limited conservation of these could be observed (Figure 5A,B). T1150, R1151 (RNase IIIA) and T1358, R1415 (RNase IIIB) residues were predicted to contribute to dsRNA positioning at cleavage sites; at T1150, a conserved threonine or serine was found, implying that a hydroxylic side chain is required at this point (Figure 5A, open arrow); T1358 residue (RNase IIIB) was also conserved (Figure 5B, open arrow). In contrast, residues corresponding to R1151 (RNase IIIA) and R1415 (RNase IIIB) were highly variable, implying that these are not critical for dsRNA positioning. In summary, data suggest that, although binding/positioning of dsRNA is potentially different, the cleavage catalysis driven by *Hv*DCLs mechanistically is very likely similar to the RNase III type enzymes described before [90]. Besides the regions discussed above, we have found other highly conserved residues and stretches (Figure S1), implying their structural and/or functional relevance in a clade-independent manner. The biological relevance of these remains to be validated experimentally.

Next, we took a closer look into the conservation of functional motifs in *Hv*AGOs. PAZ domain recognise and bind the 3′ end of sRNAs [91,92]. As data on plants are not available, we searched for conserved motifs based on *D. melanogaster* and human AGO data [92,93]. Only limited conservation was found in the amino acids involved in the sRNA 3′ end binding between *Dm*AGO1 aromatic cluster α3 (L, Y, F and Y, red squares), human *Hs*AGO-eIF2C1 (H, Y, F, Y and L, blue circles) and *A. thaliana* or barley AGOs (Figure S5). These residues potentially may take part in sRNA 3′-end binding. Besides these, other highly conserved residues/motifs with potential functional importance are present within this area (Figures S2 and S5).

**Figure 6:**
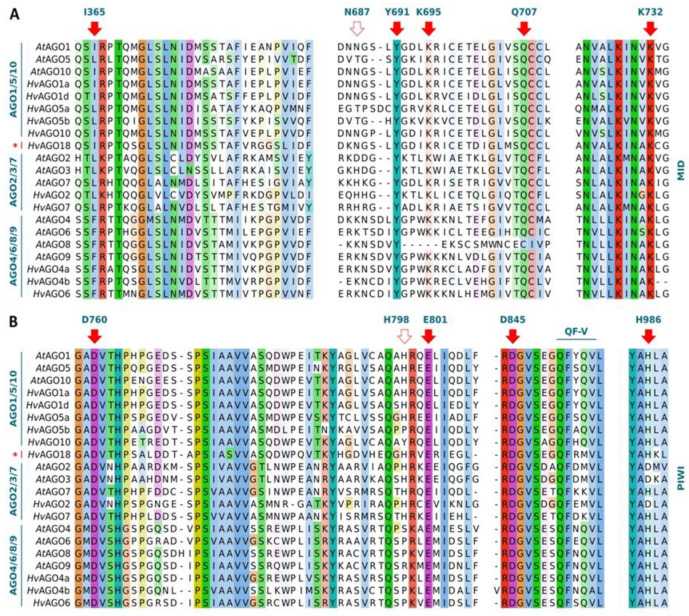
Functionally conserved positions within MID and PIWI domains of *A. thaliana* and barley AGO proteins. Residues within (**A**) MID domain sRNA-target interaction (I365, red arrow), 5′ terminal nucleotide selection (N687, open arrow) and 5′-phosphate-binding (YKQK, red arrows) residues and (**B**) PIWI domain catalytic tetrad ((DED[D/H], red arrows), additional H residue (open arrow) and QF-V motif (blue line)) are indicated. Residue numbers correspond to AtAGO1 amino acid positions. AGO clades are shown on the left. Monocot-specific AGO18 clade is marked with a red star. For full-length alignment of AGOs see Figure S2.

AGO MID domain functions in sRNA binding, sorting and sRNA-target RNA pairing. Residues Y691, K695, Q707 and K732 (numbers correspond to positions in *At*AGO1) actively involved in sRNA 5′-phosphate-binding [94] were fully conserved in all *A. thaliana* and barley AGOs (Figure 6A). sRNA sorting into different AGOs depends on multiple features, including sRNA length and 5′ end nucleotide type [95,96,97]. sRNA 5′ terminal nucleotide is recognised by the nucleotide specificity loop [95,98] within the MID domain (Figure 6A). In *At*AGO1, the key asparagine (N687) residue within this promotes 5′-uridine (5′-U) binding. *Hv*AGO1a, 1d, 10 and AGO18 all retain the N residue, suggesting similar preference. *Os*AGO1a/b/c were shown to predominantly associate with 5′-U sRNAs, supporting this hypothesis [99]. *At*AGO5 prefers 5′-C sRNAs [97] but permits binding of 5′-A, -G and -U sRNAs as well [95]. AGO5 members of the AGO1/5/10 clade carries a conserved threonine (T) within the nucleotide-binding loop (Figure 6A). *At*AGO4 has a 5′-A ended sRNA binding priority. Insertion of a K and an N687K change (e.g., AGO1 MID-AGO4 chimaera) triggered a conformational change, inducing 5′-A preference instead of 5′-U [98]. All AGO4/6/8/9 clade members own a K at this position, indicating that 5′-A sRNAs may be favoured within this clade. Interestingly, the aspartic acid (D) residue within nucleotide specificity loop of *At*AGO2, important for 5′-A selection [95] is not conserved within AGO2/3/7 clade, instead, a histidine (H) replaces it. *At*AGO7 exclusively associate with miR390, however, no preference for particular 5′-end nucleotide could be established [100]. Maintenance of the H within the nucleotide specificity loop within AGO2/3/7 clade of *A. thaliana* and barley (except *At*AGO2) proteins suggests a functional purpose of this residue.

Isoleucine I365 ensures rapid and precise target binding [101]. I365 is present in *At/Hv*AGO1, 10, 18 proteins, while an L (I/L conservative change, hydrophobic and aliphatic) replaces it in *At/Hv*AGO5 and AGO2/3/7 clade proteins (Figure 6A). A partially non-conservative change occurred at this position in AGO4/6/8/9 clade, with phenylalanine (F, hydrophobic and aromatic). Interestingly, F was found to be preferentially involved in protein-DNA interactions and far less in protein-RNA interactions [102], consistent with chromatin regulatory roles of AGO4/6/8/9 clade proteins [103].

We also investigated if *Hv*AGOs retained the DED[D/H] (DEDH or DEDD) tetrad and the additional histidine (*At*AGO1-H798 analogue) residue within PIWI, directly involved in enzyme catalysis [35,36,37] (Figure 6B). The DED[D/H] tetrad was fully conserved in all *Hv*AGO proteins. Five out of eleven *Hv*AGOs (*Hv*AGO1a, 1d, 5a, 7, and 18) also possessed the additional histidine analogous to *At*AGO1-H798. *Hv*AGO2 contained the DEDD tetrad and the additional H, signature typical to AGO2/3 proteins [104]. However, *Hv*AGO4a, 4b, 5b, 6, and 10 had amino acid substitutions in the *At*AGO1-H798 analogue position. The highly conserved glutamine-phenylalanine-valine (QF-V) motif essential in sRNA duplex recognition and sorting [105] was fully conserved in all *Hv*AGOs (Figure 6B). The clade-specific surroundings of D760, H798, E801 residues and QF-V motif hints to roles in the functional diversification of AGO clades. We also analysed the AGO-hook motifs involved in GW protein partner and sRNA 5′-end binding [106,107,108]: several essential residues of *Hs*AGO2 and hydrophobic stretches within pockets 1 and 2 show conservation between *A. thaliana* and barley AGOs (Figure S6) pointing to biological relevance. Interestingly, some of these regions possess clade-specific amino acid swaps. Residues directly involved in GW protein AGO hooking and sRNA 5′-end binding need to be defined experimentally.

**Figure 7:**
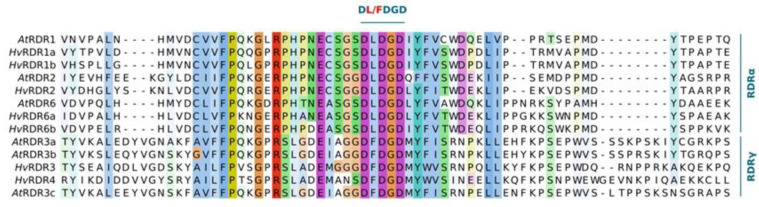
Presence of functionally critical amino acid residues in barley RDR proteins. Catalytic domain within RdRP is shown. For full-length alignment of RDRs see Figure S3.

Finally, we checked the presence of the catalytic core and its surrounding region within barley RDRs. *Hv*RDR1a, 1b, 2, 6a and 6b, belonging to RDRα clade all possessed the canonical DLDGD, while RDRγ clade proteins *Hv*RDR3 and 4 contained the atypical DFDGD catalytic core (Figure 7). The enclosing of the catalytic centre also carried differences between RDRα and RDRγ groups: the PHX_2_EC/AS upstream stretch or the WD dipeptide downstream to the catalytic core is RDRα-specific. These observations further underpin the motif and phylogenetic analysis on *Hv*RDRs (Figure 4). Retention of the active core within all RDRα members suggests that these factors are potentially required at some stage during barley lifecycle.

The high similarity between barley and corresponding genes/proteins of other species, the presence of characteristic domains in the rigorous order (Figure 2, Figure 3 and Figure 4) and functionally important residues/motifs (Figure 5, Figure 6 and Figure 7) suggest that the predicted barley genes/proteins are potentially the orthologues of the previously characterised DCLs, AGOs and RDRs of *A. thaliana* or rice and that these genes are probably functional and required during development and environmental adaptation.

### 3.4. Autoregulation of RNA Silencing

**Figure 8:**
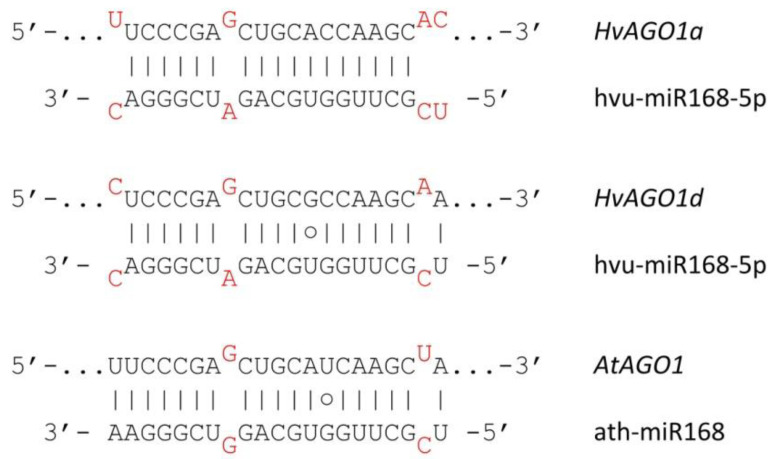
Secondary structure of miR168/target site duplex region of identified barley and *Arabidopsis thaliana* AGO1 transcripts. G/U base-pairs are denoted by a circle, while mismatched bases are marked with red.

RNA silencing factors including *AGO* and *DCL* transcripts were shown to be autoregulated in many plant species by a negative feedback loop involving sRNAs [109,110]. Ath-miR168 and the secondary siRNAs arising from miR168-guided cleavage negatively regulate *AtAGO1* in a complex but also robust manner [39]. This autoregulatory loop seems to be widely conserved among vascular plants, and potentially also occurs in monocots. MiR168 was found in rice, wheat, maize, *B. distachyon* and barley as well [111,112,113,114]. For these reasons, we analysed the potential of *AGO1* being negatively regulated through miR168 in barley. MiR168 target region was found in both *HvAGO1a* and *HvAGO1d* (Figure 8). *Hv*AGO1a and *Hv*AGO1d target-miR168 guide pairing topology and locations of mismatches are very similar to the *A. thaliana* system, suggesting that both *HvAGO1a* and *1b* are probably subjects of an autoregulatory loop. This hypothesis is also supported by a degradome data of barley *HvAGO1a* miR168-mediated cleavage [115].

MiR403 is the negative regulator of *AGO2* and *AGO3*. MiR403 is present only in specific dicot groups, having very similar sequence and few isoforms suggesting a recent evolutionary origin. MiR403 is probably absent in monocots [113,116]. In accordance with this, we were unable to find any potential target region of miR403 in *HvAGO2*. Ath-miR162 has been shown to negatively regulate *AtDCL1* in *A. thaliana* [117]. Although found in rice, miR162 was not detected in barley, wheat or *B. distachyon* [112]. In line with this, we could not find or predict miR162 target region within *Hv*DCL1 gene.

### 3.5. Heat Stress Significantly Alters the Expression of RNA Silencing Genes

**Figure 9:**
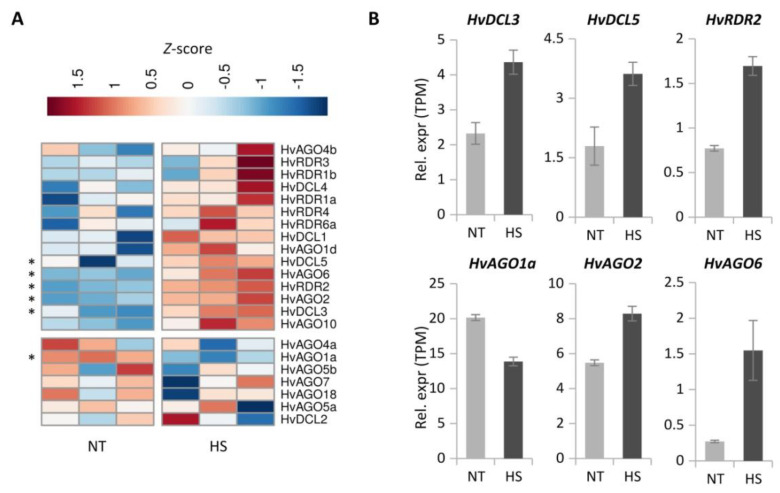
RNA-seq analysis of the data published by Pacak et al. [79]. (**A**) Heat map representation of the expression pattern of silencing-related genes in heat-shocked (HS) and not treated (NT) barley plants (data for the three biological replicates are shown separately). Colours represent *Z*-scores, which show how many standard deviations the given value is above or below the mean of all values in a row. Genes that are significantly differentially expressed between the NT and HS samples are marked with asterisks. (**B**) Bar chart representation of the expression values of the significantly differentially expressed RNA silencing-related genes. Expression values are normalised transcript per million (TPM) units. Error bars represent standard errors.

High-temperature stress (heat stress, HS) is considered to be one of the major abiotic stresses affecting both composition of natural habitats and distribution and productivity of agriculturally important plants worldwide [118,119,120]. Plants alter their developmental pathways to re-allocate resources and ensure versatile stress management. To preliminary assess the transcriptional alterations of RNA silencing components in response to HS, RNA-seq data of barley cv. Rolap shoots provided by Pacak et al. [79] was analysed. A heat-map was generated using the normalised expression values of every putative *HvDCL*, *HvAGO* and *HvRDR* gene (Figure 9A). According to the RNA-seq data, *HvDCL3, HvDCL5, HvAGO2, HvAGO6,* and *HvRDR2* were significantly induced, while *HvAGO1a* was slightly downregulated in heat-stressed shoots (Figure 9).

**Figure 10:**
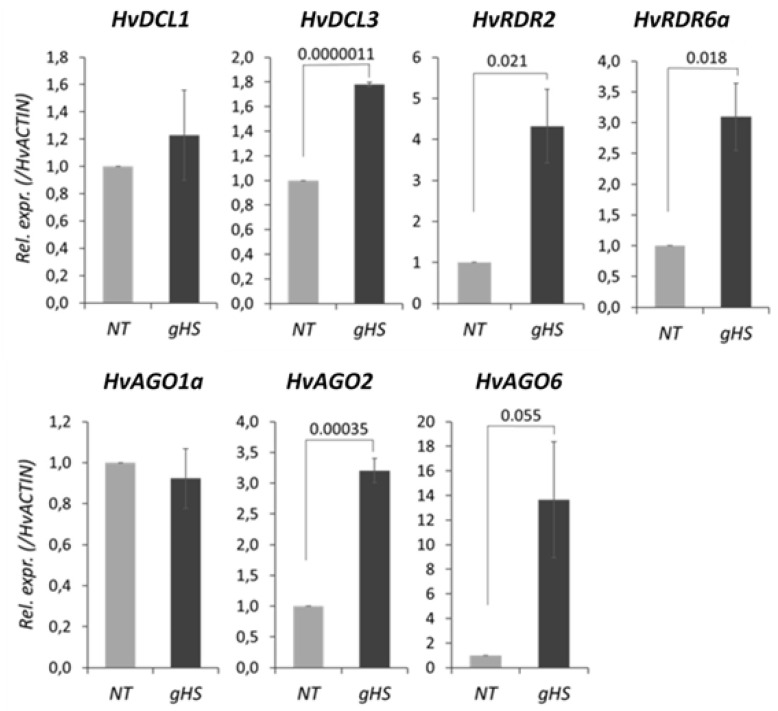
RT-qPCR analysis of selected RNA silencing factor transcripts in gradient heat-shock (gHS)-treated and non-treated (NT) barley leaves. *p*-values for significant changes are shown over the bars. For details, see Materials and Methods.

To verify the results of the in silico analysis, expression of selected silencing-related genes, including the ones that changed significantly upon heat stress in the RNA-seq experiment by Pacak et al. [79], were validated by RT-qPCR (Figure S7). Additionally, semi-quantitative PCR consistently measured elevated *HvDCL3, HvAGO6, HvRDR2* and *HvRDR6a* but unchanged levels of *HsDCL1* and *HvAGO1* following persistent, long term heat stress (40 °C/24 h) treatments (Figure S8). Finally, to mimic natural conditions, we performed the experiment by employing gradient heat shock treatment (gHS, 21 °C to 37 °C in the course of 4 h, see Materials and Methods). Significant accumulation of *HvDCL3*, *HvAGO2*, *HvAGO6*, *HvRDR2* and *HvRDR6a* transcripts was again certified. The two main factors of miRNA pathway *HvDCL1* and *HvAGO1a* however, were not altered by this treatment either (Figure 10).

In summary, moderate direct HS (35.5 °C/48 h), prolonged heat (40 °C/24 h) and gradient heat (21–37 °C/4 h) all lead to transcriptional accumulation of siRNA- but not miRNA-based pathway components (Table S8).

## 4. Discussion

Barley is a multi-purpose crop plant: besides its economic importance, is a model species for cereal research (almost 20,000 papers were published on barley based on a PubMed keyword search). As a close relative to wheat (separated ca. ten million years), it may be a good starting point for studies difficult to conduct in wheat. Fertile hybrids may also be produced between barley and wheat, enlarging the genetic utensils available. Importantly, barley is a more resilient plant and adapts better to regions/habitats where wheat cannot be cultivated. Due to its diploid genome, it is suitable for easy obtaining of mutants through classical breeding or molecular (e.g., CRISPR mutagenesis) tools. Therefore, understanding cellular pathways and their specific features in barley is more convenient and could give a good model for crop science. Since RNA silencing plays a crucial role in the life of plants, there is a growing need to identify and characterise its central protein components in crop plants like barley.

DCL proteins are key factors of sRNA biogenesis. Unlike mammals, which have only one DCL enzyme, plants possess at least a set of four DCLs of monophyletic origin. During evolution, more complex plants tend to evolve more DCLs as a result of gene duplication events [28]. The close phylogenetic relationship within DCL1, 2, 3 and 4 clade orthologues suggests functional conservation. Mild differences, however, exist within the *Hv*DCL and their orthologues: (i) The domain organisation of *Hv*DCL1 is very similar to the *At*DCL1, while in rice and maize DCL1 PAZ and Dicer-dimer domains are slightly altered. This change may have occurred after the divergence of the common ancestor of wheat and barley from the ancestor of rice and maize (ca. 60 million years ago) [28]; (ii) PAZ domain within monocot DCL2s (including barley) has a potentially modified structure, suggesting a distinct folding and RNA binding; (iii) when analysed, we found a highly conserved DCL catalytic core within all DCLs (Figure 5). However, dsRNA binding may be possibly different between DCL orthologs/paralogs; (iv) in contrast to dicots, monocots evolved a fifth DCL, DCL5 [28]. DCL5 is required for the production of specific 24-nt-long pha-siRNAs in reproductive tissues of rice [30]. In maize, the absence of DCL5 causes temperature-sensitive male sterility [121]. The presence and domain conservation of *Hv*DCL5 suggests a conserved and similar function in barley. Based on the roles of 24-nt siRNAs in *A. thaliana*, we speculate that perhaps DCL5 (and the 24-nt pha-siRNAs) may be involved in TGS during reproduction in barley. To reveal the functionally important differences in DCLs between dicots and monocots or within monocot lineages, further *in silico,* biochemical and genetic analyses are required.

AGO proteins are present in all eukaryotic organisms and can be identified by the combined presence of PAZ and PIWI domains [85]. The *A. thaliana*, rice, maize, and *B. distachyon* genomes encode 9 [39], 19 [25], 18 [26], and 16 [27] *AGO* genes, respectively. In wheat, two *AGO* genes have been described so far [122]. We describe here eleven *AGO* candidates in barley. Interestingly, the expansion of AGO family characteristic to monocots is not observed in barley. Although barley has one of the largest diploid genome (ca. 5.3 Gb), it seems that the restricted number of *Hv*AGOs are able to fulfil all tasks required. Existence of functionally distinct AGO clades seems to widely spread in plants. From these clades, the AGO1/5/10 contains the so-called binder and slicers, while the AGO4/6/8/9 the modifiers. It was suggested that the AGO2/3/7 clade members are potentially more flexible, and could provide both binder/slicer and modifier functions, depending on the circumstances [123].

In barley, we have found five members of AGO1/5/10 clade. *HvAGO1* and *HvAGO5* have been duplicated. Grasses in general exhibit an expanded AGO1/5/10 clade [87]. *Os*AGO1 family possess the catalytic residues and have slicer activity [100]. We have also found perfect conservation of DEDH + H motifs in *Hv*AGO1a, *Hv*AGO1d and *Hv*AGO5a but not in *Hv*AGO5b, where the histidine residue (analogous to *At*AGO1-H798, polar, hydrophobic) was replaced by a proline (non-polar, hydrophilic) (DEDH + P, Figure 6). It was shown experimentally that H798P change turns *At*AGO1 cleavage-deficient [35]. At 798 position a proline (P) residue is present in *At*AGO6, raising the possibility that *Hv*AGO5b also acts as a chromatin modifier. The observation that *Os*AGO5c/MEL1 is required for H3 modification reprogramming during male meiosis in rice supports this idea [124]. AGO10 has a particular function in *A. thaliana*: it sequesters miR165/166 to block its activity and enhance its decay. *At*AGO10 is required for stem cell maintenance in SAM [125,126]. Whether this specialised role of AGO10 is preserved in monocots is not known. The catalytic core (DEDH + Y) of *Hv*AGO10 was unique amongst clade members (*At*AGO10 has DEDH + H). Although tyrosine (Y) is similar to histidine (H), as both having aromatic and hydrophobic side chains, the absence of the charge (H has a positive charge at physiological pH) may alter the activity of *Hv*AGO10. Overall, we postulate that within *Hv*AGO1/5/10 clade, *Hv*AGO1a and 1b may act canonically and redundantly, while *Hv*AGO5a, *Hv*AGO5b and *Hv*AGO10 probably perform differentiated/ specialised functions.

Grasses have evolved a further subgroup of AGO1/5/10, the AGO18 clade (Figure 3), that seems to be required for specific tasks: AGO18 confers broad virus-resistance in rice [127]; on the other hand, it was proposed that it binds 24-nt pha-siRNAs to regulate male reproductive organ development [128]. The phylogenetic analysis of *Hv*AGO18 placed it close to *Z. mays* and *O. sativa* AGO18 ortholog proteins, implying similar functions.

*At*AGO2 of the AGO2/3/7 clade binds 21-nt sRNAs and was shown to have roles during pathogen defence and double-stranded DNA repair [33,116,129,130]. In spite of the fact that *At*AGO2 and *At*AGO3 are very similar, surprisingly, *At*AGO3 binds 24-nt and targets transposable elements through TGS [131]. In barley, only the homolog of AGO2 has been detected. *Hv*AGO2 also contained the distinctive DEDD + H motif specific to *At*AGO2/3 proteins (Figure 6). Which AGO may substitute for AGO3 activity in barley, is an exciting question. The function of the second member of the clade AGO7 is more conserved: it regulates organ development through miR390 binding and generation of ta-siRNAs from TAS3 precursor [100,132]. Domain organisation and catalytic core in *Hv*AGO7 are similar to *At*AGO7 (Figure 3). As multiple TAS3 loci have been found in barley [133], we assume conserved functions for *Hv*AGO7.

The modifier AGO clade (AGO4/6/8/9) contains three functional members in *A. thaliana* (*AtAGO8* is a pseudogene), with partial redundancy and specificity [134,135,136]. The clade members bind primarily 24-nt hc-siRNAs with 5′-A but also 21-nt ta-siRNAs to direct RdDM [17,137,138]. Barley genome also encodes three members of this clade, *HvAGO4a*, *4b* and *6*. The DEDH + P catalytic core of *Hv*AGO4a, 4b and 6 is identical to *At*AGO6 (Figure 6B). Domain topology and phylogenetic analysis of *Hv*AGO shows high similarity to *A. thaliana*, rice and maize clade members suggesting chromatin regulatory roles. The observation that *Zm*AGO104 (*Zm*AGO9) is needed for non-CG methylation of centromere region and knot-repeat DNA backs this theory [139]. We could not define in silico the functional homolog of AGO9. *At/Zm*AGO9 proteins are predominantly expressed in ovules and regulate cell fate [136,139]. Which of barley AGO4/6/8/9 clade members fulfils this task remains a future question.

Initially, RDR proteins were studied for their antiviral roles but later it became evident that they are also required during gene expression control and chromatin regulation. Several barley *Hv*RDR genes were identified previously [83], however, we expanded this gene family with systematic identification of multiple members (Figure 1 and Table 1). We noted a slight expansion of RDRα (5 members) and a reduction of RDRγ (2 members) clades. Phylogenetic analysis, domain organisation and catalytic core type reinforce their classification (Figure 1 and Figure 4). There are only scattered data on RDRα protein functions in monocots: *Os*RDR1 is induced and required for antiviral defence [140]; maize RDR2 ortholog MOP1 takes part in heritable chromatin silencing [141]; SHL2, 4 and SHO1 proteins (RDR6 orthologs) are involved in ta-siRNA pathway in rice [142]. The close phylogenetic relationship and available functional data suggest conserved functions of RDR1, 2 and 6 in barley. The existence of two *Hv*RDR1 and *Hv*RDR6 homologues suggests an evolutionary selection for specialisation either during development or under stress responses. Indeed, *HvRDR1a/b* and *HvRDR6a/b* transcription were selectively induced in different organs, in response to different pathogens or under elevated temperature [83]. On the other hand, the presence of the two RDRγ proteins (*Hv*RDR3, 4) informs about a need for their activity. Absence of one or multiple RDR3, 4, and 5 proteins from different plants, however, suggests non-essential roles [143].

To function in an equilibrated and flexible manner, RNA silencing needs to be strictly regulated at multiple layers including miRNA-directed feedback loops. Several mechanisms have been described, including miR168/AGO1, miR403/AGO2/3, miR162/DCL1 feedback loops [109,110,116,117]. In monocots, multiple loci of the miR168 family are present suggesting that *AGO1s* self-regulation occurs in monocots as well [113]. In our study, miR168 target sites were in silico detected in both *HvAGO1a* and *1d* transcripts, implying that miR168-mediated regulation potentially occur in barley (Figure 8) [115]. *AGO1* regulation by miR168 was also proposed in maize [114]. The presence of miR168 and that of target sites within *HvAGO1a* and *1b*, nonetheless, is not the ultimate proof [144], therefore miR168-mediated self-regulation in monocots lacks the final biochemical evidence. miR162/*DCL1* and miR403/*AGO2*/*3* feedback regulation [116,117] seems to be absent from barley. Other components of RNA silencing (e.g., RDRs) may also be regulated. The monocot-specific miR444 indirectly activates *Os*RDR1 to boost antiviral silencing response [140]. MiR444 is also present in barley, raising the possibility of a similar regulation [115].

Heat stress is a major threat to barley crop yield and quality. Previous studies demonstrated the heat-regulated changes of specific miRNAs, ta-siRNAs, hc-siRNAs despite the rather stable global level of sRNAs [53,54,58,145,146,147,148]. Transcription of silencing *trans* factor is also modulated by heat. *Solanum lycopersicum AGO10a* and *AGO10b (SlAGO10a* and *SlAGO10b)* were both activated by heat [65]. *At*AGO1 is required for heat-stress memory [56]. Prolonged elevated temperature releases transgene-induced PTGS, which was epigenetically inherited trans-generationally [145]. These data show an intimate connection between environmental temperature, sRNA biogenesis/activity, silencing *trans* factor regulation and epigenetic/chromatin reprogramming.

To unravel heat-induced transcriptional regulation of silencing *trans* factors in barley, we studied RNA-seq data available [79] (Figure 9). Based on these, we selected silencing factors and assessed their change during three different heat stress regimes, including moderate HS (35.5 °C/48 h), prolonged heat (40 °C/24 h) and gradient elevation of heat (21–37 °C/4 h). Significant accumulation of *HvDCL3*, *HvAGO2*, *HvAGO6*, *HvRDR2* and *HvRDR6a* mRNA was confirmed (Figure 9, Figure 10, Figures S7 and S8). The highly conserved miR390/TAS3/ARF pathway exerts its function via the siRNA-based subgroup of silencing factors. We searched for TAS3-derived tasiRNA targets in barley and identified three potential ARF target transcripts. Two of these targets were significantly down-regulated upon heat stress (Figure S9) suggesting that the activity of siRNA-based silencing is potentially elevated at higher temperature. Contrarily, the principal *trans* factors of miRNA pathway, *HvDCL1* and *HvAGO1a* seem to be much stable under the investigated circumstances. In summary, data from RNA-seq, semi-quantitative and RT-qPCR measurements all converge and points towards the transcriptional accumulation of factors enrolled primarily in siRNA-based silencing, including hc-siRNA, pha-siRNA and RDR6-dependent sRNA pathways. Importantly, our gradient heat treatment mimics natural situations, e.g., temperature changes during a summer day, therefore may be relevant in field conditions. As DCL3, AGO2, AGO6, RDR2 and RDR6 factors were all involved in TGS or double-stranded DNA break repair [130,137,149], RNA silencing could have chromatin regulatory and protective roles in barley during heat stress acclimation.

## 5. Conclusions

We have identified members of gene families having key roles in RNA silencing of barley and provided basic data on their genomic location, clade, phylogenetic relations, domain and motif organisation, and catalytic core build-up. Our data firmly suggests that these players are potentially functional and likely required at some point during barley’s lifecycle. Transcriptional accumulation of siRNA pathway factors hints to a probable role in environmental adaptation. This work will be a stepping-stone to ask further fundamental and exciting questions that remained pending in barley and monocot RNA silencing field.

## Figures and Tables

**Table 1:** Features of predicted *H. vulgare* DCL, AGO and RDR homologues.

Predicted Gene Name *	Chromosomal Location (5′-3′)	Accession No. of Ensembl Transcript	ORF Length (bp)	No. Exons	No. of Amino Acids	Isoelectric Point (PI) **	Molecular Weight (Da) **
***HvDCLs***							
*HvDCL1*	chr4H:630334902–630347021	HORVU4Hr1G084890.7	5994	19	1997	6.26	222,185.89
*HvDCL2*	chr5H:84450148–84493265	HORVU5Hr1G019300.8	4428	20	1475	8.56	165,696.76
*HvDCL3*	chr3H:638064590–638074096	HORVU3Hr1G092430.9	5058	26	1685	5.92	188,438.84
*HvDCL4*	chr2H:632322356–632371458	HORVU2Hr1G088270.9	4908	25	1635	6.17	185,174.48
*HvDCL5*	chr1H:310007028–310025536	HORVU1Hr1G042710.5	5052	26	1683	6.21	189,830.24
***HvAGOs***							
*HvAGO1a*	chr7H:9459266–9467181	HORVU7Hr1G007000.14	3654	23	1217	9.56	133,685.87
*HvAGO1d*	chr7H:651899946–651906855	HORVU7Hr1G120600.3	3195	22	1064	9.05	117,524.50
*HvAGO2*	chr2H:684121508–684127760	HORVU2Hr1G098650.1	3144	3	1047	9.34	113,039.59
*HvAGO4a*	chr3H:226356734–226364526	HORVU3Hr1G038830.1	2769	23	922	9.10	102,605.58
*HvAGO4b*	chr7H:623895748–623891202	HORVU7Hr1G107770.0 ***	2451	22	816	4.88	201,998.97
*HvAGO5a*	chr4H:8972435–8981437	HORVU4Hr1G004030.1	3102	22	1033	9.47	114,317.97
*HvAGO5b*	chr2H:116787397–116793928	HORVU2Hr1G031130.2	2532	22	843	9.17	94,171.10
*HvAGO6*	chrUn:71291842–71302762	HORVU0Hr1G012490.1	2652	23	883	9.25	98,208.02
*HvAGO7*	chrUn:29969371–29973587	HORVU0Hr1G005350.1	3039	3	1012	9.28	114,045.18
*HvAGO10*	chr6H:183574909–183587426	HORVU6Hr1G036840.2	2850	21	949	9.43	105,648.60
*HvAGO18*	chr3H:68596766–68603419	HORVU3Hr1G021290.1	3156	21	1051	9.17	114,177.28
***HvRDRs***							
*HvRDR1a*	chr6H:512834480–512842253	HORVU6Hr1G074180.1	3348	5	1115	7.73	126,707.04
*HvRDR1b*	chr6H:513108807–513120207	HORVU6Hr1G074220.2	3549	5	1182	8.66	134,118.47
*HvRDR2*	chr2H:589496751–589507376	HORVU2Hr1G081260.6	3414	4	1137	6.80	127,637.79
*HvRDR3*	chr3H:116156819–116191312	HORVU3Hr1G027290.6	3588	19	1195	6.30	134,565.32
*HvRDR4*	chr3H:116503595–116535566	HORVU3Hr1G027340.4	3507	19	1168	6.83	131,898.43
*HvRDR6a*	chr3H:673186268–673191806	HORVU3Hr1G107690.2	3684	2	1227	7.38	137,814.98
*HvRDR6b*	chr3H:17012603–17027858	HORVU7Hr1G012280.1	3621	6	1206	8.17	135,686.38

* Predicted gene name according to phylogenetic analyses. *H. vulgare* RNA silencing components were named after their closest *A. thaliana* relative. For AGO1/5/10- and AGO18-clade proteins of barley, monocotyledonous *O. sativa* and *Z. mays* were the base of the nomenclature. ** Predicted values calculated by ExPasy. *** This transcript variant is not in the official ENSEMBL annotation; therefore, it was provisionally named HORVU7Hr1G107770.0 variant.

## Supplementary Materials

The following are available online at https://www.mdpi.com/2218-273X/10/6/929/s1, Figure S1: Full-length protein alignment of all *A. thaliana* and barley DCL proteins, Figure S2: Full-length protein alignment of all *A. thaliana* and barley AGO proteins, Figure S3: Full-length protein alignment of all *A. thaliana* and barley RDR proteins, Figure S4: Partial alignment of *A. thaliana* and barley DCLs’ PAZ domains, Figure S5: Partial alignment of *A. thaliana* and barley AGOs’ PAZ domains, Figure S6: Partial alignment of *A. thaliana* and barley AGOs’ PIWI domains, Figure S7: RT-qPCR analysis of selected RNA silencing transcripts in response to heat treatment (40 °C/24 h; HS) and non-treated (NT) barley leaves, Figure S8: Semi-quantitative RT-PCR measurement of barley RNA silencing factor expressional changes in response to sustained heat stress (40 °C/24 h), Figure S9: Targets of the conserved TAS3-derived siRNA in barley, Supplemental Material 1: Identity and Similarity scores of *A. thaliana*, *O. sativa*, *Z. mays* and *H. vulgare* homologue proteins, Table S1: List of primers used for semiquantitative PCR, Table S2: List of primers used for RT-qPCR, Table S3: Number of exons in barley RNAi genes and their *Arabidopsis* homologues, Table S4: Domain organisation of barley RNAi-related proteins according to Pfam, Table S5: Conserved motifs of *Hv*DCLs identified by MEME, Table S6: Conservative motifs of HvAGOs identified by MEME, Table S7: Conserved motifs of HvRDRs identified by MEME.
