# Supplementary material for: Genome-Wide Identification of RNA Silencing-Related Genes and Their Expressional Analysis in Response to Heat Stress in Barley (Hordeum vulgare L.)
Source: Biomolecules. 2020 Jun 18;10(6):929. doi: 10.3390/biom10060929 (PMC7356095; doi:10.3390/biom10060929)
Supplement: Supplementary file 1 [file biomolecules-10-00929-s001.zip › Figure_S1-9.pdf]

Figure S1

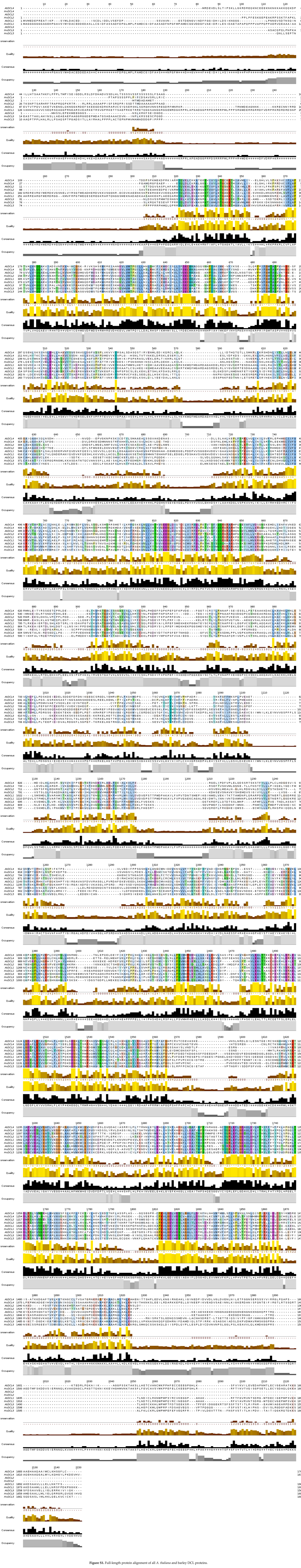

Figure S1. Full-length protein alignment of all *A. thaliana* and barley DCL proteins.

Figure S2

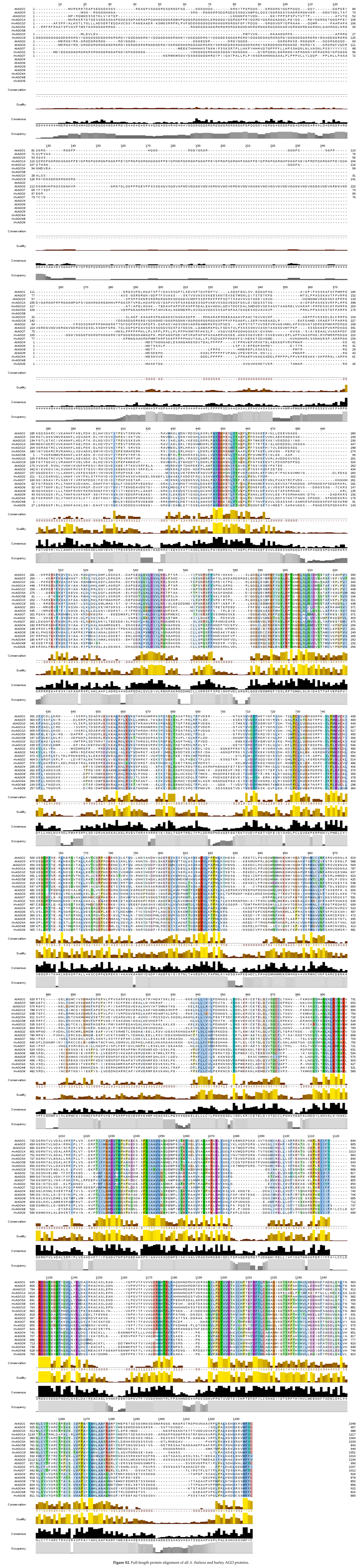

Figure S2. Full-length protein alignment of all *A. thaliana* and barley AGO proteins.

Figure S3

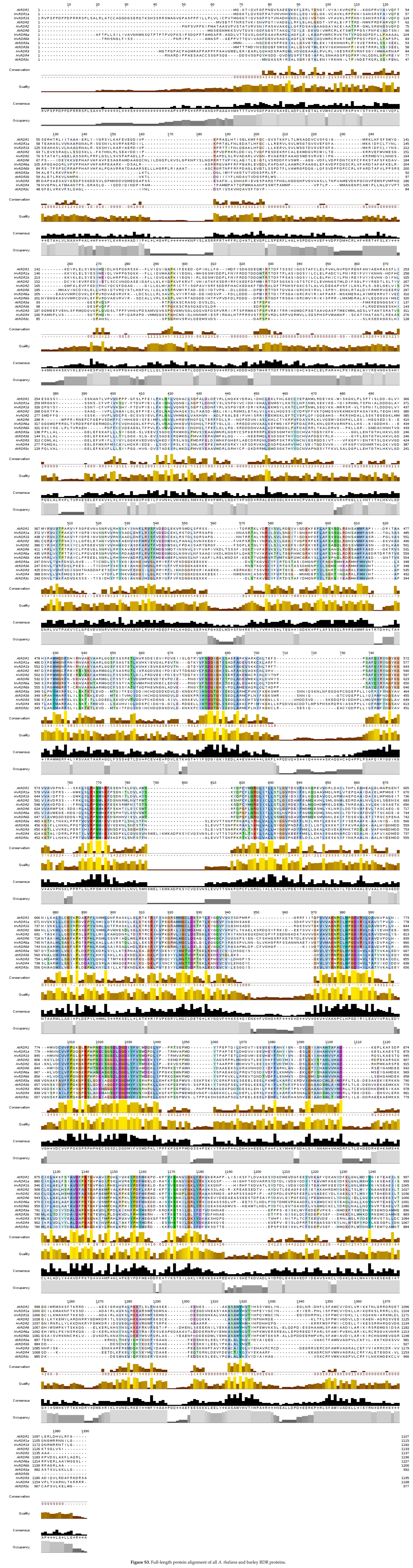

Figure S3. Full-length protein alignment of all *A. thaliana* and barley RDR proteins.

Figure S4

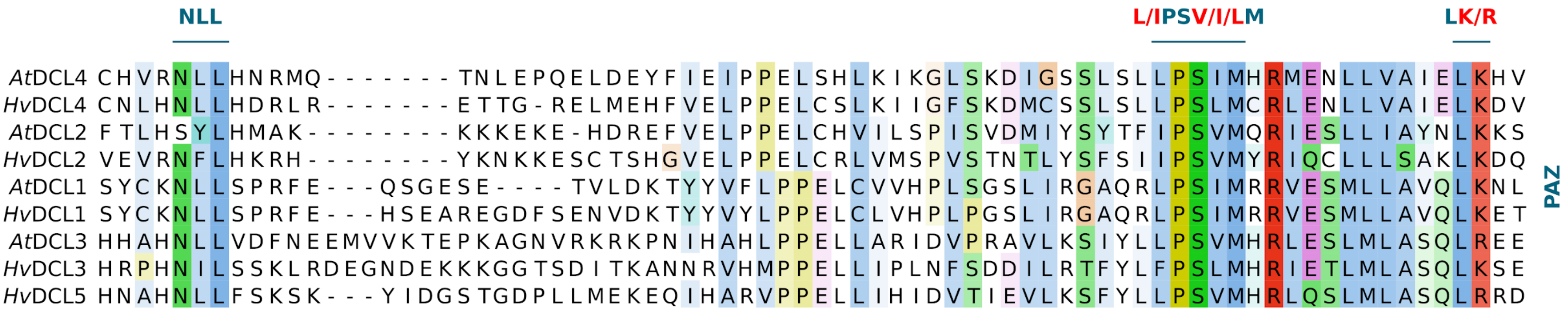

Figure S4. Partial alignment of *A. thaliana* and barley DCLs' PAZ domains. N-terminal part of PAZ-loop (NLL motif) responsible for dsRNA binding and Connector helix core L/IPSI/L/MM$(X)_{11}$LK/R is conserved between *A. thaliana* and barley.

Figure S5

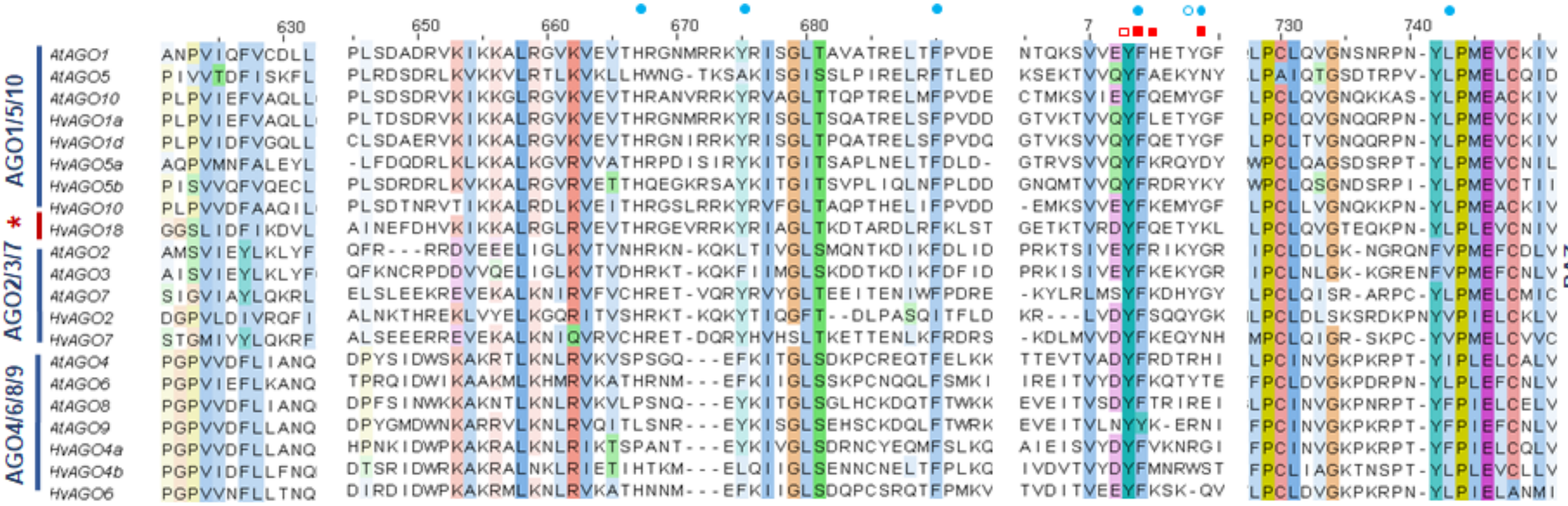

Figure S5. Partial alignment of *A. thaliana* and barley AGOs' PAZ domains. Amino acids involved in the sRNA 3' end binding; residues corresponding to *Dm*AGO1 aromatic cluster $\alpha3$ (L, Y, F and Y, red squares, based on Yan *et al.* [92]) or human *Hs*AGO-eiF2c1 (H, Y, F, Y and L, blue circles, based on Ma *et al.* [93]) are highlighted in *A. thaliana* and/or barley AGOs. Numbers correspond to residues of *At*AGO1. For details see *Materials and Methods*.

Figure S6

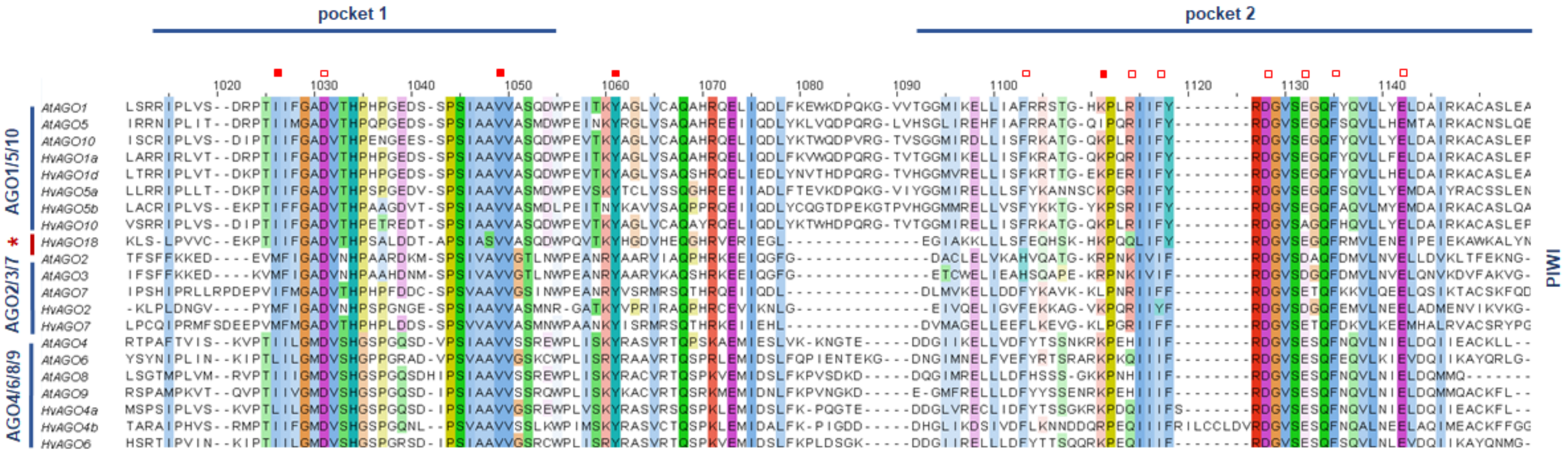

Figure S6. Partial alignment of *A. thaliana* and barley AGOs' PIWI domains. The tandem tryptophan-pockets within the PIWI domains of *A. thaliana* and barley AGOs were determined using the paper by Till *et al.* [106]; red boxes denote conserved residues between *At*AGO1 and *Hs*AGO2, the mutation of which give loss-of-function phenotype; open boxes show conserved residues between *At*AGO1 and *Hs*AGO2 in pocket regions. Numbers correspond to residues of *At*AGO1. For details see *Materials and Methods*.

Figure S7

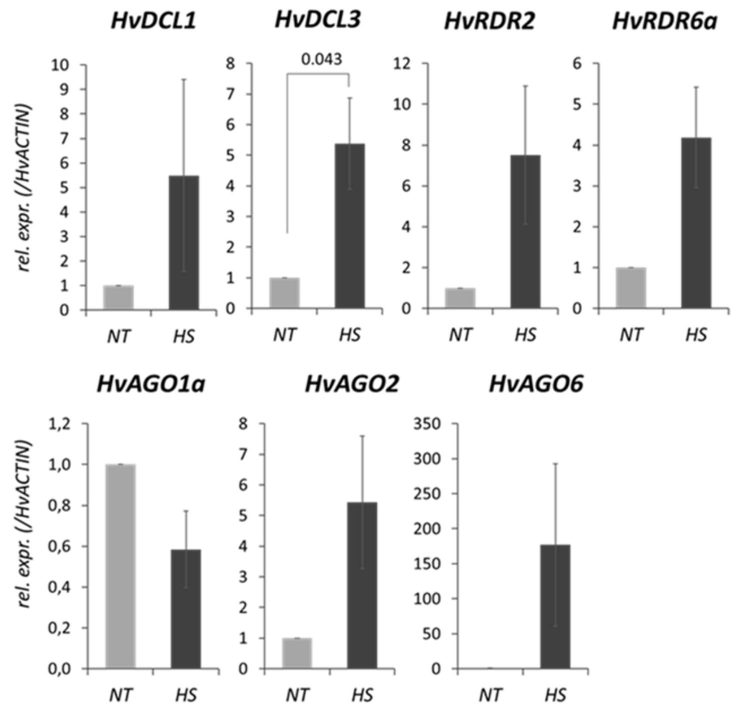

Figure S7. RT-qPCR analysis of selected RNA silencing factor transcripts in response to heat treatment (40°C/24h; HS) and non-treated (NT) barley leaves. *P*-values for significant changes are shown over the bars. For details, see *Materials and methods*.

Figure S8

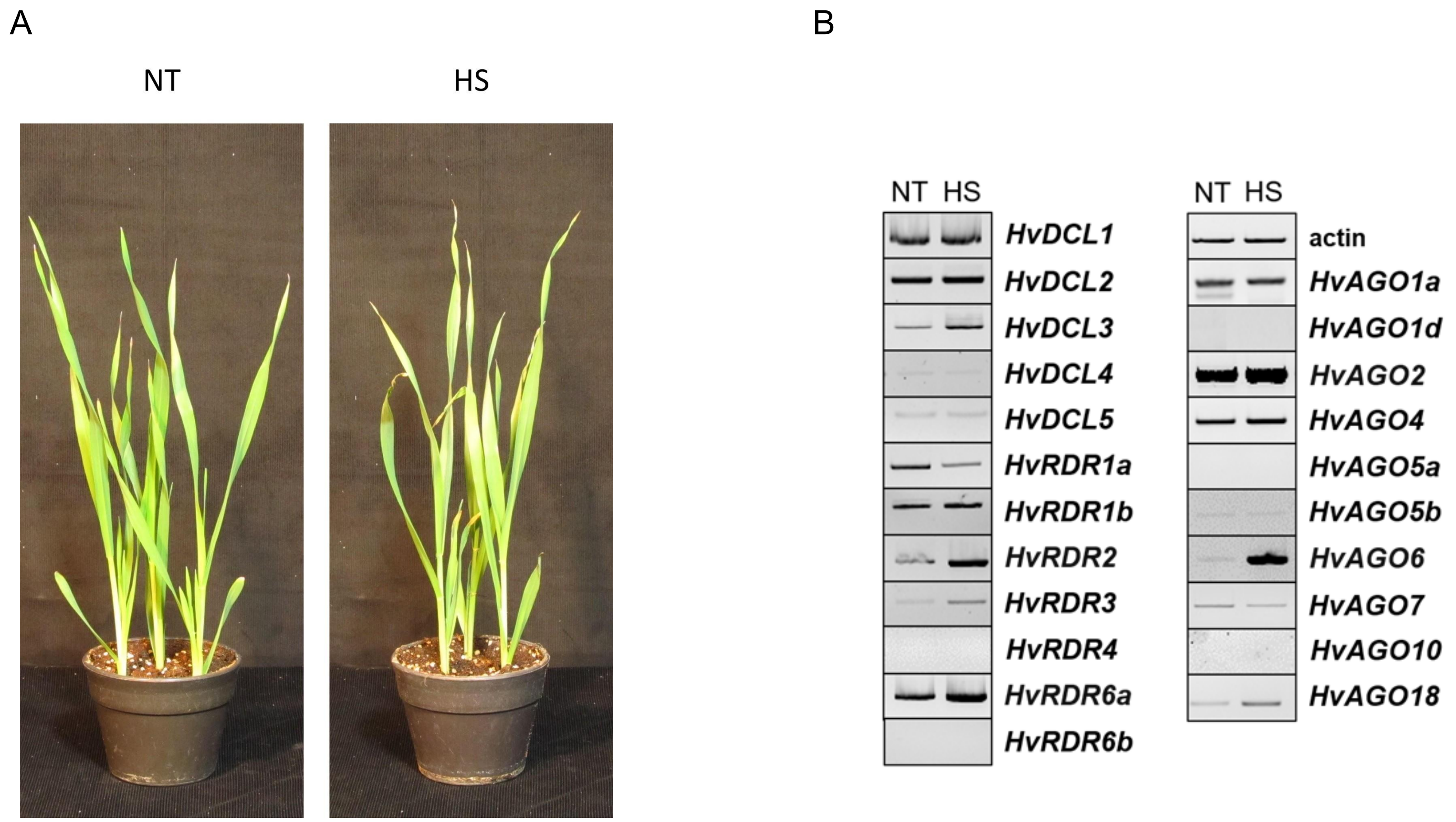

Figure S8. Semi-quantitative RT-PCR measurement of barley RNA silencing factor expressional changes in response to sustained heat stress (40°C/24h). (**A**) Temperature-dependent lesion phenotype is observed on barley seedlings one week following heat treatments (Non-treated, NT; heat stress treated, HS. (**B**) Semi-quantitative RT-PCR analysis of barley RNA silencing factors (for details see *Materials and methods*).

Figure S9

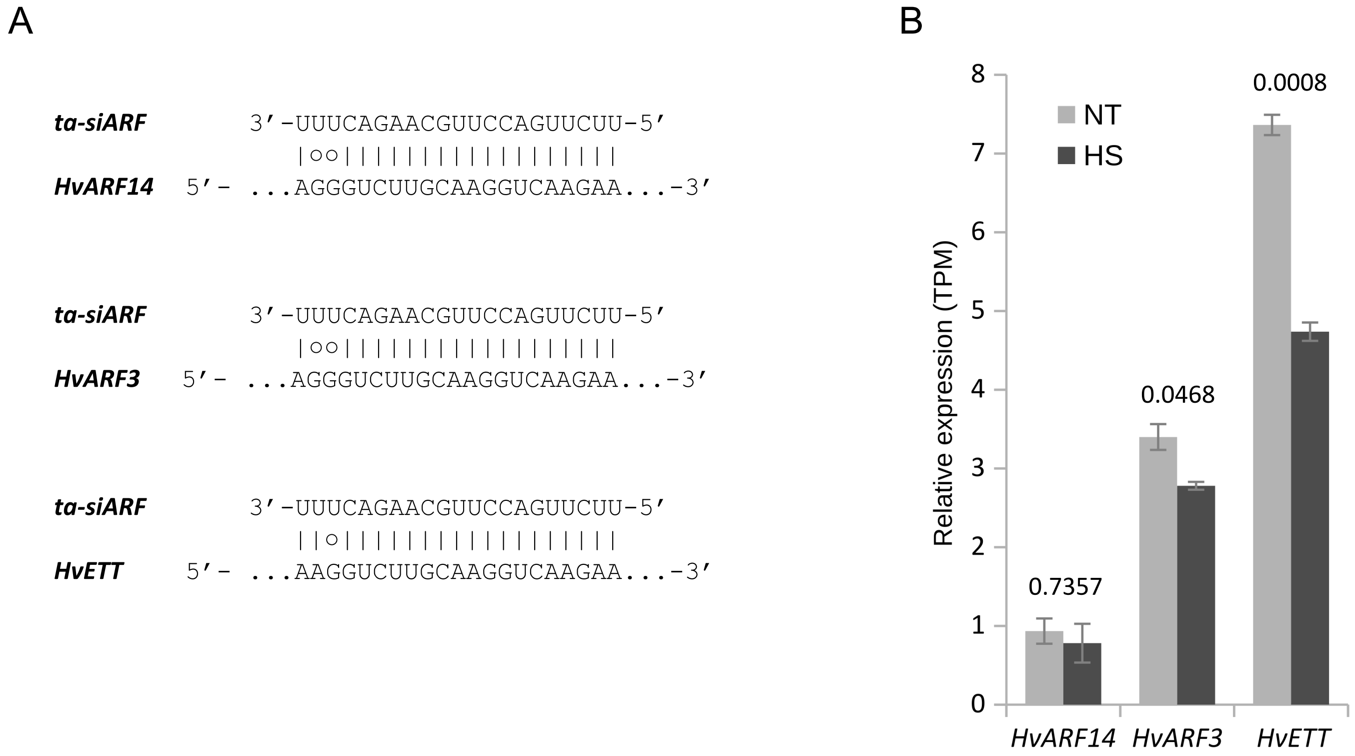

Figure S9. Targets of the conserved TAS3-derived ta-siARF in barley. (**A**) Structure of the ta-siRNA/target duplexes predicted by the psRNATarget server. The IDs of the target genes are the following: *HvARF14* (HORVU1Hr1G076690), *HvARF3* (HORVU3Hr1G072340), *HvETT* (HORVU1Hr1G087460). (**B**) Expression of ta-siARF targets upon heat stress based on the RNA-seq data by Pacak *et al.* [79]. A Student's t-test was performed to test if there is a difference between the means of the not treated (NT) and heat-shocked (HS) plant samples (n = 3). The error bars represent standard error. The *P*-values are indicated over the bars.
