# Supplementary material for: Genome-Wide Identification of RNA Silencing-Related Genes and Their Expressional Analysis in Response to Heat Stress in Barley (Hordeum vulgare L.)
Source: Biomolecules. 2020 Jun 18;10(6):929. doi: 10.3390/biom10060929 (PMC7356095; doi:10.3390/biom10060929)
Supplement: Supplementary file 1 [file biomolecules-10-00929-s001.zip › Supplementary_material_1.pdf]

Ident and Sim results

Results for ZmAGO1E vs ZmAGO1A :
Alignment length: 1163
Identical residues: 993
Similar residues: 35
Percent identity: 85.38
Percent similarity: 88.39

Results for ZmAGO1E vs ZmAGO1C :
Alignment length: 1171
Identical residues: 913
Similar residues: 39
Percent identity: 77.97
Percent similarity: 81.30

Results for ZmAGO1E vs ZmAGO1D :
Alignment length: 1204
Identical residues: 674
Similar residues: 104
Percent identity: 55.98
Percent similarity: 64.62

Results for ZmAGO1E vs ZmAGO2 :
Alignment length: 1164
Identical residues: 268
Similar residues: 143
Percent identity: 23.02
Percent similarity: 35.31

Results for ZmAGO1E vs ZmAGO7 :
Alignment length: 1163
Identical residues: 277
Similar residues: 146
Percent identity: 23.82
Percent similarity: 36.37

Results for ZmAGO1E vs ZmAGO5B :
Alignment length: 1156
Identical residues: 497
Similar residues: 144
Percent identity: 42.99
Percent similarity: 55.45

Results for ZmAGO1E vs ZmAGO5C :
Alignment length: 1156
Identical residues: 303
Similar residues: 133
Percent identity: 26.21
Percent similarity: 37.72

Results for ZmAGO1E vs ZmAGO10B :
Alignment length: 1126
Identical residues: 649
Similar residues: 84
Percent identity: 57.64
Percent similarity: 65.10

Results for ZmAGO1E vs ZmAGO18A :
Alignment length: 1169
Identical residues: 478
Similar residues: 121
Percent identity: 40.89
Percent similarity: 51.24

Results for ZmAGO1E vs ZmAGO4D :
Alignment length: 1162
Identical residues: 295
Similar residues: 154
Percent identity: 25.39
Percent similarity: 38.64

Results for ZmAGO1E vs ZmAGO5A :
Alignment length: 1142
Identical residues: 524
Similar residues: 149
Percent identity: 45.88
Percent similarity: 58.93

Results for ZmAGO1E vs ZmAGO18B :
Alignment length: 1194
Identical residues: 381
Similar residues: 143
Percent identity: 31.91
Percent similarity: 43.89

Results for ZmAGO1E vs OsAGO1A :
Alignment length: 1138
Identical residues: 861
Similar residues: 52
Percent identity: 75.66
Percent similarity: 80.23

Results for ZmAGO1E vs OsAGO1B :
Alignment length: 1145
Identical residues: 931
Similar residues: 36
Percent identity: 81.31
Percent similarity: 84.45

Results for ZmAGO1E vs OsAGO1C :
Alignment length: 1122
Identical residues: 715
Similar residues: 79
Percent identity: 63.73
Percent similarity: 70.77

Results for ZmAGO1E vs OsAGO1D :
Alignment length: 1120
Identical residues: 716
Similar residues: 97
Percent identity: 63.93
Percent similarity: 72.59

Results for ZmAGO1E vs OsAGO2 :
Alignment length: 1202
Identical residues: 299
Similar residues: 157
Percent identity: 24.88
Percent similarity: 37.94

Results for ZmAGO1E vs OsAGO3 :
Alignment length: 1252
Identical residues: 273
Similar residues: 149
Percent identity: 21.81
Percent similarity: 33.71

Results for ZmAGO1E vs OsAGO4A :
Alignment length: 1160
Identical residues: 297
Similar residues: 148
Percent identity: 25.60
Percent similarity: 38.36

Results for ZmAGO1E vs OsAGO4B :
Alignment length: 1162
Identical residues: 308
Similar residues: 145
Percent identity: 26.51
Percent similarity: 38.98

Results for ZmAGO1E vs OsAGO7 :
Alignment length: 1188
Identical residues: 327
Similar residues: 152
Percent identity: 27.53
Percent similarity: 40.32

Results for ZmAGO1E vs OsAGO11 :
Alignment length: 1133
Identical residues: 433
Similar residues: 119
Percent identity: 38.22
Percent similarity: 48.72

Results for ZmAGO1E vs OsAGO12 :
Alignment length: 1140
Identical residues: 512
Similar residues: 143
Percent identity: 44.91
Percent similarity: 57.46

Results for ZmAGO1E vs OsAGO13 :
Alignment length: 1490
Identical residues: 457
Similar residues: 109
Percent identity: 30.67
Percent similarity: 37.99

Results for ZmAGO1E vs OsAGO14 :
Alignment length: 1157
Identical residues: 511
Similar residues: 138
Percent identity: 44.17
Percent similarity: 56.09

Results for ZmAGO1E vs OsAGO15 :
Alignment length: 1165
Identical residues: 301
Similar residues: 127
Percent identity: 25.84
Percent similarity: 36.74

Results for ZmAGO1E vs OsAGO16 :
Alignment length: 1156
Identical residues: 293
Similar residues: 134
Percent identity: 25.35
Percent similarity: 36.94

Results for ZmAGO1E vs OsAGO17 :
Alignment length: 1118
Identical residues: 465
Similar residues: 110
Percent identity: 41.59
Percent similarity: 51.43

Results for ZmAGO1E vs OsAGO18 :

Alignment length: 1186
Identical residues: 460
Similar residues: 131
Percent identity: 38.79
Percent similarity: 49.83

Results for ZmAGO1E vs OsMEL1 :
Alignment length: 1139
Identical residues: 553
Similar residues: 125
Percent identity: 48.55
Percent similarity: 59.53

Results for ZmAGO1E vs OsPNH1 :
Alignment length: 1130
Identical residues: 657
Similar residues: 89
Percent identity: 58.14
Percent similarity: 66.02

Results for ZmAGO1E vs AtAGO1 :
Alignment length: 1123
Identical residues: 776
Similar residues: 69
Percent identity: 69.10
Percent similarity: 75.24

Results for ZmAGO1E vs AtAGO2 :
Alignment length: 1192
Identical residues: 296
Similar residues: 161
Percent identity: 24.83
Percent similarity: 38.34

Results for ZmAGO1E vs AtAGO3 :
Alignment length: 1272
Identical residues: 293
Similar residues: 171
Percent identity: 23.03
Percent similarity: 36.48

Results for ZmAGO1E vs AtAGO4 :
Alignment length: 1163
Identical residues: 304
Similar residues: 159
Percent identity: 26.14
Percent similarity: 39.81

Results for ZmAGO1E vs AtAGO5 :
Alignment length: 1135
Identical residues: 522
Similar residues: 127
Percent identity: 45.99
Percent similarity: 57.18

Results for ZmAGO1E vs AtAGO6 :
Alignment length: 1154
Identical residues: 308
Similar residues: 134
Percent identity: 26.69
Percent similarity: 38.30

Results for ZmAGO1E vs AtAGO7 :
Alignment length: 1173
Identical residues: 325
Similar residues: 137
Percent identity: 27.71
Percent similarity: 39.39

Results for ZmAGO1E vs AtAGO8 :
Alignment length: 1145
Identical residues: 281
Similar residues: 125
Percent identity: 24.54
Percent similarity: 35.46

Results for ZmAGO1E vs AtAGO9 :
Alignment length: 1155
Identical residues: 298
Similar residues: 156
Percent identity: 25.80
Percent similarity: 39.31

Results for ZmAGO1E vs AtAGO10 :
Alignment length: 1132
Identical residues: 652
Similar residues: 93
Percent identity: 57.60
Percent similarity: 65.81

Results for ZmAGO1E vs HvAGO4A :
Alignment length: 1164
Identical residues: 303
Similar residues: 143
Percent identity: 26.03
Percent similarity: 38.32

Results for ZmAGO1E vs HvAGO4B :
Alignment length: 1163
Identical residues: 277
Similar residues: 131
Percent identity: 23.82
Percent similarity: 35.08

Results for ZmAGO1E vs HvAGO7 :
Alignment length: 1170
Identical residues: 320
Similar residues: 147
Percent identity: 27.35
Percent similarity: 39.91

Results for ZmAGO1E vs HvAGO1D :
Alignment length: 1163
Identical residues: 687
Similar residues: 106
Percent identity: 59.07
Percent similarity: 68.19

Results for ZmAGO1E vs HvAGO5B :
Alignment length: 1128
Identical residues: 491
Similar residues: 112
Percent identity: 43.53
Percent similarity: 53.46

Results for ZmAGO1E vs HvAGO6 :
Alignment length: 1156
Identical residues: 299
Similar residues: 130
Percent identity: 25.87
Percent similarity: 37.11

Results for ZmAGO1E vs HvAGO2 :
Alignment length: 1197
Identical residues: 309
Similar residues: 152
Percent identity: 25.81
Percent similarity: 38.51

Results for ZmAGO1E vs HvAGO18 :
Alignment length: 1159
Identical residues: 484
Similar residues: 130
Percent identity: 41.76
Percent similarity: 52.98

Results for ZmAGO1E vs HvAGO5A :
Alignment length: 1139
Identical residues: 510
Similar residues: 158
Percent identity: 44.78
Percent similarity: 58.65

Results for ZmAGO1E vs HvAGO10 :
Alignment length: 1124
Identical residues: 597
Similar residues: 105
Percent identity: 53.11
Percent similarity: 62.46

Results for ZmAGO1E vs HvAGO1A :
Alignment length: 1237
Identical residues: 866
Similar residues: 58
Percent identity: 70.01
Percent similarity: 74.70

Results for ZmAGO1A vs ZmAGO1C :
Alignment length: 1208
Identical residues: 925
Similar residues: 34
Percent identity: 76.57
Percent similarity: 79.39

Results for ZmAGO1A vs ZmAGO1D :
Alignment length: 1241
Identical residues: 676
Similar residues: 100
Percent identity: 54.47
Percent similarity: 62.53

Results for ZmAGO1A vs ZmAGO2 :
Alignment length: 1203
Identical residues: 268
Similar residues: 140
Percent identity: 22.28
Percent similarity: 33.92

Results for ZmAGO1A vs ZmAGO7 :
Alignment length: 1202
Identical residues: 276
Similar residues: 144
Percent identity: 22.96
Percent similarity: 34.94

Results for ZmAGO1A vs ZmAGO5B :
Alignment length: 1203
Identical residues: 495

Similar residues: 144
Percent identity: 41.15
Percent similarity: 53.12

Results for ZmAGO1A vs ZmAGO5C :
Alignment length: 1194
Identical residues: 299
Similar residues: 148
Percent identity: 25.04
Percent similarity: 37.44

Results for ZmAGO1A vs ZmAGO10B :
Alignment length: 1154
Identical residues: 647
Similar residues: 88
Percent identity: 56.07
Percent similarity: 63.69

Results for ZmAGO1A vs ZmAGO18A :
Alignment length: 1188
Identical residues: 479
Similar residues: 129
Percent identity: 40.32
Percent similarity: 51.18

Results for ZmAGO1A vs ZmAGO4D :
Alignment length: 1199
Identical residues: 300
Similar residues: 160
Percent identity: 25.02
Percent similarity: 38.37

Results for ZmAGO1A vs ZmAGO5A :
Alignment length: 1187
Identical residues: 526
Similar residues: 149
Percent identity: 44.31
Percent similarity: 56.87

Results for ZmAGO1A vs ZmAGO18B :
Alignment length: 1235
Identical residues: 382
Similar residues: 146
Percent identity: 30.93
Percent similarity: 42.75

Results for ZmAGO1A vs OsAGO1A :
Alignment length: 1163
Identical residues: 867
Similar residues: 56
Percent identity: 74.55
Percent similarity: 79.36

Results for ZmAGO1A vs OsAGO1B :
Alignment length: 1169
Identical residues: 931
Similar residues: 40
Percent identity: 79.64
Percent similarity: 83.06

Results for ZmAGO1A vs OsAGO1C :
Alignment length: 1158
Identical residues: 716
Similar residues: 81
Percent identity: 61.83
Percent similarity: 68.83

Results for ZmAGO1A vs OsAGO1D :
Alignment length: 1163
Identical residues: 720
Similar residues: 93
Percent identity: 61.91
Percent similarity: 69.91

Results for ZmAGO1A vs OsAGO2 :
Alignment length: 1230
Identical residues: 294
Similar residues: 156
Percent identity: 23.90
Percent similarity: 36.59

Results for ZmAGO1A vs OsAGO3 :
Alignment length: 1282
Identical residues: 275
Similar residues: 151
Percent identity: 21.45
Percent similarity: 33.23

Results for ZmAGO1A vs OsAGO4A :
Alignment length: 1197
Identical residues: 306
Similar residues: 147
Percent identity: 25.56
Percent similarity: 37.84

Results for ZmAGO1A vs OsAGO4B :
Alignment length: 1200
Identical residues: 310
Similar residues: 151
Percent identity: 25.83
Percent similarity: 38.42

Results for ZmAGO1A vs OsAGO7 :
Alignment length: 1216
Identical residues: 321
Similar residues: 159
Percent identity: 26.40
Percent similarity: 39.47

Results for ZmAGO1A vs OsAGO11 :
Alignment length: 1176
Identical residues: 430
Similar residues: 126
Percent identity: 36.56
Percent similarity: 47.28

Results for ZmAGO1A vs OsAGO12 :
Alignment length: 1187
Identical residues: 516
Similar residues: 140
Percent identity: 43.47
Percent similarity: 55.27

Results for ZmAGO1A vs OsAGO13 :
Alignment length: 1524
Identical residues: 457
Similar residues: 111
Percent identity: 29.99
Percent similarity: 37.27

Results for ZmAGO1A vs OsAGO14 :
Alignment length: 1202
Identical residues: 510
Similar residues: 139
Percent identity: 42.43
Percent similarity: 53.99

Results for ZmAGO1A vs OsAGO15 :
Alignment length: 1202
Identical residues: 299
Similar residues: 136
Percent identity: 24.88
Percent similarity: 36.19

Results for ZmAGO1A vs OsAGO16 :
Alignment length: 1193
Identical residues: 295
Similar residues: 145
Percent identity: 24.73
Percent similarity: 36.88

Results for ZmAGO1A vs OsAGO17 :
Alignment length: 1154
Identical residues: 471
Similar residues: 111
Percent identity: 40.81
Percent similarity: 50.43

Results for ZmAGO1A vs OsAGO18 :
Alignment length: 1201
Identical residues: 462
Similar residues: 138
Percent identity: 38.47
Percent similarity: 49.96

Results for ZmAGO1A vs OsMEL1 :
Alignment length: 1183
Identical residues: 554
Similar residues: 132
Percent identity: 46.83
Percent similarity: 57.99

Results for ZmAGO1A vs OsPNH1 :
Alignment length: 1159
Identical residues: 654
Similar residues: 96
Percent identity: 56.43
Percent similarity: 64.71

Results for ZmAGO1A vs AtAGO1 :
Alignment length: 1165
Identical residues: 770
Similar residues: 68
Percent identity: 66.09
Percent similarity: 71.93

Results for ZmAGO1A vs AtAGO2 :
Alignment length: 1220
Identical residues: 295
Similar residues: 165
Percent identity: 24.18
Percent similarity: 37.70

Results for ZmAGO1A vs AtAGO3 :
Alignment length: 1301
Identical residues: 291
Similar residues: 180
Percent identity: 22.37

Percent similarity: 36.20

Results for ZmAGO1A vs AtAGO4 :
Alignment length: 1202
Identical residues: 308
Similar residues: 164
Percent identity: 25.62
Percent similarity: 39.27

Results for ZmAGO1A vs AtAGO5 :
Alignment length: 1180
Identical residues: 525
Similar residues: 126
Percent identity: 44.49
Percent similarity: 55.17

Results for ZmAGO1A vs AtAGO6 :
Alignment length: 1193
Identical residues: 305
Similar residues: 141
Percent identity: 25.57
Percent similarity: 37.38

Results for ZmAGO1A vs AtAGO7 :
Alignment length: 1219
Identical residues: 323
Similar residues: 136
Percent identity: 26.50
Percent similarity: 37.65

Results for ZmAGO1A vs AtAGO8 :
Alignment length: 1183
Identical residues: 283
Similar residues: 134
Percent identity: 23.92
Percent similarity: 35.25

Results for ZmAGO1A vs AtAGO9 :
Alignment length: 1193
Identical residues: 298
Similar residues: 164
Percent identity: 24.98
Percent similarity: 38.73

Results for ZmAGO1A vs AtAGO10 :
Alignment length: 1163
Identical residues: 646
Similar residues: 101
Percent identity: 55.55
Percent similarity: 64.23

Results for ZmAGO1A vs HvAGO4A :
Alignment length: 1204
Identical residues: 310
Similar residues: 144
Percent identity: 25.75
Percent similarity: 37.71

Results for ZmAGO1A vs HvAGO4B :
Alignment length: 1200
Identical residues: 276
Similar residues: 136
Percent identity: 23.00
Percent similarity: 34.33

Results for ZmAGO1A vs HvAGO7 :
Alignment length: 1216
Identical residues: 320
Similar residues: 146
Percent identity: 26.32
Percent similarity: 38.32

Results for ZmAGO1A vs HvAGO1D :
Alignment length: 1164
Identical residues: 688
Similar residues: 111
Percent identity: 59.11
Percent similarity: 68.64

Results for ZmAGO1A vs HvAGO5B :
Alignment length: 1163
Identical residues: 494
Similar residues: 112
Percent identity: 42.48
Percent similarity: 52.11

Results for ZmAGO1A vs HvAGO6 :
Alignment length: 1193
Identical residues: 298
Similar residues: 142
Percent identity: 24.98
Percent similarity: 36.88

Results for ZmAGO1A vs HvAGO2 :
Alignment length: 1228
Identical residues: 309
Similar residues: 152
Percent identity: 25.16
Percent similarity: 37.54

Results for ZmAGO1A vs HvAGO18 :
Alignment length: 1188
Identical residues: 481
Similar residues: 139
Percent identity: 40.49
Percent similarity: 52.19

Results for ZmAGO1A vs HvAGO5A :
Alignment length: 1182
Identical residues: 507
Similar residues: 153
Percent identity: 42.89
Percent similarity: 55.84

Results for ZmAGO1A vs HvAGO10 :
Alignment length: 1153
Identical residues: 596
Similar residues: 109
Percent identity: 51.69
Percent similarity: 61.14

Results for ZmAGO1A vs HvAGO1A :
Alignment length: 1281
Identical residues: 871
Similar residues: 57
Percent identity: 67.99
Percent similarity: 72.44

Results for ZmAGO1C vs ZmAGO1D :
Alignment length: 1220
Identical residues: 696
Similar residues: 90
Percent identity: 57.05
Percent similarity: 64.43

Results for ZmAGO1C vs ZmAGO2 :
Alignment length: 1174
Identical residues: 274
Similar residues: 141
Percent identity: 23.34
Percent similarity: 35.35

Results for ZmAGO1C vs ZmAGO7 :
Alignment length: 1173
Identical residues: 281
Similar residues: 146
Percent identity: 23.96
Percent similarity: 36.40

Results for ZmAGO1C vs ZmAGO5B :
Alignment length: 1179
Identical residues: 497
Similar residues: 139
Percent identity: 42.15
Percent similarity: 53.94

Results for ZmAGO1C vs ZmAGO5C :
Alignment length: 1174
Identical residues: 306
Similar residues: 137
Percent identity: 26.06
Percent similarity: 37.73

Results for ZmAGO1C vs ZmAGO10B :
Alignment length: 1134
Identical residues: 672
Similar residues: 88
Percent identity: 59.26
Percent similarity: 67.02

Results for ZmAGO1C vs ZmAGO18A :
Alignment length: 1196
Identical residues: 484
Similar residues: 120
Percent identity: 40.47
Percent similarity: 50.50

Results for ZmAGO1C vs ZmAGO4D :
Alignment length: 1178
Identical residues: 304
Similar residues: 155
Percent identity: 25.81
Percent similarity: 38.96

Results for ZmAGO1C vs ZmAGO5A :
Alignment length: 1164
Identical residues: 530
Similar residues: 155
Percent identity: 45.53
Percent similarity: 58.85

Results for ZmAGO1C vs ZmAGO18B :
Alignment length: 1211
Identical residues: 387
Similar residues: 138
Percent identity: 31.96
Percent similarity: 43.35

Results for ZmAGO1C vs OsAGO1A :
Alignment length: 1149
Identical residues: 924
Similar residues: 51
Percent identity: 80.42
Percent similarity: 84.86

Results for ZmAGO1C vs OsAGO1B :
Alignment length: 1168
Identical residues: 993
Similar residues: 22
Percent identity: 85.02
Percent similarity: 86.90

Results for ZmAGO1C vs OsAGO1C :
Alignment length: 1139
Identical residues: 739
Similar residues: 72
Percent identity: 64.88
Percent similarity: 71.20

Results for ZmAGO1C vs OsAGO1D :
Alignment length: 1139
Identical residues: 747
Similar residues: 84
Percent identity: 65.58
Percent similarity: 72.96

Results for ZmAGO1C vs OsAGO2 :
Alignment length: 1224
Identical residues: 300
Similar residues: 157
Percent identity: 24.51
Percent similarity: 37.34

Results for ZmAGO1C vs OsAGO3 :
Alignment length: 1274
Identical residues: 277
Similar residues: 156
Percent identity: 21.74
Percent similarity: 33.99

Results for ZmAGO1C vs OsAGO4A :
Alignment length: 1177
Identical residues: 304
Similar residues: 149
Percent identity: 25.83
Percent similarity: 38.49

Results for ZmAGO1C vs OsAGO4B :
Alignment length: 1179
Identical residues: 310
Similar residues: 147
Percent identity: 26.29
Percent similarity: 38.76

Results for ZmAGO1C vs OsAGO7 :
Alignment length: 1211
Identical residues: 325
Similar residues: 158
Percent identity: 26.84
Percent similarity: 39.88

Results for ZmAGO1C vs OsAGO11 :
Alignment length: 1153
Identical residues: 434
Similar residues: 125
Percent identity: 37.64
Percent similarity: 48.48

Results for ZmAGO1C vs OsAGO12 :
Alignment length: 1163
Identical residues: 517
Similar residues: 143
Percent identity: 44.45
Percent similarity: 56.75

Results for ZmAGO1C vs OsAGO13 :
Alignment length: 1505
Identical residues: 459
Similar residues: 111
Percent identity: 30.50
Percent similarity: 37.87

Results for ZmAGO1C vs OsAGO14 :
Alignment length: 1179
Identical residues: 517
Similar residues: 140
Percent identity: 43.85
Percent similarity: 55.73

Results for ZmAGO1C vs OsAGO15 :
Alignment length: 1181
Identical residues: 306
Similar residues: 128
Percent identity: 25.91
Percent similarity: 36.75

Results for ZmAGO1C vs OsAGO16 :
Alignment length: 1173
Identical residues: 298
Similar residues: 141
Percent identity: 25.40
Percent similarity: 37.43

Results for ZmAGO1C vs OsAGO17 :
Alignment length: 1131
Identical residues: 484
Similar residues: 107
Percent identity: 42.79
Percent similarity: 52.25

Results for ZmAGO1C vs OsAGO18 :
Alignment length: 1221
Identical residues: 461
Similar residues: 130
Percent identity: 37.76
Percent similarity: 48.40

Results for ZmAGO1C vs OsMEL1 :
Alignment length: 1160
Identical residues: 554
Similar residues: 129
Percent identity: 47.76
Percent similarity: 58.88

Results for ZmAGO1C vs OsPNH1 :
Alignment length: 1136
Identical residues: 675
Similar residues: 93
Percent identity: 59.42
Percent similarity: 67.61

Results for ZmAGO1C vs AtAGO1 :
Alignment length: 1137
Identical residues: 791
Similar residues: 62
Percent identity: 69.57
Percent similarity: 75.02

Results for ZmAGO1C vs AtAGO2 :
Alignment length: 1207
Identical residues: 295
Similar residues: 163
Percent identity: 24.44
Percent similarity: 37.95

Results for ZmAGO1C vs AtAGO3 :
Alignment length: 1315
Identical residues: 290
Similar residues: 178
Percent identity: 22.05
Percent similarity: 35.59

Results for ZmAGO1C vs AtAGO4 :
Alignment length: 1180
Identical residues: 311
Similar residues: 159
Percent identity: 26.36
Percent similarity: 39.83

Results for ZmAGO1C vs AtAGO5 :
Alignment length: 1155
Identical residues: 534
Similar residues: 131
Percent identity: 46.23
Percent similarity: 57.58

Results for ZmAGO1C vs AtAGO6 :
Alignment length: 1172
Identical residues: 306
Similar residues: 135
Percent identity: 26.11
Percent similarity: 37.63

Results for ZmAGO1C vs AtAGO7 :
Alignment length: 1194
Identical residues: 330
Similar residues: 138
Percent identity: 27.64
Percent similarity: 39.20

Results for ZmAGO1C vs AtAGO8 :
Alignment length: 1162
Identical residues: 291
Similar residues: 136
Percent identity: 25.04
Percent similarity: 36.75

Results for ZmAGO1C vs AtAGO9 :
Alignment length: 1172
Identical residues: 300
Similar residues: 162
Percent identity: 25.60
Percent similarity: 39.42

Results for ZmAGO1C vs AtAGO10 :
Alignment length: 1139

Identical residues: 667
Similar residues: 100
Percent identity: 58.56
Percent similarity: 67.34

Results for ZmAGO1C vs HvAGO4A :
Alignment length: 1180
Identical residues: 309
Similar residues: 147
Percent identity: 26.19
Percent similarity: 38.64

Results for ZmAGO1C vs HvAGO4B :
Alignment length: 1178
Identical residues: 284
Similar residues: 136
Percent identity: 24.11
Percent similarity: 35.65

Results for ZmAGO1C vs HvAGO7 :
Alignment length: 1193
Identical residues: 328
Similar residues: 142
Percent identity: 27.49
Percent similarity: 39.40

Results for ZmAGO1C vs HvAGO1D :
Alignment length: 1175
Identical residues: 709
Similar residues: 93
Percent identity: 60.34
Percent similarity: 68.26

Results for ZmAGO1C vs HvAGO5B :
Alignment length: 1143
Identical residues: 493
Similar residues: 114
Percent identity: 43.13
Percent similarity: 53.11

Results for ZmAGO1C vs HvAGO6 :
Alignment length: 1173
Identical residues: 302
Similar residues: 140
Percent identity: 25.75
Percent similarity: 37.68

Results for ZmAGO1C vs HvAGO2 :
Alignment length: 1220
Identical residues: 313
Similar residues: 155
Percent identity: 25.66
Percent similarity: 38.36

Results for ZmAGO1C vs HvAGO18 :
Alignment length: 1188
Identical residues: 491
Similar residues: 129
Percent identity: 41.33
Percent similarity: 52.19

Results for ZmAGO1C vs HvAGO5A :
Alignment length: 1158
Identical residues: 515
Similar residues: 163
Percent identity: 44.47
Percent similarity: 58.55

Results for ZmAGO1C vs HvAGO10 :
Alignment length: 1132
Identical residues: 615
Similar residues: 104
Percent identity: 54.33
Percent similarity: 63.52

Results for ZmAGO1C vs HvAGO1A :
Alignment length: 1273
Identical residues: 917
Similar residues: 42
Percent identity: 72.03
Percent similarity: 75.33

Results for ZmAGO1D vs ZmAGO2 :
Alignment length: 1142
Identical residues: 248
Similar residues: 143
Percent identity: 21.72
Percent similarity: 34.24

Results for ZmAGO1D vs ZmAGO7 :
Alignment length: 1142
Identical residues: 249
Similar residues: 149
Percent identity: 21.80
Percent similarity: 34.85

Results for ZmAGO1D vs ZmAGO5B :
Alignment length: 1176
Identical residues: 449
Similar residues: 139
Percent identity: 38.18
Percent similarity: 50.00

Results for ZmAGO1D vs ZmAGO5C :
Alignment length: 1139
Identical residues: 286
Similar residues: 130
Percent identity: 25.11
Percent similarity: 36.52

Results for ZmAGO1D vs ZmAGO10B :
Alignment length: 1099
Identical residues: 603
Similar residues: 102
Percent identity: 54.87
Percent similarity: 64.15

Results for ZmAGO1D vs ZmAGO18A :
Alignment length: 1199
Identical residues: 443
Similar residues: 125
Percent identity: 36.95
Percent similarity: 47.37

Results for ZmAGO1D vs ZmAGO4D :
Alignment length: 1147
Identical residues: 284
Similar residues: 139
Percent identity: 24.76
Percent similarity: 36.88

Results for ZmAGO1D vs ZmAGO5A :
Alignment length: 1160
Identical residues: 492
Similar residues: 148
Percent identity: 42.41
Percent similarity: 55.17

Results for ZmAGO1D vs ZmAGO18B :
Alignment length: 1199
Identical residues: 354
Similar residues: 156
Percent identity: 29.52
Percent similarity: 42.54

Results for ZmAGO1D vs OsAGO1A :
Alignment length: 1173
Identical residues: 680
Similar residues: 98
Percent identity: 57.97
Percent similarity: 66.33

Results for ZmAGO1D vs OsAGO1B :
Alignment length: 1209
Identical residues: 698
Similar residues: 87
Percent identity: 57.73
Percent similarity: 64.93

Results for ZmAGO1D vs OsAGO1C :
Alignment length: 1102
Identical residues: 744
Similar residues: 62
Percent identity: 67.51
Percent similarity: 73.14

Results for ZmAGO1D vs OsAGO1D :
Alignment length: 1125
Identical residues: 826
Similar residues: 51
Percent identity: 73.42
Percent similarity: 77.96

Results for ZmAGO1D vs OsAGO2 :
Alignment length: 1235
Identical residues: 265
Similar residues: 151
Percent identity: 21.46
Percent similarity: 33.68

Results for ZmAGO1D vs OsAGO3 :
Alignment length: 1288
Identical residues: 247
Similar residues: 141
Percent identity: 19.18
Percent similarity: 30.12

Results for ZmAGO1D vs OsAGO4A :
Alignment length: 1132
Identical residues: 285
Similar residues: 140
Percent identity: 25.18
Percent similarity: 37.54

Results for ZmAGO1D vs OsAGO4B :
Alignment length: 1137
Identical residues: 289
Similar residues: 140

Percent identity: 25.42
Percent similarity: 37.73

Results for ZmAGO1D vs OsAGO7 :
Alignment length: 1227
Identical residues: 297
Similar residues: 154
Percent identity: 24.21
Percent similarity: 36.76

Results for ZmAGO1D vs OsAGO11 :
Alignment length: 1137
Identical residues: 396
Similar residues: 136
Percent identity: 34.83
Percent similarity: 46.79

Results for ZmAGO1D vs OsAGO12 :
Alignment length: 1163
Identical residues: 490
Similar residues: 141
Percent identity: 42.13
Percent similarity: 54.26

Results for ZmAGO1D vs OsAGO13 :
Alignment length: 1438
Identical residues: 431
Similar residues: 124
Percent identity: 29.97
Percent similarity: 38.60

Results for ZmAGO1D vs OsAGO14 :
Alignment length: 1173
Identical residues: 473
Similar residues: 140
Percent identity: 40.32
Percent similarity: 52.26

Results for ZmAGO1D vs OsAGO15 :
Alignment length: 1134
Identical residues: 279
Similar residues: 133
Percent identity: 24.60
Percent similarity: 36.33

Results for ZmAGO1D vs OsAGO16 :
Alignment length: 1126
Identical residues: 282
Similar residues: 133
Percent identity: 25.04
Percent similarity: 36.86

Results for ZmAGO1D vs OsAGO17 :
Alignment length: 1091
Identical residues: 428
Similar residues: 125
Percent identity: 39.23
Percent similarity: 50.69

Results for ZmAGO1D vs OsAGO18 :
Alignment length: 1231
Identical residues: 424
Similar residues: 142
Percent identity: 34.44
Percent similarity: 45.98

Results for ZmAGO1D vs OsMEL1 :
Alignment length: 1160
Identical residues: 499
Similar residues: 136
Percent identity: 43.02
Percent similarity: 54.74

Results for ZmAGO1D vs OsPNH1 :
Alignment length: 1100
Identical residues: 611
Similar residues: 102
Percent identity: 55.55
Percent similarity: 64.82

Results for ZmAGO1D vs AtAGO1 :
Alignment length: 1147
Identical residues: 646
Similar residues: 96
Percent identity: 56.32
Percent similarity: 64.69

Results for ZmAGO1D vs AtAGO2 :
Alignment length: 1210
Identical residues: 267
Similar residues: 159
Percent identity: 22.07
Percent similarity: 35.21

Results for ZmAGO1D vs AtAGO3 :
Alignment length: 1343
Identical residues: 260
Similar residues: 171
Percent identity: 19.36
Percent similarity: 32.09

Results for ZmAGO1D vs AtAGO4 :
Alignment length: 1139
Identical residues: 281
Similar residues: 156
Percent identity: 24.67
Percent similarity: 38.37

Results for ZmAGO1D vs AtAGO5 :
Alignment length: 1135
Identical residues: 479
Similar residues: 145
Percent identity: 42.20
Percent similarity: 54.98

Results for ZmAGO1D vs AtAGO6 :
Alignment length: 1127
Identical residues: 283
Similar residues: 139
Percent identity: 25.11
Percent similarity: 37.44

Results for ZmAGO1D vs AtAGO7 :
Alignment length: 1206
Identical residues: 281
Similar residues: 144
Percent identity: 23.30
Percent similarity: 35.24

Results for ZmAGO1D vs AtAGO8 :
Alignment length: 1115
Identical residues: 265
Similar residues: 131
Percent identity: 23.77
Percent similarity: 35.52

Results for ZmAGO1D vs AtAGO9 :
Alignment length: 1128
Identical residues: 284
Similar residues: 146
Percent identity: 25.18
Percent similarity: 38.12

Results for ZmAGO1D vs AtAGO10 :
Alignment length: 1103
Identical residues: 600
Similar residues: 110
Percent identity: 54.40
Percent similarity: 64.37

Results for ZmAGO1D vs HvAGO4A :
Alignment length: 1147
Identical residues: 286
Similar residues: 140
Percent identity: 24.93
Percent similarity: 37.14

Results for ZmAGO1D vs HvAGO4B :
Alignment length: 1132
Identical residues: 262
Similar residues: 134
Percent identity: 23.14
Percent similarity: 34.98

Results for ZmAGO1D vs HvAGO7 :
Alignment length: 1199
Identical residues: 288
Similar residues: 148
Percent identity: 24.02
Percent similarity: 36.36

Results for ZmAGO1D vs HvAGO1D :
Alignment length: 1157
Identical residues: 769
Similar residues: 73
Percent identity: 66.46
Percent similarity: 72.77

Results for ZmAGO1D vs HvAGO5B :
Alignment length: 1091
Identical residues: 440
Similar residues: 133
Percent identity: 40.33
Percent similarity: 52.52

Results for ZmAGO1D vs HvAGO6 :
Alignment length: 1126
Identical residues: 283
Similar residues: 134
Percent identity: 25.13
Percent similarity: 37.03

Results for ZmAGO1D vs HvAGO2 :
Alignment length: 1230
Identical residues: 276
Similar residues: 144
Percent identity: 22.44
Percent similarity: 34.15

Results for ZmAGO1D vs HvAGO18 :

Alignment length: 1193
Identical residues: 454
Similar residues: 130
Percent identity: 38.06
Percent similarity: 48.95

Results for ZmAGO1D vs HvAGO5A :

Alignment length: 1157
Identical residues: 479
Similar residues: 144
Percent identity: 41.40
Percent similarity: 53.85

Results for ZmAGO1D vs HvAGO10 :

Alignment length: 1096
Identical residues: 566
Similar residues: 105
Percent identity: 51.64
Percent similarity: 61.22

Results for ZmAGO1D vs HvAGO1A :

Alignment length: 1309
Identical residues: 639
Similar residues: 103
Percent identity: 48.82
Percent similarity: 56.68

Results for ZmAGO2 vs ZmAGO7 :

Alignment length: 884
Identical residues: 767
Similar residues: 28
Percent identity: 86.76
Percent similarity: 89.93

Results for ZmAGO2 vs ZmAGO5B :

Alignment length: 1101
Identical residues: 266
Similar residues: 121
Percent identity: 24.16
Percent similarity: 35.15

Results for ZmAGO2 vs ZmAGO5C :

Alignment length: 1012
Identical residues: 249
Similar residues: 121
Percent identity: 24.60
Percent similarity: 36.56

Results for ZmAGO2 vs ZmAGO10B :

Alignment length: 1031
Identical residues: 261
Similar residues: 136
Percent identity: 25.32
Percent similarity: 38.51

Results for ZmAGO2 vs ZmAGO18A :

Alignment length: 1115
Identical residues: 259
Similar residues: 154
Percent identity: 23.23
Percent similarity: 37.04

Results for ZmAGO2 vs ZmAGO4D :

Alignment length: 1006
Identical residues: 241
Similar residues: 132
Percent identity: 23.96
Percent similarity: 37.08

Results for ZmAGO2 vs ZmAGO5A :

Alignment length: 1109
Identical residues: 273
Similar residues: 130
Percent identity: 24.62
Percent similarity: 36.34

Results for ZmAGO2 vs ZmAGO18B :

Alignment length: 1042
Identical residues: 216
Similar residues: 165
Percent identity: 20.73
Percent similarity: 36.56

Results for ZmAGO2 vs OsAGO1A :

Alignment length: 1133
Identical residues: 268
Similar residues: 143
Percent identity: 23.65
Percent similarity: 36.28

Results for ZmAGO2 vs OsAGO1B :

Alignment length: 1169
Identical residues: 273
Similar residues: 142
Percent identity: 23.35
Percent similarity: 35.50

Results for ZmAGO2 vs OsAGO1C :

Alignment length: 1078
Identical residues: 267
Similar residues: 136
Percent identity: 24.77
Percent similarity: 37.38

Results for ZmAGO2 vs OsAGO1D :

Alignment length: 1096
Identical residues: 268
Similar residues: 139
Percent identity: 24.45
Percent similarity: 37.14

Results for ZmAGO2 vs OsAGO2 :

Alignment length: 1041
Identical residues: 635
Similar residues: 75
Percent identity: 61.00
Percent similarity: 68.20

Results for ZmAGO2 vs OsAGO3 :

Alignment length: 1128
Identical residues: 520
Similar residues: 94
Percent identity: 46.10
Percent similarity: 54.43

Results for ZmAGO2 vs OsAGO4A :

Alignment length: 991
Identical residues: 249
Similar residues: 135
Percent identity: 25.13
Percent similarity: 38.75

Results for ZmAGO2 vs OsAGO4B :

Alignment length: 992
Identical residues: 244
Similar residues: 136
Percent identity: 24.60
Percent similarity: 38.31

Results for ZmAGO2 vs OsAGO7 :

Alignment length: 1083
Identical residues: 309
Similar residues: 136
Percent identity: 28.53
Percent similarity: 41.09

Results for ZmAGO2 vs OsAGO11 :

Alignment length: 1034
Identical residues: 228
Similar residues: 115
Percent identity: 22.05
Percent similarity: 33.17

Results for ZmAGO2 vs OsAGO12 :

Alignment length: 1108
Identical residues: 269
Similar residues: 133
Percent identity: 24.28
Percent similarity: 36.28

Results for ZmAGO2 vs OsAGO13 :

Alignment length: 1326
Identical residues: 218
Similar residues: 133
Percent identity: 16.44
Percent similarity: 26.47

Results for ZmAGO2 vs OsAGO14 :

Alignment length: 1119
Identical residues: 265
Similar residues: 131
Percent identity: 23.68
Percent similarity: 35.39

Results for ZmAGO2 vs OsAGO15 :

Alignment length: 980
Identical residues: 234
Similar residues: 136
Percent identity: 23.88
Percent similarity: 37.76

Results for ZmAGO2 vs OsAGO16 :

Alignment length: 972
Identical residues: 246
Similar residues: 123
Percent identity: 25.31
Percent similarity: 37.96

Results for ZmAGO2 vs OsAGO17 :

Alignment length: 962
Identical residues: 218
Similar residues: 128
Percent identity: 22.66
Percent similarity: 35.97

Results for ZmAGO2 vs OsAGO18 :

Alignment length: 1150
Identical residues: 256
Similar residues: 149
Percent identity: 22.26
Percent similarity: 35.22

Results for ZmAGO2 vs OsMEL1 :
Alignment length: 1110
Identical residues: 269
Similar residues: 141
Percent identity: 24.23
Percent similarity: 36.94

Results for ZmAGO2 vs OsPNH1 :
Alignment length: 1044
Identical residues: 265
Similar residues: 137
Percent identity: 25.38
Percent similarity: 38.51

Results for ZmAGO2 vs AtAGO1 :
Alignment length: 1101
Identical residues: 277
Similar residues: 142
Percent identity: 25.16
Percent similarity: 38.06

Results for ZmAGO2 vs AtAGO2 :
Alignment length: 1038
Identical residues: 339
Similar residues: 152
Percent identity: 32.66
Percent similarity: 47.30

Results for ZmAGO2 vs AtAGO3 :
Alignment length: 1212
Identical residues: 342
Similar residues: 154
Percent identity: 28.22
Percent similarity: 40.92

Results for ZmAGO2 vs AtAGO4 :
Alignment length: 1007
Identical residues: 241
Similar residues: 132
Percent identity: 23.93
Percent similarity: 37.04

Results for ZmAGO2 vs AtAGO5 :
Alignment length: 1065
Identical residues: 266
Similar residues: 140
Percent identity: 24.98
Percent similarity: 38.12

Results for ZmAGO2 vs AtAGO6 :
Alignment length: 967
Identical residues: 246
Similar residues: 132
Percent identity: 25.44
Percent similarity: 39.09

Results for ZmAGO2 vs AtAGO7 :
Alignment length: 1044
Identical residues: 292
Similar residues: 143
Percent identity: 27.97
Percent similarity: 41.67

Results for ZmAGO2 vs AtAGO8 :
Alignment length: 959
Identical residues: 224
Similar residues: 124
Percent identity: 23.36
Percent similarity: 36.29

Results for ZmAGO2 vs AtAGO9 :
Alignment length: 983
Identical residues: 243
Similar residues: 137
Percent identity: 24.72
Percent similarity: 38.66

Results for ZmAGO2 vs AtAGO10 :
Alignment length: 1053
Identical residues: 263
Similar residues: 131
Percent identity: 24.98
Percent similarity: 37.42

Results for ZmAGO2 vs HvAGO4A :
Alignment length: 991
Identical residues: 248
Similar residues: 139
Percent identity: 25.03
Percent similarity: 39.05

Results for ZmAGO2 vs HvAGO4B :
Alignment length: 958
Identical residues: 219
Similar residues: 125
Percent identity: 22.86
Percent similarity: 35.91

Results for ZmAGO2 vs HvAGO7 :
Alignment length: 1045
Identical residues: 304
Similar residues: 134
Percent identity: 29.09
Percent similarity: 41.91

Results for ZmAGO2 vs HvAGO1D :
Alignment length: 1117
Identical residues: 264
Similar residues: 147
Percent identity: 23.63
Percent similarity: 36.79

Results for ZmAGO2 vs HvAGO5B :
Alignment length: 936
Identical residues: 276
Similar residues: 129
Percent identity: 29.49
Percent similarity: 43.27

Results for ZmAGO2 vs HvAGO6 :
Alignment length: 972
Identical residues: 245
Similar residues: 128
Percent identity: 25.21
Percent similarity: 38.37

Results for ZmAGO2 vs HvAGO2 :
Alignment length: 1051
Identical residues: 614
Similar residues: 87
Percent identity: 58.42
Percent similarity: 66.70

Results for ZmAGO2 vs HvAGO18 :
Alignment length: 1126
Identical residues: 250
Similar residues: 154
Percent identity: 22.20
Percent similarity: 35.88

Results for ZmAGO2 vs HvAGO5A :
Alignment length: 1087
Identical residues: 271
Similar residues: 122
Percent identity: 24.93
Percent similarity: 36.15

Results for ZmAGO2 vs HvAGO10 :
Alignment length: 1017
Identical residues: 259
Similar residues: 132
Percent identity: 25.47
Percent similarity: 38.45

Results for ZmAGO2 vs HvAGO1A :
Alignment length: 1268
Identical residues: 249
Similar residues: 141
Percent identity: 19.64
Percent similarity: 30.76

Results for ZmAGO7 vs ZmAGO5B :
Alignment length: 1100
Identical residues: 267
Similar residues: 129
Percent identity: 24.27
Percent similarity: 36.00

Results for ZmAGO7 vs ZmAGO5C :
Alignment length: 1016
Identical residues: 260
Similar residues: 120
Percent identity: 25.59
Percent similarity: 37.40

Results for ZmAGO7 vs ZmAGO10B :
Alignment length: 1034
Identical residues: 270
Similar residues: 137
Percent identity: 26.11
Percent similarity: 39.36

Results for ZmAGO7 vs ZmAGO18A :
Alignment length: 1115
Identical residues: 268
Similar residues: 147
Percent identity: 24.04
Percent similarity: 37.22

Results for ZmAGO7 vs ZmAGO4D :
Alignment length: 1010
Identical residues: 251

Similar residues: 129
Percent identity: 24.85
Percent similarity: 37.62

Results for ZmAGO7 vs ZmAGO5A :
Alignment length: 1109
Identical residues: 276
Similar residues: 131
Percent identity: 24.89
Percent similarity: 36.70

Results for ZmAGO7 vs ZmAGO18B :
Alignment length: 1042
Identical residues: 217
Similar residues: 160
Percent identity: 20.83
Percent similarity: 36.18

Results for ZmAGO7 vs OsAGO1A :
Alignment length: 1132
Identical residues: 275
Similar residues: 145
Percent identity: 24.29
Percent similarity: 37.10

Results for ZmAGO7 vs OsAGO1B :
Alignment length: 1168
Identical residues: 279
Similar residues: 147
Percent identity: 23.89
Percent similarity: 36.47

Results for ZmAGO7 vs OsAGO1C :
Alignment length: 1077
Identical residues: 270
Similar residues: 138
Percent identity: 25.07
Percent similarity: 37.88

Results for ZmAGO7 vs OsAGO1D :
Alignment length: 1095
Identical residues: 270
Similar residues: 143
Percent identity: 24.66
Percent similarity: 37.72

Results for ZmAGO7 vs OsAGO2 :
Alignment length: 1039
Identical residues: 660
Similar residues: 70
Percent identity: 63.52
Percent similarity: 70.26

Results for ZmAGO7 vs OsAGO3 :
Alignment length: 1125
Identical residues: 535
Similar residues: 81
Percent identity: 47.56
Percent similarity: 54.76

Results for ZmAGO7 vs OsAGO4A :
Alignment length: 996
Identical residues: 255
Similar residues: 131
Percent identity: 25.60
Percent similarity: 38.76

Results for ZmAGO7 vs OsAGO4B :
Alignment length: 996
Identical residues: 253
Similar residues: 131
Percent identity: 25.40
Percent similarity: 38.55

Results for ZmAGO7 vs OsAGO7 :
Alignment length: 1086
Identical residues: 298
Similar residues: 144
Percent identity: 27.44
Percent similarity: 40.70

Results for ZmAGO7 vs OsAGO11 :
Alignment length: 1039
Identical residues: 237
Similar residues: 113
Percent identity: 22.81
Percent similarity: 33.69

Results for ZmAGO7 vs OsAGO12 :
Alignment length: 1111
Identical residues: 267
Similar residues: 138
Percent identity: 24.03
Percent similarity: 36.45

Results for ZmAGO7 vs OsAGO13 :
Alignment length: 1333
Identical residues: 216
Similar residues: 136
Percent identity: 16.20
Percent similarity: 26.41

Results for ZmAGO7 vs OsAGO14 :
Alignment length: 1118
Identical residues: 270
Similar residues: 135
Percent identity: 24.15
Percent similarity: 36.23

Results for ZmAGO7 vs OsAGO15 :
Alignment length: 988
Identical residues: 239
Similar residues: 137
Percent identity: 24.19
Percent similarity: 38.06

Results for ZmAGO7 vs OsAGO16 :
Alignment length: 976
Identical residues: 257
Similar residues: 118
Percent identity: 26.33
Percent similarity: 38.42

Results for ZmAGO7 vs OsAGO17 :
Alignment length: 980
Identical residues: 220
Similar residues: 132
Percent identity: 22.45
Percent similarity: 35.92

Results for ZmAGO7 vs OsAGO18 :
Alignment length: 1150
Identical residues: 258
Similar residues: 152
Percent identity: 22.43
Percent similarity: 35.65

Results for ZmAGO7 vs OsMEL1 :
Alignment length: 1109
Identical residues: 271
Similar residues: 145
Percent identity: 24.44
Percent similarity: 37.51

Results for ZmAGO7 vs OsPNH1 :
Alignment length: 1046
Identical residues: 273
Similar residues: 133
Percent identity: 26.10
Percent similarity: 38.81

Results for ZmAGO7 vs AtAGO1 :
Alignment length: 1103
Identical residues: 280
Similar residues: 145
Percent identity: 25.39
Percent similarity: 38.53

Results for ZmAGO7 vs AtAGO2 :
Alignment length: 1037
Identical residues: 353
Similar residues: 148
Percent identity: 34.04
Percent similarity: 48.31

Results for ZmAGO7 vs AtAGO3 :
Alignment length: 1211
Identical residues: 355
Similar residues: 152
Percent identity: 29.31
Percent similarity: 41.87

Results for ZmAGO7 vs AtAGO4 :
Alignment length: 1010
Identical residues: 249
Similar residues: 138
Percent identity: 24.65
Percent similarity: 38.32

Results for ZmAGO7 vs AtAGO5 :
Alignment length: 1064
Identical residues: 267
Similar residues: 143
Percent identity: 25.09
Percent similarity: 38.53

Results for ZmAGO7 vs AtAGO6 :
Alignment length: 975
Identical residues: 247
Similar residues: 132
Percent identity: 25.33
Percent similarity: 38.87

Results for ZmAGO7 vs AtAGO7 :
Alignment length: 1043
Identical residues: 296
Similar residues: 143
Percent identity: 28.38

Percent similarity: 42.09

Results for ZmAGO7 vs AtAGO8 :
Alignment length: 965
Identical residues: 232
Similar residues: 128
Percent identity: 24.04
Percent similarity: 37.31

Results for ZmAGO7 vs AtAGO9 :
Alignment length: 983
Identical residues: 249
Similar residues: 135
Percent identity: 25.33
Percent similarity: 39.06

Results for ZmAGO7 vs AtAGO10 :
Alignment length: 1052
Identical residues: 268
Similar residues: 129
Percent identity: 25.48
Percent similarity: 37.74

Results for ZmAGO7 vs HvAGO4A :
Alignment length: 995
Identical residues: 250
Similar residues: 136
Percent identity: 25.13
Percent similarity: 38.79

Results for ZmAGO7 vs HvAGO4B :
Alignment length: 976
Identical residues: 221
Similar residues: 126
Percent identity: 22.64
Percent similarity: 35.55

Results for ZmAGO7 vs HvAGO7 :
Alignment length: 1044
Identical residues: 296
Similar residues: 139
Percent identity: 28.35
Percent similarity: 41.67

Results for ZmAGO7 vs HvAGO1D :
Alignment length: 1116
Identical residues: 268
Similar residues: 151
Percent identity: 24.01
Percent similarity: 37.54

Results for ZmAGO7 vs HvAGO5B :
Alignment length: 954
Identical residues: 273
Similar residues: 132
Percent identity: 28.62
Percent similarity: 42.45

Results for ZmAGO7 vs HvAGO6 :
Alignment length: 976
Identical residues: 256
Similar residues: 127
Percent identity: 26.23
Percent similarity: 39.24

Results for ZmAGO7 vs HvAGO2 :
Alignment length: 1048
Identical residues: 634
Similar residues: 80
Percent identity: 60.50
Percent similarity: 68.13

Results for ZmAGO7 vs HvAGO18 :
Alignment length: 1126
Identical residues: 256
Similar residues: 160
Percent identity: 22.74
Percent similarity: 36.94

Results for ZmAGO7 vs HvAGO5A :
Alignment length: 1094
Identical residues: 274
Similar residues: 125
Percent identity: 25.05
Percent similarity: 36.47

Results for ZmAGO7 vs HvAGO10 :
Alignment length: 1021
Identical residues: 262
Similar residues: 134
Percent identity: 25.66
Percent similarity: 38.79

Results for ZmAGO7 vs HvAGO1A :
Alignment length: 1267
Identical residues: 256
Similar residues: 144
Percent identity: 20.21
Percent similarity: 31.57

Results for ZmAGO5B vs ZmAGO5C :
Alignment length: 1115
Identical residues: 277
Similar residues: 151
Percent identity: 24.84
Percent similarity: 38.39

Results for ZmAGO5B vs ZmAGO10B :
Alignment length: 1089
Identical residues: 462
Similar residues: 134
Percent identity: 42.42
Percent similarity: 54.73

Results for ZmAGO5B vs ZmAGO18A :
Alignment length: 1123
Identical residues: 400
Similar residues: 154
Percent identity: 35.62
Percent similarity: 49.33

Results for ZmAGO5B vs ZmAGO4D :
Alignment length: 1119
Identical residues: 284
Similar residues: 158
Percent identity: 25.38
Percent similarity: 39.50

Results for ZmAGO5B vs ZmAGO5A :
Alignment length: 1090
Identical residues: 512
Similar residues: 139
Percent identity: 46.97
Percent similarity: 59.72

Results for ZmAGO5B vs ZmAGO18B :
Alignment length: 1135
Identical residues: 329
Similar residues: 157
Percent identity: 28.99
Percent similarity: 42.82

Results for ZmAGO5B vs OsAGO1A :
Alignment length: 1144
Identical residues: 492
Similar residues: 144
Percent identity: 43.01
Percent similarity: 55.59

Results for ZmAGO5B vs OsAGO1B :
Alignment length: 1169
Identical residues: 493
Similar residues: 139
Percent identity: 42.17
Percent similarity: 54.06

Results for ZmAGO5B vs OsAGO1C :
Alignment length: 1107
Identical residues: 476
Similar residues: 123
Percent identity: 43.00
Percent similarity: 54.11

Results for ZmAGO5B vs OsAGO1D :
Alignment length: 1108
Identical residues: 473
Similar residues: 136
Percent identity: 42.69
Percent similarity: 54.96

Results for ZmAGO5B vs OsAGO2 :
Alignment length: 1151
Identical residues: 301
Similar residues: 139
Percent identity: 26.15
Percent similarity: 38.23

Results for ZmAGO5B vs OsAGO3 :
Alignment length: 1201
Identical residues: 283
Similar residues: 131
Percent identity: 23.56
Percent similarity: 34.47

Results for ZmAGO5B vs OsAGO4A :
Alignment length: 1118
Identical residues: 280
Similar residues: 161
Percent identity: 25.04
Percent similarity: 39.45

Results for ZmAGO5B vs OsAGO4B :
Alignment length: 1119
Identical residues: 295
Similar residues: 154
Percent identity: 26.36
Percent similarity: 40.13

Results for ZmAGO5B vs OsAGO7 :

Alignment length: 1146

Identical residues: 323

Similar residues: 157

Percent identity: 28.18

Percent similarity: 41.88

Results for ZmAGO5B vs OsAGO11 :

Alignment length: 1079

Identical residues: 417

Similar residues: 124

Percent identity: 38.65

Percent similarity: 50.14

Results for ZmAGO5B vs OsAGO12 :

Alignment length: 1089

Identical residues: 532

Similar residues: 118

Percent identity: 48.85

Percent similarity: 59.69

Results for ZmAGO5B vs OsAGO13 :

Alignment length: 1445

Identical residues: 471

Similar residues: 103

Percent identity: 32.60

Percent similarity: 39.72

Results for ZmAGO5B vs OsAGO14 :

Alignment length: 1083

Identical residues: 615

Similar residues: 118

Percent identity: 56.79

Percent similarity: 67.68

Results for ZmAGO5B vs OsAGO15 :

Alignment length: 1122

Identical residues: 266

Similar residues: 146

Percent identity: 23.71

Percent similarity: 36.72

Results for ZmAGO5B vs OsAGO16 :

Alignment length: 1116

Identical residues: 268

Similar residues: 149

Percent identity: 24.01

Percent similarity: 37.37

Results for ZmAGO5B vs OsAGO17 :

Alignment length: 1085

Identical residues: 343

Similar residues: 132

Percent identity: 31.61

Percent similarity: 43.78

Results for ZmAGO5B vs OsAGO18 :

Alignment length: 1147

Identical residues: 404

Similar residues: 143

Percent identity: 35.22

Percent similarity: 47.69

Results for ZmAGO5B vs OsMEL1 :

Alignment length: 1082

Identical residues: 606

Similar residues: 125

Percent identity: 56.01

Percent similarity: 67.56

Results for ZmAGO5B vs OsPNH1 :

Alignment length: 1088

Identical residues: 464

Similar residues: 140

Percent identity: 42.65

Percent similarity: 55.51

Results for ZmAGO5B vs AtAGO1 :

Alignment length: 1105

Identical residues: 494

Similar residues: 129

Percent identity: 44.71

Percent similarity: 56.38

Results for ZmAGO5B vs AtAGO2 :

Alignment length: 1136

Identical residues: 279

Similar residues: 160

Percent identity: 24.56

Percent similarity: 38.64

Results for ZmAGO5B vs AtAGO3 :

Alignment length: 1269

Identical residues: 277

Similar residues: 162

Percent identity: 21.83

Percent similarity: 34.59

Results for ZmAGO5B vs AtAGO4 :

Alignment length: 1121

Identical residues: 289

Similar residues: 155

Percent identity: 25.78

Percent similarity: 39.61

Results for ZmAGO5B vs AtAGO5 :

Alignment length: 1071

Identical residues: 512

Similar residues: 136

Percent identity: 47.81

Percent similarity: 60.50

Results for ZmAGO5B vs AtAGO6 :

Alignment length: 1112

Identical residues: 275

Similar residues: 140

Percent identity: 24.73

Percent similarity: 37.32

Results for ZmAGO5B vs AtAGO7 :

Alignment length: 1119

Identical residues: 300

Similar residues: 135

Percent identity: 26.81

Percent similarity: 38.87

Results for ZmAGO5B vs AtAGO8 :

Alignment length: 1103

Identical residues: 259

Similar residues: 139

Percent identity: 23.48

Percent similarity: 36.08

Results for ZmAGO5B vs AtAGO9 :

Alignment length: 1113

Identical residues: 287

Similar residues: 163

Percent identity: 25.79

Percent similarity: 40.43

Results for ZmAGO5B vs AtAGO10 :

Alignment length: 1096

Identical residues: 456

Similar residues: 129

Percent identity: 41.61

Percent similarity: 53.38

Results for ZmAGO5B vs HvAGO4A :

Alignment length: 1119

Identical residues: 286

Similar residues: 158

Percent identity: 25.56

Percent similarity: 39.68

Results for ZmAGO5B vs HvAGO4B :

Alignment length: 1121

Identical residues: 259

Similar residues: 128

Percent identity: 23.10

Percent similarity: 34.52

Results for ZmAGO5B vs HvAGO7 :

Alignment length: 1118

Identical residues: 315

Similar residues: 147

Percent identity: 28.18

Percent similarity: 41.32

Results for ZmAGO5B vs HvAGO1D :

Alignment length: 1142

Identical residues: 470

Similar residues: 139

Percent identity: 41.16

Percent similarity: 53.33

Results for ZmAGO5B vs HvAGO5B :

Alignment length: 1055

Identical residues: 569

Similar residues: 94

Percent identity: 53.93

Percent similarity: 62.84

Results for ZmAGO5B vs HvAGO6 :

Alignment length: 1116

Identical residues: 279

Similar residues: 140

Percent identity: 25.00

Percent similarity: 37.54

Results for ZmAGO5B vs HvAGO2 :

Alignment length: 1147

Identical residues: 304

Similar residues: 143

Percent identity: 26.50

Percent similarity: 38.97

Results for ZmAGO5B vs HvAGO18 :

Alignment length: 1118

Identical residues: 419
Similar residues: 145
Percent identity: 37.48
Percent similarity: 50.45

Results for ZmAGO5B vs HvAGO5A :
Alignment length: 1087
Identical residues: 492
Similar residues: 153
Percent identity: 45.26
Percent similarity: 59.34

Results for ZmAGO5B vs HvAGO10 :
Alignment length: 1087
Identical residues: 438
Similar residues: 144
Percent identity: 40.29
Percent similarity: 53.54

Results for ZmAGO5B vs HvAGO1A :
Alignment length: 1264
Identical residues: 464
Similar residues: 148
Percent identity: 36.71
Percent similarity: 48.42

Results for ZmAGO5C vs ZmAGO10B :
Alignment length: 1040
Identical residues: 301
Similar residues: 141
Percent identity: 28.94
Percent similarity: 42.50

Results for ZmAGO5C vs ZmAGO18A :
Alignment length: 1142
Identical residues: 283
Similar residues: 150
Percent identity: 24.78
Percent similarity: 37.92

Results for ZmAGO5C vs ZmAGO4D :
Alignment length: 945
Identical residues: 497
Similar residues: 111
Percent identity: 52.59
Percent similarity: 64.34

Results for ZmAGO5C vs ZmAGO5A :
Alignment length: 1111
Identical residues: 300
Similar residues: 132
Percent identity: 27.00
Percent similarity: 38.88

Results for ZmAGO5C vs ZmAGO18B :
Alignment length: 1079
Identical residues: 249
Similar residues: 152
Percent identity: 23.08
Percent similarity: 37.16

Results for ZmAGO5C vs OsAGO1A :
Alignment length: 1135
Identical residues: 308
Similar residues: 138
Percent identity: 27.14
Percent similarity: 39.30

Results for ZmAGO5C vs OsAGO1B :
Alignment length: 1167
Identical residues: 303
Similar residues: 138
Percent identity: 25.96
Percent similarity: 37.79

Results for ZmAGO5C vs OsAGO1C :
Alignment length: 1076
Identical residues: 303
Similar residues: 131
Percent identity: 28.16
Percent similarity: 40.33

Results for ZmAGO5C vs OsAGO1D :
Alignment length: 1088
Identical residues: 307
Similar residues: 128
Percent identity: 28.22
Percent similarity: 39.98

Results for ZmAGO5C vs OsAGO2 :
Alignment length: 1135
Identical residues: 250
Similar residues: 125
Percent identity: 22.03
Percent similarity: 33.04

Results for ZmAGO5C vs OsAGO3 :
Alignment length: 1195
Identical residues: 245
Similar residues: 134
Percent identity: 20.50
Percent similarity: 31.72

Results for ZmAGO5C vs OsAGO4A :
Alignment length: 940
Identical residues: 518
Similar residues: 111
Percent identity: 55.11
Percent similarity: 66.91

Results for ZmAGO5C vs OsAGO4B :
Alignment length: 944
Identical residues: 521
Similar residues: 102
Percent identity: 55.19
Percent similarity: 66.00

Results for ZmAGO5C vs OsAGO7 :
Alignment length: 1118
Identical residues: 252
Similar residues: 157
Percent identity: 22.54
Percent similarity: 36.58

Results for ZmAGO5C vs OsAGO11 :
Alignment length: 1055
Identical residues: 257
Similar residues: 106
Percent identity: 24.36
Percent similarity: 34.41

Results for ZmAGO5C vs OsAGO12 :
Alignment length: 1110
Identical residues: 300
Similar residues: 137
Percent identity: 27.03
Percent similarity: 39.37

Results for ZmAGO5C vs OsAGO13 :
Alignment length: 1357
Identical residues: 257
Similar residues: 132
Percent identity: 18.94
Percent similarity: 28.67

Results for ZmAGO5C vs OsAGO14 :
Alignment length: 1127
Identical residues: 289
Similar residues: 130
Percent identity: 25.64
Percent similarity: 37.18

Results for ZmAGO5C vs OsAGO15 :
Alignment length: 946
Identical residues: 458
Similar residues: 124
Percent identity: 48.41
Percent similarity: 61.52

Results for ZmAGO5C vs OsAGO16 :
Alignment length: 923
Identical residues: 773
Similar residues: 47
Percent identity: 83.75
Percent similarity: 88.84

Results for ZmAGO5C vs OsAGO17 :
Alignment length: 985
Identical residues: 256
Similar residues: 140
Percent identity: 25.99
Percent similarity: 40.20

Results for ZmAGO5C vs OsAGO18 :
Alignment length: 1170
Identical residues: 272
Similar residues: 144
Percent identity: 23.25
Percent similarity: 35.56

Results for ZmAGO5C vs OsMEL1 :
Alignment length: 1115
Identical residues: 297
Similar residues: 145
Percent identity: 26.64
Percent similarity: 39.64

Results for ZmAGO5C vs OsPNH1 :
Alignment length: 1048
Identical residues: 300
Similar residues: 141
Percent identity: 28.63
Percent similarity: 42.08

Results for ZmAGO5C vs AtAGO1 :
Alignment length: 1106
Identical residues: 302
Similar residues: 141

Percent identity: 27.31
Percent similarity: 40.05

Results for ZmAGO5C vs AtAGO2 :
Alignment length: 1097
Identical residues: 241
Similar residues: 135
Percent identity: 21.97
Percent similarity: 34.28

Results for ZmAGO5C vs AtAGO3 :
Alignment length: 1268
Identical residues: 249
Similar residues: 141
Percent identity: 19.64
Percent similarity: 30.76

Results for ZmAGO5C vs AtAGO4 :
Alignment length: 941
Identical residues: 506
Similar residues: 128
Percent identity: 53.77
Percent similarity: 67.38

Results for ZmAGO5C vs AtAGO5 :
Alignment length: 1081
Identical residues: 304
Similar residues: 116
Percent identity: 28.12
Percent similarity: 38.85

Results for ZmAGO5C vs AtAGO6 :
Alignment length: 928
Identical residues: 529
Similar residues: 100
Percent identity: 57.00
Percent similarity: 67.78

Results for ZmAGO5C vs AtAGO7 :
Alignment length: 1071
Identical residues: 260
Similar residues: 142
Percent identity: 24.28
Percent similarity: 37.54

Results for ZmAGO5C vs AtAGO8 :
Alignment length: 932
Identical residues: 455
Similar residues: 110
Percent identity: 48.82
Percent similarity: 60.62

Results for ZmAGO5C vs AtAGO9 :
Alignment length: 934
Identical residues: 506
Similar residues: 115
Percent identity: 54.18
Percent similarity: 66.49

Results for ZmAGO5C vs AtAGO10 :
Alignment length: 1051
Identical residues: 289
Similar residues: 138
Percent identity: 27.50
Percent similarity: 40.63

Results for ZmAGO5C vs HvAGO4A :
Alignment length: 957
Identical residues: 527
Similar residues: 102
Percent identity: 55.07
Percent similarity: 65.73

Results for ZmAGO5C vs HvAGO4B :
Alignment length: 950
Identical residues: 422
Similar residues: 104
Percent identity: 44.42
Percent similarity: 55.37

Results for ZmAGO5C vs HvAGO7 :
Alignment length: 1084
Identical residues: 248
Similar residues: 150
Percent identity: 22.88
Percent similarity: 36.72

Results for ZmAGO5C vs HvAGO1D :
Alignment length: 1128
Identical residues: 303
Similar residues: 126
Percent identity: 26.86
Percent similarity: 38.03

Results for ZmAGO5C vs HvAGO5B :
Alignment length: 982
Identical residues: 277
Similar residues: 131
Percent identity: 28.21
Percent similarity: 41.55

Results for ZmAGO5C vs HvAGO6 :
Alignment length: 923
Identical residues: 748
Similar residues: 53
Percent identity: 81.04
Percent similarity: 86.78

Results for ZmAGO5C vs HvAGO2 :
Alignment length: 1137
Identical residues: 248
Similar residues: 128
Percent identity: 21.81
Percent similarity: 33.07

Results for ZmAGO5C vs HvAGO18 :
Alignment length: 1127
Identical residues: 289
Similar residues: 135
Percent identity: 25.64
Percent similarity: 37.62

Results for ZmAGO5C vs HvAGO5A :
Alignment length: 1101
Identical residues: 296
Similar residues: 127
Percent identity: 26.88
Percent similarity: 38.42

Results for ZmAGO5C vs HvAGO10 :
Alignment length: 1024
Identical residues: 296
Similar residues: 132
Percent identity: 28.91
Percent similarity: 41.80

Results for ZmAGO5C vs HvAGO1A :
Alignment length: 1265
Identical residues: 284
Similar residues: 144
Percent identity: 22.45
Percent similarity: 33.83

Results for ZmAGO10B vs ZmAGO18A :
Alignment length: 1103
Identical residues: 447
Similar residues: 126
Percent identity: 40.53
Percent similarity: 51.95

Results for ZmAGO10B vs ZmAGO4D :
Alignment length: 1043
Identical residues: 297
Similar residues: 150
Percent identity: 28.48
Percent similarity: 42.86

Results for ZmAGO10B vs ZmAGO5A :
Alignment length: 1078
Identical residues: 503
Similar residues: 152
Percent identity: 46.66
Percent similarity: 60.76

Results for ZmAGO10B vs ZmAGO18B :
Alignment length: 1086
Identical residues: 379
Similar residues: 134
Percent identity: 34.90
Percent similarity: 47.24

Results for ZmAGO10B vs OsAGO1A :
Alignment length: 1088
Identical residues: 666
Similar residues: 88
Percent identity: 61.21
Percent similarity: 69.30

Results for ZmAGO10B vs OsAGO1B :
Alignment length: 1123
Identical residues: 669
Similar residues: 86
Percent identity: 59.57
Percent similarity: 67.23

Results for ZmAGO10B vs OsAGO1C :
Alignment length: 1026
Identical residues: 640
Similar residues: 93
Percent identity: 62.38
Percent similarity: 71.44

Results for ZmAGO10B vs OsAGO1D :
Alignment length: 1053
Identical residues: 630
Similar residues: 103
Percent identity: 59.83
Percent similarity: 69.61

Results for ZmAGO10B vs OsAGO2 :
Alignment length: 1134
Identical residues: 268
Similar residues: 154
Percent identity: 23.63
Percent similarity: 37.21

Results for ZmAGO10B vs OsAGO3 :
Alignment length: 1189
Identical residues: 267
Similar residues: 144
Percent identity: 22.46
Percent similarity: 34.57

Results for ZmAGO10B vs OsAGO4A :
Alignment length: 1027
Identical residues: 293
Similar residues: 149
Percent identity: 28.53
Percent similarity: 43.04

Results for ZmAGO10B vs OsAGO4B :
Alignment length: 1032
Identical residues: 301
Similar residues: 151
Percent identity: 29.17
Percent similarity: 43.80

Results for ZmAGO10B vs OsAGO7 :
Alignment length: 1120
Identical residues: 317
Similar residues: 151
Percent identity: 28.30
Percent similarity: 41.79

Results for ZmAGO10B vs OsAGO11 :
Alignment length: 1044
Identical residues: 415
Similar residues: 124
Percent identity: 39.75
Percent similarity: 51.63

Results for ZmAGO10B vs OsAGO12 :
Alignment length: 1080
Identical residues: 490
Similar residues: 158
Percent identity: 45.37
Percent similarity: 60.00

Results for ZmAGO10B vs OsAGO13 :
Alignment length: 1361
Identical residues: 430
Similar residues: 116
Percent identity: 31.59
Percent similarity: 40.12

Results for ZmAGO10B vs OsAGO14 :
Alignment length: 1090
Identical residues: 482
Similar residues: 144
Percent identity: 44.22
Percent similarity: 57.43

Results for ZmAGO10B vs OsAGO15 :
Alignment length: 1030
Identical residues: 293
Similar residues: 137
Percent identity: 28.45
Percent similarity: 41.75

Results for ZmAGO10B vs OsAGO16 :
Alignment length: 1023
Identical residues: 299
Similar residues: 137
Percent identity: 29.23
Percent similarity: 42.62

Results for ZmAGO10B vs OsAGO17 :
Alignment length: 983
Identical residues: 440
Similar residues: 125
Percent identity: 44.76
Percent similarity: 57.48

Results for ZmAGO10B vs OsAGO18 :
Alignment length: 1134
Identical residues: 419
Similar residues: 141
Percent identity: 36.95
Percent similarity: 49.38

Results for ZmAGO10B vs OsMEL1 :
Alignment length: 1077
Identical residues: 509
Similar residues: 129
Percent identity: 47.26
Percent similarity: 59.24

Results for ZmAGO10B vs OsPNH1 :
Alignment length: 980
Identical residues: 883
Similar residues: 19
Percent identity: 90.10
Percent similarity: 92.04

Results for ZmAGO10B vs AtAGO1 :
Alignment length: 1066
Identical residues: 656
Similar residues: 81
Percent identity: 61.54
Percent similarity: 69.14

Results for ZmAGO10B vs AtAGO2 :
Alignment length: 1103
Identical residues: 283
Similar residues: 158
Percent identity: 25.66
Percent similarity: 39.98

Results for ZmAGO10B vs AtAGO3 :
Alignment length: 1254
Identical residues: 277
Similar residues: 158
Percent identity: 22.09
Percent similarity: 34.69

Results for ZmAGO10B vs AtAGO4 :
Alignment length: 1035
Identical residues: 295
Similar residues: 162
Percent identity: 28.50
Percent similarity: 44.15

Results for ZmAGO10B vs AtAGO5 :
Alignment length: 1044
Identical residues: 496
Similar residues: 134
Percent identity: 47.51
Percent similarity: 60.34

Results for ZmAGO10B vs AtAGO6 :
Alignment length: 1021
Identical residues: 301
Similar residues: 131
Percent identity: 29.48
Percent similarity: 42.31

Results for ZmAGO10B vs AtAGO7 :
Alignment length: 1102
Identical residues: 314
Similar residues: 134
Percent identity: 28.49
Percent similarity: 40.65

Results for ZmAGO10B vs AtAGO8 :
Alignment length: 1011
Identical residues: 274
Similar residues: 135
Percent identity: 27.10
Percent similarity: 40.45

Results for ZmAGO10B vs AtAGO9 :
Alignment length: 1023
Identical residues: 291
Similar residues: 163
Percent identity: 28.45
Percent similarity: 44.38

Results for ZmAGO10B vs AtAGO10 :
Alignment length: 990
Identical residues: 713
Similar residues: 89
Percent identity: 72.02
Percent similarity: 81.01

Results for ZmAGO10B vs HvAGO4A :
Alignment length: 1042
Identical residues: 298
Similar residues: 145
Percent identity: 28.60
Percent similarity: 42.51

Results for ZmAGO10B vs HvAGO4B :
Alignment length: 1027
Identical residues: 268
Similar residues: 129
Percent identity: 26.10
Percent similarity: 38.66

Results for ZmAGO10B vs HvAGO7 :
Alignment length: 1094
Identical residues: 309
Similar residues: 141
Percent identity: 28.24
Percent similarity: 41.13

Results for ZmAGO10B vs HvAGO1D :

Alignment length: 1071
Identical residues: 627
Similar residues: 113
Percent identity: 58.54
Percent similarity: 69.09

Results for ZmAGO10B vs HvAGO5B :
Alignment length: 990
Identical residues: 478
Similar residues: 120
Percent identity: 48.28
Percent similarity: 60.40

Results for ZmAGO10B vs HvAGO6 :
Alignment length: 1023
Identical residues: 299
Similar residues: 139
Percent identity: 29.23
Percent similarity: 42.82

Results for ZmAGO10B vs HvAGO2 :
Alignment length: 1135
Identical residues: 272
Similar residues: 147
Percent identity: 23.96
Percent similarity: 36.92

Results for ZmAGO10B vs HvAGO18 :
Alignment length: 1097
Identical residues: 453
Similar residues: 139
Percent identity: 41.29
Percent similarity: 53.97

Results for ZmAGO10B vs HvAGO5A :
Alignment length: 1069
Identical residues: 472
Similar residues: 160
Percent identity: 44.15
Percent similarity: 59.12

Results for ZmAGO10B vs HvAGO10 :
Alignment length: 972
Identical residues: 745
Similar residues: 73
Percent identity: 76.65
Percent similarity: 84.16

Results for ZmAGO10B vs HvAGO1A :
Alignment length: 1230
Identical residues: 630
Similar residues: 93
Percent identity: 51.22
Percent similarity: 58.78

Results for ZmAGO18A vs ZmAGO4D :
Alignment length: 1136
Identical residues: 285
Similar residues: 154
Percent identity: 25.09
Percent similarity: 38.64

Results for ZmAGO18A vs ZmAGO5A :
Alignment length: 1122
Identical residues: 437
Similar residues: 151
Percent identity: 38.95
Percent similarity: 52.41

Results for ZmAGO18A vs ZmAGO18B :
Alignment length: 1125
Identical residues: 489
Similar residues: 121
Percent identity: 43.47
Percent similarity: 54.22

Results for ZmAGO18A vs OsAGO1A :
Alignment length: 1146
Identical residues: 472
Similar residues: 143
Percent identity: 41.19
Percent similarity: 53.66

Results for ZmAGO18A vs OsAGO1B :
Alignment length: 1173
Identical residues: 483
Similar residues: 131
Percent identity: 41.18
Percent similarity: 52.34

Results for ZmAGO18A vs OsAGO1C :
Alignment length: 1130
Identical residues: 456
Similar residues: 121
Percent identity: 40.35
Percent similarity: 51.06

Results for ZmAGO18A vs OsAGO1D :
Alignment length: 1130
Identical residues: 459
Similar residues: 139
Percent identity: 40.62
Percent similarity: 52.92

Results for ZmAGO18A vs OsAGO2 :
Alignment length: 1147
Identical residues: 306
Similar residues: 162
Percent identity: 26.68
Percent similarity: 40.80

Results for ZmAGO18A vs OsAGO3 :
Alignment length: 1202
Identical residues: 284
Similar residues: 159
Percent identity: 23.63
Percent similarity: 36.86

Results for ZmAGO18A vs OsAGO4A :
Alignment length: 1135
Identical residues: 282
Similar residues: 152
Percent identity: 24.85
Percent similarity: 38.24

Results for ZmAGO18A vs OsAGO4B :
Alignment length: 1136
Identical residues: 285
Similar residues: 157
Percent identity: 25.09
Percent similarity: 38.91

Results for ZmAGO18A vs OsAGO7 :
Alignment length: 1139
Identical residues: 295
Similar residues: 172
Percent identity: 25.90
Percent similarity: 41.00

Results for ZmAGO18A vs OsAGO11 :
Alignment length: 1105
Identical residues: 351
Similar residues: 130
Percent identity: 31.76
Percent similarity: 43.53

Results for ZmAGO18A vs OsAGO12 :
Alignment length: 1114
Identical residues: 425
Similar residues: 149
Percent identity: 38.15
Percent similarity: 51.53

Results for ZmAGO18A vs OsAGO13 :
Alignment length: 1463
Identical residues: 352
Similar residues: 124
Percent identity: 24.06
Percent similarity: 32.54

Results for ZmAGO18A vs OsAGO14 :
Alignment length: 1124
Identical residues: 408
Similar residues: 151
Percent identity: 36.30
Percent similarity: 49.73

Results for ZmAGO18A vs OsAGO15 :
Alignment length: 1136
Identical residues: 274
Similar residues: 153
Percent identity: 24.12
Percent similarity: 37.59

Results for ZmAGO18A vs OsAGO16 :
Alignment length: 1129
Identical residues: 277
Similar residues: 150
Percent identity: 24.53
Percent similarity: 37.82

Results for ZmAGO18A vs OsAGO17 :
Alignment length: 1107
Identical residues: 333
Similar residues: 137
Percent identity: 30.08
Percent similarity: 42.46

Results for ZmAGO18A vs OsAGO18 :
Alignment length: 1106
Identical residues: 586
Similar residues: 126
Percent identity: 52.98
Percent similarity: 64.38

Results for ZmAGO18A vs OsMEL1 :
Alignment length: 1117
Identical residues: 435

Similar residues: 143
Percent identity: 38.94
Percent similarity: 51.75

Results for ZmAGO18A vs OsPNH1 :
Alignment length: 1111
Identical residues: 455
Similar residues: 133
Percent identity: 40.95
Percent similarity: 52.93

Results for ZmAGO18A vs AtAGO1 :
Alignment length: 1136
Identical residues: 476
Similar residues: 131
Percent identity: 41.90
Percent similarity: 53.43

Results for ZmAGO18A vs AtAGO2 :
Alignment length: 1138
Identical residues: 289
Similar residues: 165
Percent identity: 25.40
Percent similarity: 39.89

Results for ZmAGO18A vs AtAGO3 :
Alignment length: 1263
Identical residues: 300
Similar residues: 167
Percent identity: 23.75
Percent similarity: 36.98

Results for ZmAGO18A vs AtAGO4 :
Alignment length: 1148
Identical residues: 273
Similar residues: 173
Percent identity: 23.78
Percent similarity: 38.85

Results for ZmAGO18A vs AtAGO5 :
Alignment length: 1109
Identical residues: 401
Similar residues: 154
Percent identity: 36.16
Percent similarity: 50.05

Results for ZmAGO18A vs AtAGO6 :
Alignment length: 1127
Identical residues: 277
Similar residues: 149
Percent identity: 24.58
Percent similarity: 37.80

Results for ZmAGO18A vs AtAGO7 :
Alignment length: 1139
Identical residues: 279
Similar residues: 147
Percent identity: 24.50
Percent similarity: 37.40

Results for ZmAGO18A vs AtAGO8 :
Alignment length: 1119
Identical residues: 253
Similar residues: 143
Percent identity: 22.61
Percent similarity: 35.39

Results for ZmAGO18A vs AtAGO9 :
Alignment length: 1130
Identical residues: 274
Similar residues: 164
Percent identity: 24.25
Percent similarity: 38.76

Results for ZmAGO18A vs AtAGO10 :
Alignment length: 1117
Identical residues: 437
Similar residues: 140
Percent identity: 39.12
Percent similarity: 51.66

Results for ZmAGO18A vs HvAGO4A :
Alignment length: 1138
Identical residues: 285
Similar residues: 156
Percent identity: 25.04
Percent similarity: 38.75

Results for ZmAGO18A vs HvAGO4B :
Alignment length: 1133
Identical residues: 251
Similar residues: 142
Percent identity: 22.15
Percent similarity: 34.69

Results for ZmAGO18A vs HvAGO7 :
Alignment length: 1136
Identical residues: 288
Similar residues: 162
Percent identity: 25.35
Percent similarity: 39.61

Results for ZmAGO18A vs HvAGO1D :
Alignment length: 1151
Identical residues: 467
Similar residues: 134
Percent identity: 40.57
Percent similarity: 52.22

Results for ZmAGO18A vs HvAGO5B :
Alignment length: 1087
Identical residues: 387
Similar residues: 129
Percent identity: 35.60
Percent similarity: 47.47

Results for ZmAGO18A vs HvAGO6 :
Alignment length: 1129
Identical residues: 279
Similar residues: 145
Percent identity: 24.71
Percent similarity: 37.56

Results for ZmAGO18A vs HvAGO2 :
Alignment length: 1145
Identical residues: 296
Similar residues: 160
Percent identity: 25.85
Percent similarity: 39.83

Results for ZmAGO18A vs HvAGO18 :
Alignment length: 1078
Identical residues: 643
Similar residues: 98
Percent identity: 59.65
Percent similarity: 68.74

Results for ZmAGO18A vs HvAGO5A :
Alignment length: 1112
Identical residues: 429
Similar residues: 142
Percent identity: 38.58
Percent similarity: 51.35

Results for ZmAGO18A vs HvAGO10 :
Alignment length: 1104
Identical residues: 422
Similar residues: 146
Percent identity: 38.22
Percent similarity: 51.45

Results for ZmAGO18A vs HvAGO1A :
Alignment length: 1286
Identical residues: 439
Similar residues: 127
Percent identity: 34.14
Percent similarity: 44.01

Results for ZmAGO4D vs ZmAGO5A :
Alignment length: 1116
Identical residues: 294
Similar residues: 155
Percent identity: 26.34
Percent similarity: 40.23

Results for ZmAGO4D vs ZmAGO18B :
Alignment length: 1071
Identical residues: 243
Similar residues: 157
Percent identity: 22.69
Percent similarity: 37.35

Results for ZmAGO4D vs OsAGO1A :
Alignment length: 1138
Identical residues: 307
Similar residues: 152
Percent identity: 26.98
Percent similarity: 40.33

Results for ZmAGO4D vs OsAGO1B :
Alignment length: 1171
Identical residues: 305
Similar residues: 152
Percent identity: 26.05
Percent similarity: 39.03

Results for ZmAGO4D vs OsAGO1C :
Alignment length: 1082
Identical residues: 306
Similar residues: 135
Percent identity: 28.28
Percent similarity: 40.76

Results for ZmAGO4D vs OsAGO1D :
Alignment length: 1096
Identical residues: 305
Similar residues: 142
Percent identity: 27.83

Percent similarity: 40.78

Results for ZmAGO4D vs OsAGO2 :

Alignment length: 1140
Identical residues: 249
Similar residues: 127
Percent identity: 21.84
Percent similarity: 32.98

Results for ZmAGO4D vs OsAGO3 :

Alignment length: 1199
Identical residues: 238
Similar residues: 131
Percent identity: 19.85
Percent similarity: 30.78

Results for ZmAGO4D vs OsAGO4A :

Alignment length: 927
Identical residues: 682
Similar residues: 102
Percent identity: 73.57
Percent similarity: 84.57

Results for ZmAGO4D vs OsAGO4B :

Alignment length: 926
Identical residues: 767
Similar residues: 65
Percent identity: 82.83
Percent similarity: 89.85

Results for ZmAGO4D vs OsAGO7 :

Alignment length: 1121
Identical residues: 245
Similar residues: 158
Percent identity: 21.86
Percent similarity: 35.95

Results for ZmAGO4D vs OsAGO11 :

Alignment length: 1063
Identical residues: 260
Similar residues: 121
Percent identity: 24.46
Percent similarity: 35.84

Results for ZmAGO4D vs OsAGO12 :

Alignment length: 1117
Identical residues: 290
Similar residues: 151
Percent identity: 25.96
Percent similarity: 39.48

Results for ZmAGO4D vs OsAGO13 :

Alignment length: 1359
Identical residues: 259
Similar residues: 133
Percent identity: 19.06
Percent similarity: 28.84

Results for ZmAGO4D vs OsAGO14 :

Alignment length: 1132
Identical residues: 289
Similar residues: 144
Percent identity: 25.53
Percent similarity: 38.25

Results for ZmAGO4D vs OsAGO15 :

Alignment length: 943
Identical residues: 534
Similar residues: 107
Percent identity: 56.63
Percent similarity: 67.97

Results for ZmAGO4D vs OsAGO16 :

Alignment length: 929
Identical residues: 492
Similar residues: 105
Percent identity: 52.96
Percent similarity: 64.26

Results for ZmAGO4D vs OsAGO17 :

Alignment length: 992
Identical residues: 244
Similar residues: 152
Percent identity: 24.60
Percent similarity: 39.92

Results for ZmAGO4D vs OsAGO18 :

Alignment length: 1169
Identical residues: 268
Similar residues: 150
Percent identity: 22.93
Percent similarity: 35.76

Results for ZmAGO4D vs OsMEL1 :

Alignment length: 1119
Identical residues: 300
Similar residues: 145
Percent identity: 26.81
Percent similarity: 39.77

Results for ZmAGO4D vs OsPNH1 :

Alignment length: 1054
Identical residues: 298
Similar residues: 154
Percent identity: 28.27
Percent similarity: 42.88

Results for ZmAGO4D vs AtAGO1 :

Alignment length: 1109
Identical residues: 307
Similar residues: 143
Percent identity: 27.68
Percent similarity: 40.58

Results for ZmAGO4D vs AtAGO2 :

Alignment length: 1099
Identical residues: 235
Similar residues: 148
Percent identity: 21.38
Percent similarity: 34.85

Results for ZmAGO4D vs AtAGO3 :

Alignment length: 1276
Identical residues: 237
Similar residues: 149
Percent identity: 18.57
Percent similarity: 30.25

Results for ZmAGO4D vs AtAGO4 :

Alignment length: 946
Identical residues: 576
Similar residues: 125
Percent identity: 60.89
Percent similarity: 74.10

Results for ZmAGO4D vs AtAGO5 :

Alignment length: 1092
Identical residues: 291
Similar residues: 130
Percent identity: 26.65
Percent similarity: 38.55

Results for ZmAGO4D vs AtAGO6 :

Alignment length: 933
Identical residues: 457
Similar residues: 110
Percent identity: 48.98
Percent similarity: 60.77

Results for ZmAGO4D vs AtAGO7 :

Alignment length: 1076
Identical residues: 250
Similar residues: 140
Percent identity: 23.23
Percent similarity: 36.25

Results for ZmAGO4D vs AtAGO8 :

Alignment length: 932
Identical residues: 486
Similar residues: 118
Percent identity: 52.15
Percent similarity: 64.81

Results for ZmAGO4D vs AtAGO9 :

Alignment length: 934
Identical residues: 554
Similar residues: 109
Percent identity: 59.31
Percent similarity: 70.99

Results for ZmAGO4D vs AtAGO10 :

Alignment length: 1057
Identical residues: 295
Similar residues: 156
Percent identity: 27.91
Percent similarity: 42.67

Results for ZmAGO4D vs HvAGO4A :

Alignment length: 938
Identical residues: 668
Similar residues: 113
Percent identity: 71.22
Percent similarity: 83.26

Results for ZmAGO4D vs HvAGO4B :

Alignment length: 948
Identical residues: 483
Similar residues: 87
Percent identity: 50.95
Percent similarity: 60.13

Results for ZmAGO4D vs HvAGO7 :

Alignment length: 1087
Identical residues: 239
Similar residues: 152
Percent identity: 21.99
Percent similarity: 35.97

Results for ZmAGO4D vs HvAGO1D :
Alignment length: 1132
Identical residues: 299
Similar residues: 145
Percent identity: 26.41
Percent similarity: 39.22

Results for ZmAGO4D vs HvAGO5B :
Alignment length: 986
Identical residues: 276
Similar residues: 142
Percent identity: 27.99
Percent similarity: 42.39

Results for ZmAGO4D vs HvAGO6 :
Alignment length: 929
Identical residues: 497
Similar residues: 108
Percent identity: 53.50
Percent similarity: 65.12

Results for ZmAGO4D vs HvAGO2 :
Alignment length: 1148
Identical residues: 252
Similar residues: 131
Percent identity: 21.95
Percent similarity: 33.36

Results for ZmAGO4D vs HvAGO18 :
Alignment length: 1131
Identical residues: 283
Similar residues: 148
Percent identity: 25.02
Percent similarity: 38.11

Results for ZmAGO4D vs HvAGO5A :
Alignment length: 1109
Identical residues: 293
Similar residues: 140
Percent identity: 26.42
Percent similarity: 39.04

Results for ZmAGO4D vs HvAGO10 :
Alignment length: 1033
Identical residues: 288
Similar residues: 153
Percent identity: 27.88
Percent similarity: 42.69

Results for ZmAGO4D vs HvAGO1A :
Alignment length: 1269
Identical residues: 279
Similar residues: 157
Percent identity: 21.99
Percent similarity: 34.36

Results for ZmAGO5A vs ZmAGO18B :
Alignment length: 1146
Identical residues: 342
Similar residues: 150
Percent identity: 29.84
Percent similarity: 42.93

Results for ZmAGO5A vs OsAGO1A :
Alignment length: 1130
Identical residues: 527
Similar residues: 158
Percent identity: 46.64
Percent similarity: 60.62

Results for ZmAGO5A vs OsAGO1B :
Alignment length: 1154
Identical residues: 534
Similar residues: 154
Percent identity: 46.27
Percent similarity: 59.62

Results for ZmAGO5A vs OsAGO1C :
Alignment length: 1090
Identical residues: 506
Similar residues: 143
Percent identity: 46.42
Percent similarity: 59.54

Results for ZmAGO5A vs OsAGO1D :
Alignment length: 1093
Identical residues: 522
Similar residues: 147
Percent identity: 47.76
Percent similarity: 61.21

Results for ZmAGO5A vs OsAGO2 :
Alignment length: 1155
Identical residues: 309
Similar residues: 148
Percent identity: 26.75
Percent similarity: 39.57

Results for ZmAGO5A vs OsAGO3 :
Alignment length: 1207
Identical residues: 282
Similar residues: 162
Percent identity: 23.36
Percent similarity: 36.79

Results for ZmAGO5A vs OsAGO4A :
Alignment length: 1115
Identical residues: 302
Similar residues: 145
Percent identity: 27.09
Percent similarity: 40.09

Results for ZmAGO5A vs OsAGO4B :
Alignment length: 1117
Identical residues: 301
Similar residues: 152
Percent identity: 26.95
Percent similarity: 40.56

Results for ZmAGO5A vs OsAGO7 :
Alignment length: 1147
Identical residues: 335
Similar residues: 158
Percent identity: 29.21
Percent similarity: 42.98

Results for ZmAGO5A vs OsAGO11 :
Alignment length: 1064
Identical residues: 595
Similar residues: 98
Percent identity: 55.92
Percent similarity: 65.13

Results for ZmAGO5A vs OsAGO12 :
Alignment length: 1068
Identical residues: 695
Similar residues: 121
Percent identity: 65.07
Percent similarity: 76.40

Results for ZmAGO5A vs OsAGO13 :
Alignment length: 1445
Identical residues: 460
Similar residues: 121
Percent identity: 31.83
Percent similarity: 40.21

Results for ZmAGO5A vs OsAGO14 :
Alignment length: 1086
Identical residues: 524
Similar residues: 145
Percent identity: 48.25
Percent similarity: 61.60

Results for ZmAGO5A vs OsAGO15 :
Alignment length: 1120
Identical residues: 294
Similar residues: 139
Percent identity: 26.25
Percent similarity: 38.66

Results for ZmAGO5A vs OsAGO16 :
Alignment length: 1112
Identical residues: 296
Similar residues: 139
Percent identity: 26.62
Percent similarity: 39.12

Results for ZmAGO5A vs OsAGO17 :
Alignment length: 1078
Identical residues: 354
Similar residues: 152
Percent identity: 32.84
Percent similarity: 46.94

Results for ZmAGO5A vs OsAGO18 :
Alignment length: 1147
Identical residues: 417
Similar residues: 155
Percent identity: 36.36
Percent similarity: 49.87

Results for ZmAGO5A vs OsMEL1 :
Alignment length: 1075
Identical residues: 573
Similar residues: 147
Percent identity: 53.30
Percent similarity: 66.98

Results for ZmAGO5A vs OsPNH1 :
Alignment length: 1078
Identical residues: 515
Similar residues: 161
Percent identity: 47.77
Percent similarity: 62.71

Results for ZmAGO5A vs AtAGO1 :
Alignment length: 1095

Identical residues: 521
Similar residues: 156
Percent identity: 47.58
Percent similarity: 61.83

Results for ZmAGO5A vs AtAGO2 :
Alignment length: 1145
Identical residues: 288
Similar residues: 161
Percent identity: 25.15
Percent similarity: 39.21

Results for ZmAGO5A vs AtAGO3 :
Alignment length: 1269
Identical residues: 299
Similar residues: 158
Percent identity: 23.56
Percent similarity: 36.01

Results for ZmAGO5A vs AtAGO4 :
Alignment length: 1119
Identical residues: 294
Similar residues: 166
Percent identity: 26.27
Percent similarity: 41.11

Results for ZmAGO5A vs AtAGO5 :
Alignment length: 1070
Identical residues: 518
Similar residues: 153
Percent identity: 48.41
Percent similarity: 62.71

Results for ZmAGO5A vs AtAGO6 :
Alignment length: 1105
Identical residues: 290
Similar residues: 130
Percent identity: 26.24
Percent similarity: 38.01

Results for ZmAGO5A vs AtAGO7 :
Alignment length: 1128
Identical residues: 315
Similar residues: 143
Percent identity: 27.93
Percent similarity: 40.60

Results for ZmAGO5A vs AtAGO8 :
Alignment length: 1099
Identical residues: 270
Similar residues: 140
Percent identity: 24.57
Percent similarity: 37.31

Results for ZmAGO5A vs AtAGO9 :
Alignment length: 1110
Identical residues: 291
Similar residues: 160
Percent identity: 26.22
Percent similarity: 40.63

Results for ZmAGO5A vs AtAGO10 :
Alignment length: 1086
Identical residues: 475
Similar residues: 162
Percent identity: 43.74
Percent similarity: 58.66

Results for ZmAGO5A vs HvAGO4A :
Alignment length: 1116
Identical residues: 294
Similar residues: 155
Percent identity: 26.34
Percent similarity: 40.23

Results for ZmAGO5A vs HvAGO4B :
Alignment length: 1117
Identical residues: 261
Similar residues: 133
Percent identity: 23.37
Percent similarity: 35.27

Results for ZmAGO5A vs HvAGO7 :
Alignment length: 1127
Identical residues: 316
Similar residues: 159
Percent identity: 28.04
Percent similarity: 42.15

Results for ZmAGO5A vs HvAGO1D :
Alignment length: 1131
Identical residues: 499
Similar residues: 164
Percent identity: 44.12
Percent similarity: 58.62

Results for ZmAGO5A vs HvAGO5B :
Alignment length: 1071
Identical residues: 487
Similar residues: 122
Percent identity: 45.47
Percent similarity: 56.86

Results for ZmAGO5A vs HvAGO6 :
Alignment length: 1112
Identical residues: 303
Similar residues: 132
Percent identity: 27.25
Percent similarity: 39.12

Results for ZmAGO5A vs HvAGO2 :
Alignment length: 1150
Identical residues: 309
Similar residues: 149
Percent identity: 26.87
Percent similarity: 39.83

Results for ZmAGO5A vs HvAGO18 :
Alignment length: 1118
Identical residues: 434
Similar residues: 147
Percent identity: 38.82
Percent similarity: 51.97

Results for ZmAGO5A vs HvAGO5A :
Alignment length: 1060
Identical residues: 736
Similar residues: 93
Percent identity: 69.43
Percent similarity: 78.21

Results for ZmAGO5A vs HvAGO10 :
Alignment length: 1079
Identical residues: 474
Similar residues: 155
Percent identity: 43.93
Percent similarity: 58.29

Results for ZmAGO5A vs HvAGO1A :
Alignment length: 1250
Identical residues: 497
Similar residues: 161
Percent identity: 39.76
Percent similarity: 52.64

Results for ZmAGO18B vs OsAGO1A :
Alignment length: 1178
Identical residues: 382
Similar residues: 149
Percent identity: 32.43
Percent similarity: 45.08

Results for ZmAGO18B vs OsAGO1B :
Alignment length: 1204
Identical residues: 386
Similar residues: 144
Percent identity: 32.06
Percent similarity: 44.02

Results for ZmAGO18B vs OsAGO1C :
Alignment length: 1125
Identical residues: 366
Similar residues: 148
Percent identity: 32.53
Percent similarity: 45.69

Results for ZmAGO18B vs OsAGO1D :
Alignment length: 1128
Identical residues: 375
Similar residues: 144
Percent identity: 33.24
Percent similarity: 46.01

Results for ZmAGO18B vs OsAGO2 :
Alignment length: 1168
Identical residues: 221
Similar residues: 162
Percent identity: 18.92
Percent similarity: 32.79

Results for ZmAGO18B vs OsAGO3 :
Alignment length: 1232
Identical residues: 223
Similar residues: 148
Percent identity: 18.10
Percent similarity: 30.11

Results for ZmAGO18B vs OsAGO4A :
Alignment length: 1061
Identical residues: 255
Similar residues: 148
Percent identity: 24.03
Percent similarity: 37.98

Results for ZmAGO18B vs OsAGO4B :
Alignment length: 1064
Identical residues: 253
Similar residues: 156

Percent identity: 23.78
Percent similarity: 38.44

Results for ZmAGO18B vs OsAGO7 :
Alignment length: 1159
Identical residues: 251
Similar residues: 159
Percent identity: 21.66
Percent similarity: 35.38

Results for ZmAGO18B vs OsAGO11 :
Alignment length: 1077
Identical residues: 297
Similar residues: 133
Percent identity: 27.58
Percent similarity: 39.93

Results for ZmAGO18B vs OsAGO12 :
Alignment length: 1139
Identical residues: 336
Similar residues: 149
Percent identity: 29.50
Percent similarity: 42.58

Results for ZmAGO18B vs OsAGO13 :
Alignment length: 1391
Identical residues: 301
Similar residues: 139
Percent identity: 21.64
Percent similarity: 31.63

Results for ZmAGO18B vs OsAGO14 :
Alignment length: 1149
Identical residues: 329
Similar residues: 166
Percent identity: 28.63
Percent similarity: 43.08

Results for ZmAGO18B vs OsAGO15 :
Alignment length: 1062
Identical residues: 246
Similar residues: 153
Percent identity: 23.16
Percent similarity: 37.57

Results for ZmAGO18B vs OsAGO16 :
Alignment length: 1051
Identical residues: 240
Similar residues: 155
Percent identity: 22.84
Percent similarity: 37.58

Results for ZmAGO18B vs OsAGO17 :
Alignment length: 1041
Identical residues: 297
Similar residues: 139
Percent identity: 28.53
Percent similarity: 41.88

Results for ZmAGO18B vs OsAGO18 :
Alignment length: 1159
Identical residues: 437
Similar residues: 135
Percent identity: 37.70
Percent similarity: 49.35

Results for ZmAGO18B vs OsMEL1 :
Alignment length: 1141
Identical residues: 353
Similar residues: 162
Percent identity: 30.94
Percent similarity: 45.14

Results for ZmAGO18B vs OsPNH1 :
Alignment length: 1097
Identical residues: 384
Similar residues: 140
Percent identity: 35.00
Percent similarity: 47.77

Results for ZmAGO18B vs AtAGO1 :
Alignment length: 1146
Identical residues: 363
Similar residues: 155
Percent identity: 31.68
Percent similarity: 45.20

Results for ZmAGO18B vs AtAGO2 :
Alignment length: 1137
Identical residues: 216
Similar residues: 173
Percent identity: 19.00
Percent similarity: 34.21

Results for ZmAGO18B vs AtAGO3 :
Alignment length: 1308
Identical residues: 222
Similar residues: 169
Percent identity: 16.97
Percent similarity: 29.89

Results for ZmAGO18B vs AtAGO4 :
Alignment length: 1079
Identical residues: 242
Similar residues: 155
Percent identity: 22.43
Percent similarity: 36.79

Results for ZmAGO18B vs AtAGO5 :
Alignment length: 1108
Identical residues: 335
Similar residues: 153
Percent identity: 30.23
Percent similarity: 44.04

Results for ZmAGO18B vs AtAGO6 :
Alignment length: 1047
Identical residues: 243
Similar residues: 163
Percent identity: 23.21
Percent similarity: 38.78

Results for ZmAGO18B vs AtAGO7 :
Alignment length: 1121
Identical residues: 252
Similar residues: 149
Percent identity: 22.48
Percent similarity: 35.77

Results for ZmAGO18B vs AtAGO8 :
Alignment length: 1041
Identical residues: 232
Similar residues: 136
Percent identity: 22.29
Percent similarity: 35.35

Results for ZmAGO18B vs AtAGO9 :
Alignment length: 1054
Identical residues: 243
Similar residues: 156
Percent identity: 23.06
Percent similarity: 37.86

Results for ZmAGO18B vs AtAGO10 :
Alignment length: 1100
Identical residues: 363
Similar residues: 143
Percent identity: 33.00
Percent similarity: 46.00

Results for ZmAGO18B vs HvAGO4A :
Alignment length: 1066
Identical residues: 251
Similar residues: 153
Percent identity: 23.55
Percent similarity: 37.90

Results for ZmAGO18B vs HvAGO4B :
Alignment length: 1055
Identical residues: 220
Similar residues: 141
Percent identity: 20.85
Percent similarity: 34.22

Results for ZmAGO18B vs HvAGO7 :
Alignment length: 1123
Identical residues: 244
Similar residues: 170
Percent identity: 21.73
Percent similarity: 36.87

Results for ZmAGO18B vs HvAGO1D :
Alignment length: 1163
Identical residues: 369
Similar residues: 147
Percent identity: 31.73
Percent similarity: 44.37

Results for ZmAGO18B vs HvAGO5B :
Alignment length: 1021
Identical residues: 329
Similar residues: 142
Percent identity: 32.22
Percent similarity: 46.13

Results for ZmAGO18B vs HvAGO6 :
Alignment length: 1051
Identical residues: 245
Similar residues: 150
Percent identity: 23.31
Percent similarity: 37.58

Results for ZmAGO18B vs HvAGO2 :
Alignment length: 1175
Identical residues: 218
Similar residues: 167
Percent identity: 18.55
Percent similarity: 32.77

Results for ZmAGO18B vs HvAGO18 :
Alignment length: 1129
Identical residues: 475
Similar residues: 114
Percent identity: 42.07
Percent similarity: 52.17

Results for ZmAGO18B vs HvAGO5A :
Alignment length: 1133
Identical residues: 341
Similar residues: 154
Percent identity: 30.10
Percent similarity: 43.69

Results for ZmAGO18B vs HvAGO10 :
Alignment length: 1075
Identical residues: 346
Similar residues: 153
Percent identity: 32.19
Percent similarity: 46.42

Results for ZmAGO18B vs HvAGO1A :
Alignment length: 1301
Identical residues: 359
Similar residues: 147
Percent identity: 27.59
Percent similarity: 38.89

Results for OsAGO1A vs OsAGO1B :
Alignment length: 1128
Identical residues: 933
Similar residues: 49
Percent identity: 82.71
Percent similarity: 87.06

Results for OsAGO1A vs OsAGO1C :
Alignment length: 1096
Identical residues: 719
Similar residues: 87
Percent identity: 65.60
Percent similarity: 73.54

Results for OsAGO1A vs OsAGO1D :
Alignment length: 1102
Identical residues: 728
Similar residues: 90
Percent identity: 66.06
Percent similarity: 74.23

Results for OsAGO1A vs OsAGO2 :
Alignment length: 1179
Identical residues: 288
Similar residues: 154
Percent identity: 24.43
Percent similarity: 37.49

Results for OsAGO1A vs OsAGO3 :
Alignment length: 1231
Identical residues: 270
Similar residues: 155
Percent identity: 21.93
Percent similarity: 34.52

Results for OsAGO1A vs OsAGO4A :
Alignment length: 1134
Identical residues: 306
Similar residues: 153
Percent identity: 26.98
Percent similarity: 40.48

Results for OsAGO1A vs OsAGO4B :
Alignment length: 1136
Identical residues: 313
Similar residues: 148
Percent identity: 27.55
Percent similarity: 40.58

Results for OsAGO1A vs OsAGO7 :
Alignment length: 1167
Identical residues: 319
Similar residues: 157
Percent identity: 27.34
Percent similarity: 40.79

Results for OsAGO1A vs OsAGO11 :
Alignment length: 1113
Identical residues: 437
Similar residues: 120
Percent identity: 39.26
Percent similarity: 50.04

Results for OsAGO1A vs OsAGO12 :
Alignment length: 1128
Identical residues: 519
Similar residues: 141
Percent identity: 46.01
Percent similarity: 58.51

Results for OsAGO1A vs OsAGO13 :
Alignment length: 1458
Identical residues: 462
Similar residues: 116
Percent identity: 31.69
Percent similarity: 39.64

Results for OsAGO1A vs OsAGO14 :
Alignment length: 1143
Identical residues: 509
Similar residues: 142
Percent identity: 44.53
Percent similarity: 56.96

Results for OsAGO1A vs OsAGO15 :
Alignment length: 1137
Identical residues: 306
Similar residues: 129
Percent identity: 26.91
Percent similarity: 38.26

Results for OsAGO1A vs OsAGO16 :
Alignment length: 1130
Identical residues: 304
Similar residues: 138
Percent identity: 26.90
Percent similarity: 39.12

Results for OsAGO1A vs OsAGO17 :
Alignment length: 1087
Identical residues: 477
Similar residues: 114
Percent identity: 43.88
Percent similarity: 54.37

Results for OsAGO1A vs OsAGO18 :
Alignment length: 1175
Identical residues: 457
Similar residues: 146
Percent identity: 38.89
Percent similarity: 51.32

Results for OsAGO1A vs OsMEL1 :
Alignment length: 1126
Identical residues: 550
Similar residues: 133
Percent identity: 48.85
Percent similarity: 60.66

Results for OsAGO1A vs OsPNH1 :
Alignment length: 1091
Identical residues: 674
Similar residues: 93
Percent identity: 61.78
Percent similarity: 70.30

Results for OsAGO1A vs AtAGO1 :
Alignment length: 1096
Identical residues: 781
Similar residues: 85
Percent identity: 71.26
Percent similarity: 79.01

Results for OsAGO1A vs AtAGO2 :
Alignment length: 1161
Identical residues: 291
Similar residues: 171
Percent identity: 25.06
Percent similarity: 39.79

Results for OsAGO1A vs AtAGO3 :
Alignment length: 1272
Identical residues: 287
Similar residues: 180
Percent identity: 22.56
Percent similarity: 36.71

Results for OsAGO1A vs AtAGO4 :
Alignment length: 1137
Identical residues: 310
Similar residues: 161
Percent identity: 27.26
Percent similarity: 41.42

Results for OsAGO1A vs AtAGO5 :
Alignment length: 1115
Identical residues: 532
Similar residues: 128
Percent identity: 47.71
Percent similarity: 59.19

Results for OsAGO1A vs AtAGO6 :
Alignment length: 1129
Identical residues: 311
Similar residues: 129
Percent identity: 27.55
Percent similarity: 38.97

Results for OsAGO1A vs AtAGO7 :

Alignment length: 1160
Identical residues: 315
Similar residues: 141
Percent identity: 27.16
Percent similarity: 39.31

Results for OsAGO1A vs AtAGO8 :
Alignment length: 1119
Identical residues: 291
Similar residues: 138
Percent identity: 26.01
Percent similarity: 38.34

Results for OsAGO1A vs AtAGO9 :
Alignment length: 1129
Identical residues: 306
Similar residues: 162
Percent identity: 27.10
Percent similarity: 41.45

Results for OsAGO1A vs AtAGO10 :
Alignment length: 1095
Identical residues: 659
Similar residues: 102
Percent identity: 60.18
Percent similarity: 69.50

Results for OsAGO1A vs HvAGO4A :
Alignment length: 1137
Identical residues: 310
Similar residues: 150
Percent identity: 27.26
Percent similarity: 40.46

Results for OsAGO1A vs HvAGO4B :
Alignment length: 1134
Identical residues: 282
Similar residues: 138
Percent identity: 24.87
Percent similarity: 37.04

Results for OsAGO1A vs HvAGO7 :
Alignment length: 1155
Identical residues: 321
Similar residues: 140
Percent identity: 27.79
Percent similarity: 39.91

Results for OsAGO1A vs HvAGO1D :
Alignment length: 1121
Identical residues: 697
Similar residues: 108
Percent identity: 62.18
Percent similarity: 71.81

Results for OsAGO1A vs HvAGO5B :
Alignment length: 1096
Identical residues: 492
Similar residues: 125
Percent identity: 44.89
Percent similarity: 56.30

Results for OsAGO1A vs HvAGO6 :
Alignment length: 1130
Identical residues: 307
Similar residues: 139
Percent identity: 27.17
Percent similarity: 39.47

Results for OsAGO1A vs HvAGO2 :
Alignment length: 1177
Identical residues: 299
Similar residues: 159
Percent identity: 25.40
Percent similarity: 38.91

Results for OsAGO1A vs HvAGO18 :
Alignment length: 1142
Identical residues: 484
Similar residues: 138
Percent identity: 42.38
Percent similarity: 54.47

Results for OsAGO1A vs HvAGO5A :
Alignment length: 1123
Identical residues: 514
Similar residues: 158
Percent identity: 45.77
Percent similarity: 59.84

Results for OsAGO1A vs HvAGO10 :
Alignment length: 1088
Identical residues: 610
Similar residues: 108
Percent identity: 56.07
Percent similarity: 65.99

Results for OsAGO1A vs HvAGO1A :
Alignment length: 1246
Identical residues: 863
Similar residues: 60
Percent identity: 69.26
Percent similarity: 74.08

Results for OsAGO1B vs OsAGO1C :
Alignment length: 1128
Identical residues: 739
Similar residues: 71
Percent identity: 65.51
Percent similarity: 71.81

Results for OsAGO1B vs OsAGO1D :
Alignment length: 1130
Identical residues: 748
Similar residues: 87
Percent identity: 66.19
Percent similarity: 73.89

Results for OsAGO1B vs OsAGO2 :
Alignment length: 1207
Identical residues: 307
Similar residues: 153
Percent identity: 25.43
Percent similarity: 38.11

Results for OsAGO1B vs OsAGO3 :
Alignment length: 1259
Identical residues: 278
Similar residues: 152
Percent identity: 22.08
Percent similarity: 34.15

Results for OsAGO1B vs OsAGO4A :
Alignment length: 1169
Identical residues: 301
Similar residues: 149
Percent identity: 25.75
Percent similarity: 38.49

Results for OsAGO1B vs OsAGO4B :
Alignment length: 1171
Identical residues: 310
Similar residues: 145
Percent identity: 26.47
Percent similarity: 38.86

Results for OsAGO1B vs OsAGO7 :
Alignment length: 1194
Identical residues: 324
Similar residues: 163
Percent identity: 27.14
Percent similarity: 40.79

Results for OsAGO1B vs OsAGO11 :
Alignment length: 1143
Identical residues: 439
Similar residues: 119
Percent identity: 38.41
Percent similarity: 48.82

Results for OsAGO1B vs OsAGO12 :
Alignment length: 1153
Identical residues: 525
Similar residues: 139
Percent identity: 45.53
Percent similarity: 57.59

Results for OsAGO1B vs OsAGO13 :
Alignment length: 1495
Identical residues: 462
Similar residues: 107
Percent identity: 30.90
Percent similarity: 38.06

Results for OsAGO1B vs OsAGO14 :
Alignment length: 1169
Identical residues: 518
Similar residues: 135
Percent identity: 44.31
Percent similarity: 55.86

Results for OsAGO1B vs OsAGO15 :
Alignment length: 1173
Identical residues: 303
Similar residues: 128
Percent identity: 25.83
Percent similarity: 36.74

Results for OsAGO1B vs OsAGO16 :
Alignment length: 1165
Identical residues: 298
Similar residues: 140
Percent identity: 25.58
Percent similarity: 37.60

Results for OsAGO1B vs OsAGO17 :
Alignment length: 1123
Identical residues: 481

Similar residues: 105
Percent identity: 42.83
Percent similarity: 52.18

Results for OsAGO1B vs OsAGO18 :
Alignment length: 1198
Identical residues: 466
Similar residues: 133
Percent identity: 38.90
Percent similarity: 50.00

Results for OsAGO1B vs OsMEL1 :
Alignment length: 1150
Identical residues: 552
Similar residues: 122
Percent identity: 48.00
Percent similarity: 58.61

Results for OsAGO1B vs OsPNH1 :
Alignment length: 1126
Identical residues: 679
Similar residues: 93
Percent identity: 60.30
Percent similarity: 68.56

Results for OsAGO1B vs AtAGO1 :
Alignment length: 1126
Identical residues: 798
Similar residues: 65
Percent identity: 70.87
Percent similarity: 76.64

Results for OsAGO1B vs AtAGO2 :
Alignment length: 1190
Identical residues: 297
Similar residues: 167
Percent identity: 24.96
Percent similarity: 38.99

Results for OsAGO1B vs AtAGO3 :
Alignment length: 1272
Identical residues: 293
Similar residues: 185
Percent identity: 23.03
Percent similarity: 37.58

Results for OsAGO1B vs AtAGO4 :
Alignment length: 1172
Identical residues: 311
Similar residues: 158
Percent identity: 26.54
Percent similarity: 40.02

Results for OsAGO1B vs AtAGO5 :
Alignment length: 1144
Identical residues: 532
Similar residues: 133
Percent identity: 46.50
Percent similarity: 58.13

Results for OsAGO1B vs AtAGO6 :
Alignment length: 1164
Identical residues: 305
Similar residues: 134
Percent identity: 26.20
Percent similarity: 37.71

Results for OsAGO1B vs AtAGO7 :
Alignment length: 1189
Identical residues: 324
Similar residues: 139
Percent identity: 27.25
Percent similarity: 38.94

Results for OsAGO1B vs AtAGO8 :
Alignment length: 1154
Identical residues: 288
Similar residues: 133
Percent identity: 24.96
Percent similarity: 36.48

Results for OsAGO1B vs AtAGO9 :
Alignment length: 1164
Identical residues: 301
Similar residues: 157
Percent identity: 25.86
Percent similarity: 39.35

Results for OsAGO1B vs AtAGO10 :
Alignment length: 1130
Identical residues: 663
Similar residues: 99
Percent identity: 58.67
Percent similarity: 67.43

Results for OsAGO1B vs HvAGO4A :
Alignment length: 1172
Identical residues: 307
Similar residues: 145
Percent identity: 26.19
Percent similarity: 38.57

Results for OsAGO1B vs HvAGO4B :
Alignment length: 1170
Identical residues: 281
Similar residues: 135
Percent identity: 24.02
Percent similarity: 35.56

Results for OsAGO1B vs HvAGO7 :
Alignment length: 1188
Identical residues: 327
Similar residues: 142
Percent identity: 27.53
Percent similarity: 39.48

Results for OsAGO1B vs HvAGO1D :
Alignment length: 1153
Identical residues: 710
Similar residues: 100
Percent identity: 61.58
Percent similarity: 70.25

Results for OsAGO1B vs HvAGO5B :
Alignment length: 1133
Identical residues: 493
Similar residues: 114
Percent identity: 43.51
Percent similarity: 53.57

Results for OsAGO1B vs HvAGO6 :
Alignment length: 1165
Identical residues: 302
Similar residues: 139
Percent identity: 25.92
Percent similarity: 37.85

Results for OsAGO1B vs HvAGO2 :
Alignment length: 1206
Identical residues: 315
Similar residues: 155
Percent identity: 26.12
Percent similarity: 38.97

Results for OsAGO1B vs HvAGO18 :
Alignment length: 1168
Identical residues: 491
Similar residues: 131
Percent identity: 42.04
Percent similarity: 53.25

Results for OsAGO1B vs HvAGO5A :
Alignment length: 1148
Identical residues: 512
Similar residues: 157
Percent identity: 44.60
Percent similarity: 58.28

Results for OsAGO1B vs HvAGO10 :
Alignment length: 1122
Identical residues: 617
Similar residues: 102
Percent identity: 54.99
Percent similarity: 64.08

Results for OsAGO1B vs HvAGO1A :
Alignment length: 1239
Identical residues: 942
Similar residues: 35
Percent identity: 76.03
Percent similarity: 78.85

Results for OsAGO1C vs OsAGO1D :
Alignment length: 1045
Identical residues: 806
Similar residues: 56
Percent identity: 77.13
Percent similarity: 82.49

Results for OsAGO1C vs OsAGO2 :
Alignment length: 1163
Identical residues: 275
Similar residues: 151
Percent identity: 23.65
Percent similarity: 36.63

Results for OsAGO1C vs OsAGO3 :
Alignment length: 1213
Identical residues: 264
Similar residues: 128
Percent identity: 21.76
Percent similarity: 32.32

Results for OsAGO1C vs OsAGO4A :
Alignment length: 1067
Identical residues: 304
Similar residues: 141
Percent identity: 28.49

Percent similarity: 41.71

Results for OsAGO1C vs OsAGO4B :
Alignment length: 1072
Identical residues: 308
Similar residues: 142
Percent identity: 28.73
Percent similarity: 41.98

Results for OsAGO1C vs OsAGO7 :
Alignment length: 1149
Identical residues: 310
Similar residues: 152
Percent identity: 26.98
Percent similarity: 40.21

Results for OsAGO1C vs OsAGO11 :
Alignment length: 1069
Identical residues: 416
Similar residues: 117
Percent identity: 38.91
Percent similarity: 49.86

Results for OsAGO1C vs OsAGO12 :
Alignment length: 1093
Identical residues: 503
Similar residues: 133
Percent identity: 46.02
Percent similarity: 58.19

Results for OsAGO1C vs OsAGO13 :
Alignment length: 1390
Identical residues: 439
Similar residues: 116
Percent identity: 31.58
Percent similarity: 39.93

Results for OsAGO1C vs OsAGO14 :
Alignment length: 1105
Identical residues: 487
Similar residues: 126
Percent identity: 44.07
Percent similarity: 55.48

Results for OsAGO1C vs OsAGO15 :
Alignment length: 1072
Identical residues: 298
Similar residues: 128
Percent identity: 27.80
Percent similarity: 39.74

Results for OsAGO1C vs OsAGO16 :
Alignment length: 1064
Identical residues: 303
Similar residues: 136
Percent identity: 28.48
Percent similarity: 41.26

Results for OsAGO1C vs OsAGO17 :
Alignment length: 1021
Identical residues: 450
Similar residues: 120
Percent identity: 44.07
Percent similarity: 55.83

Results for OsAGO1C vs OsAGO18 :
Alignment length: 1162
Identical residues: 429
Similar residues: 135
Percent identity: 36.92
Percent similarity: 48.54

Results for OsAGO1C vs OsMEL1 :
Alignment length: 1090
Identical residues: 524
Similar residues: 120
Percent identity: 48.07
Percent similarity: 59.08

Results for OsAGO1C vs OsPNH1 :
Alignment length: 1030
Identical residues: 646
Similar residues: 91
Percent identity: 62.72
Percent similarity: 71.55

Results for OsAGO1C vs AtAGO1 :
Alignment length: 1070
Identical residues: 698
Similar residues: 88
Percent identity: 65.23
Percent similarity: 73.46

Results for OsAGO1C vs AtAGO2 :
Alignment length: 1136
Identical residues: 283
Similar residues: 151
Percent identity: 24.91
Percent similarity: 38.20

Results for OsAGO1C vs AtAGO3 :
Alignment length: 1269
Identical residues: 274
Similar residues: 157
Percent identity: 21.59
Percent similarity: 33.96

Results for OsAGO1C vs AtAGO4 :
Alignment length: 1073
Identical residues: 311
Similar residues: 151
Percent identity: 28.98
Percent similarity: 43.06

Results for OsAGO1C vs AtAGO5 :
Alignment length: 1069
Identical residues: 511
Similar residues: 123
Percent identity: 47.80
Percent similarity: 59.31

Results for OsAGO1C vs AtAGO6 :
Alignment length: 1065
Identical residues: 295
Similar residues: 142
Percent identity: 27.70
Percent similarity: 41.03

Results for OsAGO1C vs AtAGO7 :
Alignment length: 1130
Identical residues: 310
Similar residues: 139
Percent identity: 27.43
Percent similarity: 39.73

Results for OsAGO1C vs AtAGO8 :
Alignment length: 1053
Identical residues: 279
Similar residues: 133
Percent identity: 26.50
Percent similarity: 39.13

Results for OsAGO1C vs AtAGO9 :
Alignment length: 1064
Identical residues: 302
Similar residues: 153
Percent identity: 28.38
Percent similarity: 42.76

Results for OsAGO1C vs AtAGO10 :
Alignment length: 1031
Identical residues: 646
Similar residues: 95
Percent identity: 62.66
Percent similarity: 71.87

Results for OsAGO1C vs HvAGO4A :
Alignment length: 1082
Identical residues: 302
Similar residues: 140
Percent identity: 27.91
Percent similarity: 40.85

Results for OsAGO1C vs HvAGO4B :
Alignment length: 1071
Identical residues: 275
Similar residues: 139
Percent identity: 25.68
Percent similarity: 38.66

Results for OsAGO1C vs HvAGO7 :
Alignment length: 1123
Identical residues: 307
Similar residues: 151
Percent identity: 27.34
Percent similarity: 40.78

Results for OsAGO1C vs HvAGO1D :
Alignment length: 1076
Identical residues: 755
Similar residues: 81
Percent identity: 70.17
Percent similarity: 77.70

Results for OsAGO1C vs HvAGO5B :
Alignment length: 1030
Identical residues: 472
Similar residues: 126
Percent identity: 45.83
Percent similarity: 58.06

Results for OsAGO1C vs HvAGO6 :
Alignment length: 1064
Identical residues: 301
Similar residues: 138
Percent identity: 28.29
Percent similarity: 41.26

Results for OsAGO1C vs HvAGO2 :
Alignment length: 1159
Identical residues: 278
Similar residues: 144
Percent identity: 23.99
Percent similarity: 36.41

Results for OsAGO1C vs HvAGO18 :
Alignment length: 1124
Identical residues: 467
Similar residues: 119
Percent identity: 41.55
Percent similarity: 52.14

Results for OsAGO1C vs HvAGO5A :
Alignment length: 1087
Identical residues: 489
Similar residues: 142
Percent identity: 44.99
Percent similarity: 58.05

Results for OsAGO1C vs HvAGO10 :
Alignment length: 1024
Identical residues: 592
Similar residues: 107
Percent identity: 57.81
Percent similarity: 68.26

Results for OsAGO1C vs HvAGO1A :
Alignment length: 1226
Identical residues: 690
Similar residues: 88
Percent identity: 56.28
Percent similarity: 63.46

Results for OsAGO1D vs OsAGO2 :
Alignment length: 1161
Identical residues: 282
Similar residues: 156
Percent identity: 24.29
Percent similarity: 37.73

Results for OsAGO1D vs OsAGO3 :
Alignment length: 1214
Identical residues: 267
Similar residues: 141
Percent identity: 21.99
Percent similarity: 33.61

Results for OsAGO1D vs OsAGO4A :
Alignment length: 1094
Identical residues: 300
Similar residues: 139
Percent identity: 27.42
Percent similarity: 40.13

Results for OsAGO1D vs OsAGO4B :
Alignment length: 1096
Identical residues: 305
Similar residues: 142
Percent identity: 27.83
Percent similarity: 40.78

Results for OsAGO1D vs OsAGO7 :
Alignment length: 1151
Identical residues: 316
Similar residues: 163
Percent identity: 27.45
Percent similarity: 41.62

Results for OsAGO1D vs OsAGO11 :
Alignment length: 1079
Identical residues: 423
Similar residues: 131
Percent identity: 39.20
Percent similarity: 51.34

Results for OsAGO1D vs OsAGO12 :
Alignment length: 1091
Identical residues: 521
Similar residues: 145
Percent identity: 47.75
Percent similarity: 61.04

Results for OsAGO1D vs OsAGO13 :
Alignment length: 1418
Identical residues: 443
Similar residues: 121
Percent identity: 31.24
Percent similarity: 39.77

Results for OsAGO1D vs OsAGO14 :
Alignment length: 1107
Identical residues: 492
Similar residues: 143
Percent identity: 44.44
Percent similarity: 57.36

Results for OsAGO1D vs OsAGO15 :
Alignment length: 1096
Identical residues: 298
Similar residues: 130
Percent identity: 27.19
Percent similarity: 39.05

Results for OsAGO1D vs OsAGO16 :
Alignment length: 1088
Identical residues: 303
Similar residues: 131
Percent identity: 27.85
Percent similarity: 39.89

Results for OsAGO1D vs OsAGO17 :
Alignment length: 1051
Identical residues: 443
Similar residues: 120
Percent identity: 42.15
Percent similarity: 53.57

Results for OsAGO1D vs OsAGO18 :
Alignment length: 1158
Identical residues: 440
Similar residues: 137
Percent identity: 38.00
Percent similarity: 49.83

Results for OsAGO1D vs OsMEL1 :
Alignment length: 1089
Identical residues: 529
Similar residues: 130
Percent identity: 48.58
Percent similarity: 60.51

Results for OsAGO1D vs OsPNH1 :
Alignment length: 1056
Identical residues: 633
Similar residues: 104
Percent identity: 59.94
Percent similarity: 69.79

Results for OsAGO1D vs AtAGO1 :
Alignment length: 1076
Identical residues: 693
Similar residues: 93
Percent identity: 64.41
Percent similarity: 73.05

Results for OsAGO1D vs AtAGO2 :
Alignment length: 1138
Identical residues: 286
Similar residues: 155
Percent identity: 25.13
Percent similarity: 38.75

Results for OsAGO1D vs AtAGO3 :
Alignment length: 1269
Identical residues: 283
Similar residues: 158
Percent identity: 22.30
Percent similarity: 34.75

Results for OsAGO1D vs AtAGO4 :
Alignment length: 1097
Identical residues: 302
Similar residues: 154
Percent identity: 27.53
Percent similarity: 41.57

Results for OsAGO1D vs AtAGO5 :
Alignment length: 1084
Identical residues: 511
Similar residues: 140
Percent identity: 47.14
Percent similarity: 60.06

Results for OsAGO1D vs AtAGO6 :
Alignment length: 1087
Identical residues: 301
Similar residues: 135
Percent identity: 27.69
Percent similarity: 40.11

Results for OsAGO1D vs AtAGO7 :
Alignment length: 1132
Identical residues: 319
Similar residues: 147
Percent identity: 28.18
Percent similarity: 41.17

Results for OsAGO1D vs AtAGO8 :
Alignment length: 1075
Identical residues: 284
Similar residues: 125
Percent identity: 26.42
Percent similarity: 38.05

Results for OsAGO1D vs AtAGO9 :
Alignment length: 1086

Identical residues: 301
Similar residues: 146
Percent identity: 27.72
Percent similarity: 41.16

Results for OsAGO1D vs AtAGO10 :
Alignment length: 1058
Identical residues: 629
Similar residues: 101
Percent identity: 59.45
Percent similarity: 69.00

Results for OsAGO1D vs HvAGO4A :
Alignment length: 1102
Identical residues: 305
Similar residues: 138
Percent identity: 27.68
Percent similarity: 40.20

Results for OsAGO1D vs HvAGO4B :
Alignment length: 1095
Identical residues: 275
Similar residues: 132
Percent identity: 25.11
Percent similarity: 37.17

Results for OsAGO1D vs HvAGO7 :
Alignment length: 1130
Identical residues: 309
Similar residues: 154
Percent identity: 27.35
Percent similarity: 40.97

Results for OsAGO1D vs HvAGO1D :
Alignment length: 1089
Identical residues: 824
Similar residues: 77
Percent identity: 75.67
Percent similarity: 82.74

Results for OsAGO1D vs HvAGO5B :
Alignment length: 1056
Identical residues: 465
Similar residues: 130
Percent identity: 44.03
Percent similarity: 56.34

Results for OsAGO1D vs HvAGO6 :
Alignment length: 1088
Identical residues: 304
Similar residues: 134
Percent identity: 27.94
Percent similarity: 40.26

Results for OsAGO1D vs HvAGO2 :
Alignment length: 1157
Identical residues: 291
Similar residues: 147
Percent identity: 25.15
Percent similarity: 37.86

Results for OsAGO1D vs HvAGO18 :
Alignment length: 1121
Identical residues: 473
Similar residues: 130
Percent identity: 42.19
Percent similarity: 53.79

Results for OsAGO1D vs HvAGO5A :
Alignment length: 1090
Identical residues: 505
Similar residues: 150
Percent identity: 46.33
Percent similarity: 60.09

Results for OsAGO1D vs HvAGO10 :
Alignment length: 1050
Identical residues: 586
Similar residues: 110
Percent identity: 55.81
Percent similarity: 66.29

Results for OsAGO1D vs HvAGO1A :
Alignment length: 1225
Identical residues: 692
Similar residues: 100
Percent identity: 56.49
Percent similarity: 64.65

Results for OsAGO2 vs OsAGO3 :
Alignment length: 1127
Identical residues: 627
Similar residues: 77
Percent identity: 55.63
Percent similarity: 62.47

Results for OsAGO2 vs OsAGO4A :
Alignment length: 1140
Identical residues: 250
Similar residues: 136
Percent identity: 21.93
Percent similarity: 33.86

Results for OsAGO2 vs OsAGO4B :
Alignment length: 1140
Identical residues: 253
Similar residues: 130
Percent identity: 22.19
Percent similarity: 33.60

Results for OsAGO2 vs OsAGO7 :
Alignment length: 1106
Identical residues: 320
Similar residues: 167
Percent identity: 28.93
Percent similarity: 44.03

Results for OsAGO2 vs OsAGO11 :
Alignment length: 1129
Identical residues: 254
Similar residues: 131
Percent identity: 22.50
Percent similarity: 34.10

Results for OsAGO2 vs OsAGO12 :
Alignment length: 1152
Identical residues: 301
Similar residues: 148
Percent identity: 26.13
Percent similarity: 38.98

Results for OsAGO2 vs OsAGO13 :
Alignment length: 1486
Identical residues: 226
Similar residues: 125
Percent identity: 15.21
Percent similarity: 23.62

Results for OsAGO2 vs OsAGO14 :
Alignment length: 1163
Identical residues: 307
Similar residues: 143
Percent identity: 26.40
Percent similarity: 38.69

Results for OsAGO2 vs OsAGO15 :
Alignment length: 1135
Identical residues: 238
Similar residues: 136
Percent identity: 20.97
Percent similarity: 32.95

Results for OsAGO2 vs OsAGO16 :
Alignment length: 1128
Identical residues: 250
Similar residues: 118
Percent identity: 22.16
Percent similarity: 32.62

Results for OsAGO2 vs OsAGO17 :
Alignment length: 1124
Identical residues: 221
Similar residues: 137
Percent identity: 19.66
Percent similarity: 31.85

Results for OsAGO2 vs OsAGO18 :
Alignment length: 1161
Identical residues: 295
Similar residues: 156
Percent identity: 25.41
Percent similarity: 38.85

Results for OsAGO2 vs OsMEL1 :
Alignment length: 1148
Identical residues: 296
Similar residues: 149
Percent identity: 25.78
Percent similarity: 38.76

Results for OsAGO2 vs OsPNH1 :
Alignment length: 1135
Identical residues: 275
Similar residues: 159
Percent identity: 24.23
Percent similarity: 38.24

Results for OsAGO2 vs AtAGO1 :
Alignment length: 1168
Identical residues: 298
Similar residues: 148
Percent identity: 25.51
Percent similarity: 38.18

Results for OsAGO2 vs AtAGO2 :
Alignment length: 1079
Identical residues: 400
Similar residues: 153

Percent identity: 37.07
Percent similarity: 51.25

Results for OsAGO2 vs AtAGO3 :
Alignment length: 1209
Identical residues: 419
Similar residues: 161
Percent identity: 34.66
Percent similarity: 47.97

Results for OsAGO2 vs AtAGO4 :
Alignment length: 1144
Identical residues: 246
Similar residues: 138
Percent identity: 21.50
Percent similarity: 33.57

Results for OsAGO2 vs AtAGO5 :
Alignment length: 1140
Identical residues: 281
Similar residues: 142
Percent identity: 24.65
Percent similarity: 37.11

Results for OsAGO2 vs AtAGO6 :
Alignment length: 1124
Identical residues: 241
Similar residues: 129
Percent identity: 21.44
Percent similarity: 32.92

Results for OsAGO2 vs AtAGO7 :
Alignment length: 1100
Identical residues: 304
Similar residues: 151
Percent identity: 27.64
Percent similarity: 41.36

Results for OsAGO2 vs AtAGO8 :
Alignment length: 1113
Identical residues: 232
Similar residues: 123
Percent identity: 20.84
Percent similarity: 31.90

Results for OsAGO2 vs AtAGO9 :
Alignment length: 1126
Identical residues: 253
Similar residues: 134
Percent identity: 22.47
Percent similarity: 34.37

Results for OsAGO2 vs AtAGO10 :
Alignment length: 1143
Identical residues: 268
Similar residues: 137
Percent identity: 23.45
Percent similarity: 35.43

Results for OsAGO2 vs HvAGO4A :
Alignment length: 1139
Identical residues: 251
Similar residues: 141
Percent identity: 22.04
Percent similarity: 34.42

Results for OsAGO2 vs HvAGO4B :
Alignment length: 1130
Identical residues: 216
Similar residues: 129
Percent identity: 19.12
Percent similarity: 30.53

Results for OsAGO2 vs HvAGO7 :
Alignment length: 1102
Identical residues: 307
Similar residues: 162
Percent identity: 27.86
Percent similarity: 42.56

Results for OsAGO2 vs HvAGO1D :
Alignment length: 1187
Identical residues: 287
Similar residues: 164
Percent identity: 24.18
Percent similarity: 37.99

Results for OsAGO2 vs HvAGO5B :
Alignment length: 1106
Identical residues: 267
Similar residues: 130
Percent identity: 24.14
Percent similarity: 35.90

Results for OsAGO2 vs HvAGO6 :
Alignment length: 1128
Identical residues: 244
Similar residues: 128
Percent identity: 21.63
Percent similarity: 32.98

Results for OsAGO2 vs HvAGO2 :
Alignment length: 1054
Identical residues: 752
Similar residues: 87
Percent identity: 71.35
Percent similarity: 79.60

Results for OsAGO2 vs HvAGO18 :
Alignment length: 1145
Identical residues: 298
Similar residues: 162
Percent identity: 26.03
Percent similarity: 40.17

Results for OsAGO2 vs HvAGO5A :
Alignment length: 1142
Identical residues: 302
Similar residues: 142
Percent identity: 26.44
Percent similarity: 38.88

Results for OsAGO2 vs HvAGO10 :
Alignment length: 1130
Identical residues: 263
Similar residues: 148
Percent identity: 23.27
Percent similarity: 36.37

Results for OsAGO2 vs HvAGO1A :
Alignment length: 1315
Identical residues: 285
Similar residues: 151
Percent identity: 21.67
Percent similarity: 33.16

Results for OsAGO3 vs OsAGO4A :
Alignment length: 1199
Identical residues: 237
Similar residues: 143
Percent identity: 19.77
Percent similarity: 31.69

Results for OsAGO3 vs OsAGO4B :
Alignment length: 1199
Identical residues: 241
Similar residues: 140
Percent identity: 20.10
Percent similarity: 31.78

Results for OsAGO3 vs OsAGO7 :
Alignment length: 1167
Identical residues: 313
Similar residues: 168
Percent identity: 26.82
Percent similarity: 41.22

Results for OsAGO3 vs OsAGO11 :
Alignment length: 1186
Identical residues: 237
Similar residues: 126
Percent identity: 19.98
Percent similarity: 30.61

Results for OsAGO3 vs OsAGO12 :
Alignment length: 1203
Identical residues: 277
Similar residues: 153
Percent identity: 23.03
Percent similarity: 35.74

Results for OsAGO3 vs OsAGO13 :
Alignment length: 1554
Identical residues: 225
Similar residues: 137
Percent identity: 14.48
Percent similarity: 23.29

Results for OsAGO3 vs OsAGO14 :
Alignment length: 1214
Identical residues: 287
Similar residues: 140
Percent identity: 23.64
Percent similarity: 35.17

Results for OsAGO3 vs OsAGO15 :
Alignment length: 1203
Identical residues: 225
Similar residues: 142
Percent identity: 18.70
Percent similarity: 30.51

Results for OsAGO3 vs OsAGO16 :
Alignment length: 1195
Identical residues: 240
Similar residues: 135
Percent identity: 20.08
Percent similarity: 31.38

Results for OsAGO3 vs OsAGO17 :

Alignment length: 1179

Identical residues: 220

Similar residues: 126

Percent identity: 18.66

Percent similarity: 29.35

Results for OsAGO3 vs OsAGO18 :

Alignment length: 1215

Identical residues: 285

Similar residues: 145

Percent identity: 23.46

Percent similarity: 35.39

Results for OsAGO3 vs OsMEL1 :

Alignment length: 1198

Identical residues: 280

Similar residues: 157

Percent identity: 23.37

Percent similarity: 36.48

Results for OsAGO3 vs OsPNH1 :

Alignment length: 1191

Identical residues: 271

Similar residues: 149

Percent identity: 22.75

Percent similarity: 35.26

Results for OsAGO3 vs AtAGO1 :

Alignment length: 1218

Identical residues: 293

Similar residues: 145

Percent identity: 24.06

Percent similarity: 35.96

Results for OsAGO3 vs AtAGO2 :

Alignment length: 1143

Identical residues: 360

Similar residues: 153

Percent identity: 31.50

Percent similarity: 44.88

Results for OsAGO3 vs AtAGO3 :

Alignment length: 1273

Identical residues: 383

Similar residues: 162

Percent identity: 30.09

Percent similarity: 42.81

Results for OsAGO3 vs AtAGO4 :

Alignment length: 1201

Identical residues: 228

Similar residues: 146

Percent identity: 18.98

Percent similarity: 31.14

Results for OsAGO3 vs AtAGO5 :

Alignment length: 1195

Identical residues: 274

Similar residues: 138

Percent identity: 22.93

Percent similarity: 34.48

Results for OsAGO3 vs AtAGO6 :

Alignment length: 1192

Identical residues: 237

Similar residues: 131

Percent identity: 19.88

Percent similarity: 30.87

Results for OsAGO3 vs AtAGO7 :

Alignment length: 1161

Identical residues: 294

Similar residues: 144

Percent identity: 25.32

Percent similarity: 37.73

Results for OsAGO3 vs AtAGO8 :

Alignment length: 1180

Identical residues: 224

Similar residues: 128

Percent identity: 18.98

Percent similarity: 29.83

Results for OsAGO3 vs AtAGO9 :

Alignment length: 1192

Identical residues: 235

Similar residues: 142

Percent identity: 19.71

Percent similarity: 31.63

Results for OsAGO3 vs AtAGO10 :

Alignment length: 1198

Identical residues: 266

Similar residues: 142

Percent identity: 22.20

Percent similarity: 34.06

Results for OsAGO3 vs HvAGO4A :

Alignment length: 1198

Identical residues: 233

Similar residues: 150

Percent identity: 19.45

Percent similarity: 31.97

Results for OsAGO3 vs HvAGO4B :

Alignment length: 1200

Identical residues: 209

Similar residues: 123

Percent identity: 17.42

Percent similarity: 27.67

Results for OsAGO3 vs HvAGO7 :

Alignment length: 1162

Identical residues: 297

Similar residues: 165

Percent identity: 25.56

Percent similarity: 39.76

Results for OsAGO3 vs HvAGO1D :

Alignment length: 1240

Identical residues: 264

Similar residues: 147

Percent identity: 21.29

Percent similarity: 33.15

Results for OsAGO3 vs HvAGO5B :

Alignment length: 1174

Identical residues: 268

Similar residues: 126

Percent identity: 22.83

Percent similarity: 33.56

Results for OsAGO3 vs HvAGO6 :

Alignment length: 1195

Identical residues: 243

Similar residues: 134

Percent identity: 20.33

Percent similarity: 31.55

Results for OsAGO3 vs HvAGO2 :

Alignment length: 1133

Identical residues: 556

Similar residues: 117

Percent identity: 49.07

Percent similarity: 59.40

Results for OsAGO3 vs HvAGO18 :

Alignment length: 1198

Identical residues: 289

Similar residues: 158

Percent identity: 24.12

Percent similarity: 37.31

Results for OsAGO3 vs HvAGO5A :

Alignment length: 1198

Identical residues: 266

Similar residues: 161

Percent identity: 22.20

Percent similarity: 35.64

Results for OsAGO3 vs HvAGO10 :

Alignment length: 1188

Identical residues: 260

Similar residues: 144

Percent identity: 21.89

Percent similarity: 34.01

Results for OsAGO3 vs HvAGO1A :

Alignment length: 1365

Identical residues: 255

Similar residues: 150

Percent identity: 18.68

Percent similarity: 29.67

Results for OsAGO4A vs OsAGO4B :

Alignment length: 912

Identical residues: 723

Similar residues: 84

Percent identity: 79.28

Percent similarity: 88.49

Results for OsAGO4A vs OsAGO7 :

Alignment length: 1121

Identical residues: 247

Similar residues: 161

Percent identity: 22.03

Percent similarity: 36.40

Results for OsAGO4A vs OsAGO11 :

Alignment length: 1051

Identical residues: 258

Similar residues: 118

Percent identity: 24.55

Percent similarity: 35.78

Results for OsAGO4A vs OsAGO12 :

Alignment length: 1115
Identical residues: 294
Similar residues: 147
Percent identity: 26.37
Percent similarity: 39.55

Results for OsAGO4A vs OsAGO13 :
Alignment length: 1338
Identical residues: 255
Similar residues: 151
Percent identity: 19.06
Percent similarity: 30.34

Results for OsAGO4A vs OsAGO14 :
Alignment length: 1131
Identical residues: 282
Similar residues: 151
Percent identity: 24.93
Percent similarity: 38.28

Results for OsAGO4A vs OsAGO15 :
Alignment length: 923
Identical residues: 555
Similar residues: 106
Percent identity: 60.13
Percent similarity: 71.61

Results for OsAGO4A vs OsAGO16 :
Alignment length: 910
Identical residues: 519
Similar residues: 101
Percent identity: 57.03
Percent similarity: 68.13

Results for OsAGO4A vs OsAGO17 :
Alignment length: 972
Identical residues: 255
Similar residues: 138
Percent identity: 26.23
Percent similarity: 40.43

Results for OsAGO4A vs OsAGO18 :
Alignment length: 1168
Identical residues: 268
Similar residues: 153
Percent identity: 22.95
Percent similarity: 36.04

Results for OsAGO4A vs OsMEL1 :
Alignment length: 1118
Identical residues: 290
Similar residues: 160
Percent identity: 25.94
Percent similarity: 40.25

Results for OsAGO4A vs OsPNH1 :
Alignment length: 1038
Identical residues: 296
Similar residues: 148
Percent identity: 28.52
Percent similarity: 42.77

Results for OsAGO4A vs AtAGO1 :
Alignment length: 1108
Identical residues: 299
Similar residues: 149
Percent identity: 26.99
Percent similarity: 40.43

Results for OsAGO4A vs AtAGO2 :
Alignment length: 1098
Identical residues: 235
Similar residues: 143
Percent identity: 21.40
Percent similarity: 34.43

Results for OsAGO4A vs AtAGO3 :
Alignment length: 1274
Identical residues: 233
Similar residues: 146
Percent identity: 18.29
Percent similarity: 29.75

Results for OsAGO4A vs AtAGO4 :
Alignment length: 929
Identical residues: 606
Similar residues: 109
Percent identity: 65.23
Percent similarity: 76.96

Results for OsAGO4A vs AtAGO5 :
Alignment length: 1075
Identical residues: 287
Similar residues: 138
Percent identity: 26.70
Percent similarity: 39.53

Results for OsAGO4A vs AtAGO6 :
Alignment length: 916
Identical residues: 474
Similar residues: 116
Percent identity: 51.75
Percent similarity: 64.41

Results for OsAGO4A vs AtAGO7 :
Alignment length: 1076
Identical residues: 250
Similar residues: 138
Percent identity: 23.23
Percent similarity: 36.06

Results for OsAGO4A vs AtAGO8 :
Alignment length: 914
Identical residues: 509
Similar residues: 110
Percent identity: 55.69
Percent similarity: 67.72

Results for OsAGO4A vs AtAGO9 :
Alignment length: 917
Identical residues: 579
Similar residues: 106
Percent identity: 63.14
Percent similarity: 74.70

Results for OsAGO4A vs AtAGO10 :
Alignment length: 1041
Identical residues: 295
Similar residues: 152
Percent identity: 28.34
Percent similarity: 42.94

Results for OsAGO4A vs HvAGO4A :
Alignment length: 924
Identical residues: 783
Similar residues: 58
Percent identity: 84.74
Percent similarity: 91.02

Results for OsAGO4A vs HvAGO4B :
Alignment length: 927
Identical residues: 487
Similar residues: 96
Percent identity: 52.54
Percent similarity: 62.89

Results for OsAGO4A vs HvAGO7 :
Alignment length: 1082
Identical residues: 240
Similar residues: 156
Percent identity: 22.18
Percent similarity: 36.60

Results for OsAGO4A vs HvAGO1D :
Alignment length: 1119
Identical residues: 294
Similar residues: 144
Percent identity: 26.27
Percent similarity: 39.14

Results for OsAGO4A vs HvAGO5B :
Alignment length: 964
Identical residues: 275
Similar residues: 147
Percent identity: 28.53
Percent similarity: 43.78

Results for OsAGO4A vs HvAGO6 :
Alignment length: 910
Identical residues: 520
Similar residues: 111
Percent identity: 57.14
Percent similarity: 69.34

Results for OsAGO4A vs HvAGO2 :
Alignment length: 1148
Identical residues: 254
Similar residues: 131
Percent identity: 22.13
Percent similarity: 33.54

Results for OsAGO4A vs HvAGO18 :
Alignment length: 1130
Identical residues: 281
Similar residues: 149
Percent identity: 24.87
Percent similarity: 38.05

Results for OsAGO4A vs HvAGO5A :
Alignment length: 1108
Identical residues: 301
Similar residues: 132
Percent identity: 27.17
Percent similarity: 39.08

Results for OsAGO4A vs HvAGO10 :
Alignment length: 1016
Identical residues: 283

Similar residues: 147
Percent identity: 27.85
Percent similarity: 42.32

Results for OsAGO4A vs HvAGO1A :
Alignment length: 1268
Identical residues: 280
Similar residues: 154
Percent identity: 22.08
Percent similarity: 34.23

Results for OsAGO4B vs OsAGO7 :
Alignment length: 1121
Identical residues: 253
Similar residues: 160
Percent identity: 22.57
Percent similarity: 36.84

Results for OsAGO4B vs OsAGO11 :
Alignment length: 1055
Identical residues: 258
Similar residues: 123
Percent identity: 24.45
Percent similarity: 36.11

Results for OsAGO4B vs OsAGO12 :
Alignment length: 1117
Identical residues: 297
Similar residues: 151
Percent identity: 26.59
Percent similarity: 40.11

Results for OsAGO4B vs OsAGO13 :
Alignment length: 1346
Identical residues: 265
Similar residues: 136
Percent identity: 19.69
Percent similarity: 29.79

Results for OsAGO4B vs OsAGO14 :
Alignment length: 1133
Identical residues: 302
Similar residues: 136
Percent identity: 26.65
Percent similarity: 38.66

Results for OsAGO4B vs OsAGO15 :
Alignment length: 928
Identical residues: 554
Similar residues: 104
Percent identity: 59.70
Percent similarity: 70.91

Results for OsAGO4B vs OsAGO16 :
Alignment length: 914
Identical residues: 514
Similar residues: 101
Percent identity: 56.24
Percent similarity: 67.29

Results for OsAGO4B vs OsAGO17 :
Alignment length: 980
Identical residues: 251
Similar residues: 148
Percent identity: 25.61
Percent similarity: 40.71

Results for OsAGO4B vs OsAGO18 :
Alignment length: 1169
Identical residues: 268
Similar residues: 159
Percent identity: 22.93
Percent similarity: 36.53

Results for OsAGO4B vs OsMEL1 :
Alignment length: 1120
Identical residues: 308
Similar residues: 143
Percent identity: 27.50
Percent similarity: 40.27

Results for OsAGO4B vs OsPNH1 :
Alignment length: 1043
Identical residues: 297
Similar residues: 157
Percent identity: 28.48
Percent similarity: 43.53

Results for OsAGO4B vs AtAGO1 :
Alignment length: 1110
Identical residues: 307
Similar residues: 145
Percent identity: 27.66
Percent similarity: 40.72

Results for OsAGO4B vs AtAGO2 :
Alignment length: 1098
Identical residues: 236
Similar residues: 151
Percent identity: 21.49
Percent similarity: 35.25

Results for OsAGO4B vs AtAGO3 :
Alignment length: 1274
Identical residues: 234
Similar residues: 153
Percent identity: 18.37
Percent similarity: 30.38

Results for OsAGO4B vs AtAGO4 :
Alignment length: 931
Identical residues: 598
Similar residues: 120
Percent identity: 64.23
Percent similarity: 77.12

Results for OsAGO4B vs AtAGO5 :
Alignment length: 1079
Identical residues: 300
Similar residues: 136
Percent identity: 27.80
Percent similarity: 40.41

Results for OsAGO4B vs AtAGO6 :
Alignment length: 918
Identical residues: 478
Similar residues: 110
Percent identity: 52.07
Percent similarity: 64.05

Results for OsAGO4B vs AtAGO7 :
Alignment length: 1076
Identical residues: 254
Similar residues: 144
Percent identity: 23.61
Percent similarity: 36.99

Results for OsAGO4B vs AtAGO8 :
Alignment length: 917
Identical residues: 506
Similar residues: 115
Percent identity: 55.18
Percent similarity: 67.72

Results for OsAGO4B vs AtAGO9 :
Alignment length: 919
Identical residues: 575
Similar residues: 113
Percent identity: 62.57
Percent similarity: 74.86

Results for OsAGO4B vs AtAGO10 :
Alignment length: 1046
Identical residues: 298
Similar residues: 158
Percent identity: 28.49
Percent similarity: 43.59

Results for OsAGO4B vs HvAGO4A :
Alignment length: 924
Identical residues: 718
Similar residues: 90
Percent identity: 77.71
Percent similarity: 87.45

Results for OsAGO4B vs HvAGO4B :
Alignment length: 933
Identical residues: 489
Similar residues: 93
Percent identity: 52.41
Percent similarity: 62.38

Results for OsAGO4B vs HvAGO7 :
Alignment length: 1082
Identical residues: 245
Similar residues: 158
Percent identity: 22.64
Percent similarity: 37.25

Results for OsAGO4B vs HvAGO1D :
Alignment length: 1121
Identical residues: 302
Similar residues: 143
Percent identity: 26.94
Percent similarity: 39.70

Results for OsAGO4B vs HvAGO5B :
Alignment length: 972
Identical residues: 279
Similar residues: 149
Percent identity: 28.70
Percent similarity: 44.03

Results for OsAGO4B vs HvAGO6 :
Alignment length: 914
Identical residues: 515
Similar residues: 108
Percent identity: 56.35

Percent similarity: 68.16

Results for OsAGO4B vs HvAGO2 :

Alignment length: 1148
Identical residues: 262
Similar residues: 126
Percent identity: 22.82
Percent similarity: 33.80

Results for OsAGO4B vs HvAGO18 :

Alignment length: 1131
Identical residues: 292
Similar residues: 147
Percent identity: 25.82
Percent similarity: 38.82

Results for OsAGO4B vs HvAGO5A :

Alignment length: 1110
Identical residues: 301
Similar residues: 140
Percent identity: 27.12
Percent similarity: 39.73

Results for OsAGO4B vs HvAGO10 :

Alignment length: 1021
Identical residues: 286
Similar residues: 156
Percent identity: 28.01
Percent similarity: 43.29

Results for OsAGO4B vs HvAGO1A :

Alignment length: 1270
Identical residues: 290
Similar residues: 148
Percent identity: 22.83
Percent similarity: 34.49

Results for OsAGO7 vs OsAGO11 :

Alignment length: 1130
Identical residues: 267
Similar residues: 130
Percent identity: 23.63
Percent similarity: 35.13

Results for OsAGO7 vs OsAGO12 :

Alignment length: 1146
Identical residues: 327
Similar residues: 165
Percent identity: 28.53
Percent similarity: 42.93

Results for OsAGO7 vs OsAGO13 :

Alignment length: 1489
Identical residues: 262
Similar residues: 143
Percent identity: 17.60
Percent similarity: 27.20

Results for OsAGO7 vs OsAGO14 :

Alignment length: 1154
Identical residues: 308
Similar residues: 164
Percent identity: 26.69
Percent similarity: 40.90

Results for OsAGO7 vs OsAGO15 :

Alignment length: 1122
Identical residues: 244
Similar residues: 149
Percent identity: 21.75
Percent similarity: 35.03

Results for OsAGO7 vs OsAGO16 :

Alignment length: 1116
Identical residues: 245
Similar residues: 161
Percent identity: 21.95
Percent similarity: 36.38

Results for OsAGO7 vs OsAGO17 :

Alignment length: 1106
Identical residues: 253
Similar residues: 141
Percent identity: 22.88
Percent similarity: 35.62

Results for OsAGO7 vs OsAGO18 :

Alignment length: 1160
Identical residues: 298
Similar residues: 167
Percent identity: 25.69
Percent similarity: 40.09

Results for OsAGO7 vs OsMEL1 :

Alignment length: 1142
Identical residues: 337
Similar residues: 177
Percent identity: 29.51
Percent similarity: 45.01

Results for OsAGO7 vs OsPNH1 :

Alignment length: 1127
Identical residues: 322
Similar residues: 156
Percent identity: 28.57
Percent similarity: 42.41

Results for OsAGO7 vs AtAGO1 :

Alignment length: 1160
Identical residues: 328
Similar residues: 163
Percent identity: 28.28
Percent similarity: 42.33

Results for OsAGO7 vs AtAGO2 :

Alignment length: 1092
Identical residues: 319
Similar residues: 159
Percent identity: 29.21
Percent similarity: 43.77

Results for OsAGO7 vs AtAGO3 :

Alignment length: 1236
Identical residues: 326
Similar residues: 160
Percent identity: 26.38
Percent similarity: 39.32

Results for OsAGO7 vs AtAGO4 :

Alignment length: 1124
Identical residues: 249
Similar residues: 167
Percent identity: 22.15
Percent similarity: 37.01

Results for OsAGO7 vs AtAGO5 :

Alignment length: 1137
Identical residues: 320
Similar residues: 155
Percent identity: 28.14
Percent similarity: 41.78

Results for OsAGO7 vs AtAGO6 :

Alignment length: 1113
Identical residues: 247
Similar residues: 150
Percent identity: 22.19
Percent similarity: 35.67

Results for OsAGO7 vs AtAGO7 :

Alignment length: 1060
Identical residues: 551
Similar residues: 120
Percent identity: 51.98
Percent similarity: 63.30

Results for OsAGO7 vs AtAGO8 :

Alignment length: 1105
Identical residues: 234
Similar residues: 137
Percent identity: 21.18
Percent similarity: 33.57

Results for OsAGO7 vs AtAGO9 :

Alignment length: 1114
Identical residues: 252
Similar residues: 169
Percent identity: 22.62
Percent similarity: 37.79

Results for OsAGO7 vs AtAGO10 :

Alignment length: 1132
Identical residues: 311
Similar residues: 145
Percent identity: 27.47
Percent similarity: 40.28

Results for OsAGO7 vs HvAGO4A :

Alignment length: 1121
Identical residues: 250
Similar residues: 161
Percent identity: 22.30
Percent similarity: 36.66

Results for OsAGO7 vs HvAGO4B :

Alignment length: 1121
Identical residues: 237
Similar residues: 139
Percent identity: 21.14
Percent similarity: 33.54

Results for OsAGO7 vs HvAGO7 :

Alignment length: 1056
Identical residues: 816
Similar residues: 60
Percent identity: 77.27
Percent similarity: 82.95

Results for OsAGO7 vs HvAGO1D :
Alignment length: 1173
Identical residues: 316
Similar residues: 174
Percent identity: 26.94
Percent similarity: 41.77

Results for OsAGO7 vs HvAGO5B :
Alignment length: 1109
Identical residues: 312
Similar residues: 131
Percent identity: 28.13
Percent similarity: 39.95

Results for OsAGO7 vs HvAGO6 :
Alignment length: 1116
Identical residues: 242
Similar residues: 165
Percent identity: 21.68
Percent similarity: 36.47

Results for OsAGO7 vs HvAGO2 :
Alignment length: 1111
Identical residues: 324
Similar residues: 165
Percent identity: 29.16
Percent similarity: 44.01

Results for OsAGO7 vs HvAGO18 :
Alignment length: 1128
Identical residues: 309
Similar residues: 164
Percent identity: 27.39
Percent similarity: 41.93

Results for OsAGO7 vs HvAGO5A :
Alignment length: 1141
Identical residues: 327
Similar residues: 153
Percent identity: 28.66
Percent similarity: 42.07

Results for OsAGO7 vs HvAGO10 :
Alignment length: 1114
Identical residues: 302
Similar residues: 160
Percent identity: 27.11
Percent similarity: 41.47

Results for OsAGO7 vs HvAGO1A :
Alignment length: 1307
Identical residues: 293
Similar residues: 159
Percent identity: 22.42
Percent similarity: 34.58

Results for OsAGO11 vs OsAGO12 :
Alignment length: 1067
Identical residues: 551
Similar residues: 95
Percent identity: 51.64
Percent similarity: 60.54

Results for OsAGO11 vs OsAGO13 :
Alignment length: 1361
Identical residues: 377
Similar residues: 121
Percent identity: 27.70
Percent similarity: 36.59

Results for OsAGO11 vs OsAGO14 :
Alignment length: 1083
Identical residues: 414
Similar residues: 126
Percent identity: 38.23
Percent similarity: 49.86

Results for OsAGO11 vs OsAGO15 :
Alignment length: 1055
Identical residues: 254
Similar residues: 120
Percent identity: 24.08
Percent similarity: 35.45

Results for OsAGO11 vs OsAGO16 :
Alignment length: 1038
Identical residues: 249
Similar residues: 111
Percent identity: 23.99
Percent similarity: 34.68

Results for OsAGO11 vs OsAGO17 :
Alignment length: 1009
Identical residues: 316
Similar residues: 128
Percent identity: 31.32
Percent similarity: 44.00

Results for OsAGO11 vs OsAGO18 :
Alignment length: 1145
Identical residues: 342
Similar residues: 127
Percent identity: 29.87
Percent similarity: 40.96

Results for OsAGO11 vs OsMEL1 :
Alignment length: 1072
Identical residues: 459
Similar residues: 138
Percent identity: 42.82
Percent similarity: 55.69

Results for OsAGO11 vs OsPNH1 :
Alignment length: 1047
Identical residues: 423
Similar residues: 123
Percent identity: 40.40
Percent similarity: 52.15

Results for OsAGO11 vs AtAGO1 :
Alignment length: 1081
Identical residues: 424
Similar residues: 127
Percent identity: 39.22
Percent similarity: 50.97

Results for OsAGO11 vs AtAGO2 :
Alignment length: 1109
Identical residues: 236
Similar residues: 140
Percent identity: 21.28
Percent similarity: 33.90

Results for OsAGO11 vs AtAGO3 :
Alignment length: 1255
Identical residues: 246
Similar residues: 130
Percent identity: 19.60
Percent similarity: 29.96

Results for OsAGO11 vs AtAGO4 :
Alignment length: 1052
Identical residues: 249
Similar residues: 144
Percent identity: 23.67
Percent similarity: 37.36

Results for OsAGO11 vs AtAGO5 :
Alignment length: 1032
Identical residues: 427
Similar residues: 123
Percent identity: 41.38
Percent similarity: 53.29

Results for OsAGO11 vs AtAGO6 :
Alignment length: 1034
Identical residues: 242
Similar residues: 111
Percent identity: 23.40
Percent similarity: 34.14

Results for OsAGO11 vs AtAGO7 :
Alignment length: 1093
Identical residues: 259
Similar residues: 125
Percent identity: 23.70
Percent similarity: 35.13

Results for OsAGO11 vs AtAGO8 :
Alignment length: 1028
Identical residues: 230
Similar residues: 114
Percent identity: 22.37
Percent similarity: 33.46

Results for OsAGO11 vs AtAGO9 :
Alignment length: 1042
Identical residues: 246
Similar residues: 129
Percent identity: 23.61
Percent similarity: 35.99

Results for OsAGO11 vs AtAGO10 :
Alignment length: 1057
Identical residues: 410
Similar residues: 128
Percent identity: 38.79
Percent similarity: 50.90

Results for OsAGO11 vs HvAGO4A :
Alignment length: 1056
Identical residues: 254
Similar residues: 124
Percent identity: 24.05
Percent similarity: 35.80

Results for OsAGO11 vs HvAGO4B :
Alignment length: 1033

Identical residues: 227
Similar residues: 106
Percent identity: 21.97
Percent similarity: 32.24

Results for OsAGO11 vs HvAGO7 :
Alignment length: 1096
Identical residues: 266
Similar residues: 121
Percent identity: 24.27
Percent similarity: 35.31

Results for OsAGO11 vs HvAGO1D :
Alignment length: 1107
Identical residues: 416
Similar residues: 134
Percent identity: 37.58
Percent similarity: 49.68

Results for OsAGO11 vs HvAGO5B :
Alignment length: 1003
Identical residues: 399
Similar residues: 109
Percent identity: 39.78
Percent similarity: 50.65

Results for OsAGO11 vs HvAGO6 :
Alignment length: 1038
Identical residues: 251
Similar residues: 112
Percent identity: 24.18
Percent similarity: 34.97

Results for OsAGO11 vs HvAGO2 :
Alignment length: 1128
Identical residues: 251
Similar residues: 127
Percent identity: 22.25
Percent similarity: 33.51

Results for OsAGO11 vs HvAGO18 :
Alignment length: 1107
Identical residues: 356
Similar residues: 119
Percent identity: 32.16
Percent similarity: 42.91

Results for OsAGO11 vs HvAGO5A :
Alignment length: 1045
Identical residues: 585
Similar residues: 101
Percent identity: 55.98
Percent similarity: 65.65

Results for OsAGO11 vs HvAGO10 :
Alignment length: 1039
Identical residues: 399
Similar residues: 127
Percent identity: 38.40
Percent similarity: 50.63

Results for OsAGO11 vs HvAGO1A :
Alignment length: 1242
Identical residues: 400
Similar residues: 122
Percent identity: 32.21
Percent similarity: 42.03

Results for OsAGO12 vs OsAGO13 :
Alignment length: 1443
Identical residues: 479
Similar residues: 103
Percent identity: 33.19
Percent similarity: 40.33

Results for OsAGO12 vs OsAGO14 :
Alignment length: 1087
Identical residues: 538
Similar residues: 135
Percent identity: 49.49
Percent similarity: 61.91

Results for OsAGO12 vs OsAGO15 :
Alignment length: 1117
Identical residues: 271
Similar residues: 149
Percent identity: 24.26
Percent similarity: 37.60

Results for OsAGO12 vs OsAGO16 :
Alignment length: 1112
Identical residues: 291
Similar residues: 133
Percent identity: 26.17
Percent similarity: 38.13

Results for OsAGO12 vs OsAGO17 :
Alignment length: 1074
Identical residues: 357
Similar residues: 132
Percent identity: 33.24
Percent similarity: 45.53

Results for OsAGO12 vs OsAGO18 :
Alignment length: 1139
Identical residues: 405
Similar residues: 154
Percent identity: 35.56
Percent similarity: 49.08

Results for OsAGO12 vs OsMEL1 :
Alignment length: 1071
Identical residues: 586
Similar residues: 126
Percent identity: 54.72
Percent similarity: 66.48

Results for OsAGO12 vs OsPNH1 :
Alignment length: 1080
Identical residues: 506
Similar residues: 157
Percent identity: 46.85
Percent similarity: 61.39

Results for OsAGO12 vs AtAGO1 :
Alignment length: 1093
Identical residues: 515
Similar residues: 151
Percent identity: 47.12
Percent similarity: 60.93

Results for OsAGO12 vs AtAGO2 :
Alignment length: 1140
Identical residues: 288
Similar residues: 153
Percent identity: 25.26
Percent similarity: 38.68

Results for OsAGO12 vs AtAGO3 :
Alignment length: 1267
Identical residues: 298
Similar residues: 157
Percent identity: 23.52
Percent similarity: 35.91

Results for OsAGO12 vs AtAGO4 :
Alignment length: 1118
Identical residues: 289
Similar residues: 158
Percent identity: 25.85
Percent similarity: 39.98

Results for OsAGO12 vs AtAGO5 :
Alignment length: 1071
Identical residues: 546
Similar residues: 129
Percent identity: 50.98
Percent similarity: 63.03

Results for OsAGO12 vs AtAGO6 :
Alignment length: 1105
Identical residues: 290
Similar residues: 125
Percent identity: 26.24
Percent similarity: 37.56

Results for OsAGO12 vs AtAGO7 :
Alignment length: 1125
Identical residues: 310
Similar residues: 152
Percent identity: 27.56
Percent similarity: 41.07

Results for OsAGO12 vs AtAGO8 :
Alignment length: 1099
Identical residues: 268
Similar residues: 139
Percent identity: 24.39
Percent similarity: 37.03

Results for OsAGO12 vs AtAGO9 :
Alignment length: 1110
Identical residues: 288
Similar residues: 156
Percent identity: 25.95
Percent similarity: 40.00

Results for OsAGO12 vs AtAGO10 :
Alignment length: 1088
Identical residues: 468
Similar residues: 154
Percent identity: 43.01
Percent similarity: 57.17

Results for OsAGO12 vs HvAGO4A :
Alignment length: 1117
Identical residues: 292
Similar residues: 155

Percent identity: 26.14
Percent similarity: 40.02

Results for OsAGO12 vs HvAGO4B :
Alignment length: 1112
Identical residues: 259
Similar residues: 129
Percent identity: 23.29
Percent similarity: 34.89

Results for OsAGO12 vs HvAGO7 :
Alignment length: 1126
Identical residues: 322
Similar residues: 156
Percent identity: 28.60
Percent similarity: 42.45

Results for OsAGO12 vs HvAGO1D :
Alignment length: 1133
Identical residues: 503
Similar residues: 151
Percent identity: 44.40
Percent similarity: 57.72

Results for OsAGO12 vs HvAGO5B :
Alignment length: 1068
Identical residues: 499
Similar residues: 113
Percent identity: 46.72
Percent similarity: 57.30

Results for OsAGO12 vs HvAGO6 :
Alignment length: 1112
Identical residues: 288
Similar residues: 141
Percent identity: 25.90
Percent similarity: 38.58

Results for OsAGO12 vs HvAGO2 :
Alignment length: 1146
Identical residues: 307
Similar residues: 147
Percent identity: 26.79
Percent similarity: 39.62

Results for OsAGO12 vs HvAGO18 :
Alignment length: 1109
Identical residues: 427
Similar residues: 150
Percent identity: 38.50
Percent similarity: 52.03

Results for OsAGO12 vs HvAGO5A :
Alignment length: 1061
Identical residues: 693
Similar residues: 110
Percent identity: 65.32
Percent similarity: 75.68

Results for OsAGO12 vs HvAGO10 :
Alignment length: 1081
Identical residues: 454
Similar residues: 157
Percent identity: 42.00
Percent similarity: 56.52

Results for OsAGO12 vs HvAGO1A :
Alignment length: 1248
Identical residues: 487
Similar residues: 150
Percent identity: 39.02
Percent similarity: 51.04

Results for OsAGO13 vs OsAGO14 :
Alignment length: 1450
Identical residues: 471
Similar residues: 108
Percent identity: 32.48
Percent similarity: 39.93

Results for OsAGO13 vs OsAGO15 :
Alignment length: 1333
Identical residues: 249
Similar residues: 136
Percent identity: 18.68
Percent similarity: 28.88

Results for OsAGO13 vs OsAGO16 :
Alignment length: 1318
Identical residues: 258
Similar residues: 133
Percent identity: 19.58
Percent similarity: 29.67

Results for OsAGO13 vs OsAGO17 :
Alignment length: 1299
Identical residues: 329
Similar residues: 123
Percent identity: 25.33
Percent similarity: 34.80

Results for OsAGO13 vs OsAGO18 :
Alignment length: 1511
Identical residues: 315
Similar residues: 135
Percent identity: 20.85
Percent similarity: 29.78

Results for OsAGO13 vs OsMEL1 :
Alignment length: 1438
Identical residues: 724
Similar residues: 29
Percent identity: 50.35
Percent similarity: 52.36

Results for OsAGO13 vs OsPNH1 :
Alignment length: 1373
Identical residues: 436
Similar residues: 116
Percent identity: 31.76
Percent similarity: 40.20

Results for OsAGO13 vs AtAGO1 :
Alignment length: 1430
Identical residues: 459
Similar residues: 113
Percent identity: 32.10
Percent similarity: 40.00

Results for OsAGO13 vs AtAGO2 :
Alignment length: 1455
Identical residues: 226
Similar residues: 147
Percent identity: 15.53
Percent similarity: 25.64

Results for OsAGO13 vs AtAGO3 :
Alignment length: 1627
Identical residues: 218
Similar residues: 144
Percent identity: 13.40
Percent similarity: 22.25

Results for OsAGO13 vs AtAGO4 :
Alignment length: 1357
Identical residues: 251
Similar residues: 138
Percent identity: 18.50
Percent similarity: 28.67

Results for OsAGO13 vs AtAGO5 :
Alignment length: 1393
Identical residues: 458
Similar residues: 116
Percent identity: 32.88
Percent similarity: 41.21

Results for OsAGO13 vs AtAGO6 :
Alignment length: 1324
Identical residues: 248
Similar residues: 123
Percent identity: 18.73
Percent similarity: 28.02

Results for OsAGO13 vs AtAGO7 :
Alignment length: 1449
Identical residues: 258
Similar residues: 130
Percent identity: 17.81
Percent similarity: 26.78

Results for OsAGO13 vs AtAGO8 :
Alignment length: 1310
Identical residues: 245
Similar residues: 123
Percent identity: 18.70
Percent similarity: 28.09

Results for OsAGO13 vs AtAGO9 :
Alignment length: 1326
Identical residues: 258
Similar residues: 146
Percent identity: 19.46
Percent similarity: 30.47

Results for OsAGO13 vs AtAGO10 :
Alignment length: 1375
Identical residues: 424
Similar residues: 132
Percent identity: 30.84
Percent similarity: 40.44

Results for OsAGO13 vs HvAGO4A :
Alignment length: 1356
Identical residues: 265
Similar residues: 138
Percent identity: 19.54
Percent similarity: 29.72

Results for OsAGO13 vs HvAGO4B :

Alignment length: 1287
Identical residues: 248
Similar residues: 122
Percent identity: 19.27
Percent similarity: 28.75

Results for OsAGO13 vs HvAGO7 :

Alignment length: 1448
Identical residues: 263
Similar residues: 134
Percent identity: 18.16
Percent similarity: 27.42

Results for OsAGO13 vs HvAGO1D :

Alignment length: 1446
Identical residues: 448
Similar residues: 119
Percent identity: 30.98
Percent similarity: 39.21

Results for OsAGO13 vs HvAGO5B :

Alignment length: 1261
Identical residues: 499
Similar residues: 92
Percent identity: 39.57
Percent similarity: 46.87

Results for OsAGO13 vs HvAGO6 :

Alignment length: 1318
Identical residues: 264
Similar residues: 132
Percent identity: 20.03
Percent similarity: 30.05

Results for OsAGO13 vs HvAGO2 :

Alignment length: 1493
Identical residues: 236
Similar residues: 118
Percent identity: 15.81
Percent similarity: 23.71

Results for OsAGO13 vs HvAGO18 :

Alignment length: 1475
Identical residues: 361
Similar residues: 123
Percent identity: 24.47
Percent similarity: 32.81

Results for OsAGO13 vs HvAGO5A :

Alignment length: 1427
Identical residues: 452
Similar residues: 125
Percent identity: 31.67
Percent similarity: 40.43

Results for OsAGO13 vs HvAGO10 :

Alignment length: 1347
Identical residues: 406
Similar residues: 122
Percent identity: 30.14
Percent similarity: 39.20

Results for OsAGO13 vs HvAGO1A :

Alignment length: 1595
Identical residues: 422
Similar residues: 116
Percent identity: 26.46
Percent similarity: 33.73

Results for OsAGO14 vs OsAGO15 :

Alignment length: 1134
Identical residues: 281
Similar residues: 127
Percent identity: 24.78
Percent similarity: 35.98

Results for OsAGO14 vs OsAGO16 :

Alignment length: 1127
Identical residues: 284
Similar residues: 124
Percent identity: 25.20
Percent similarity: 36.20

Results for OsAGO14 vs OsAGO17 :

Alignment length: 1091
Identical residues: 357
Similar residues: 138
Percent identity: 32.72
Percent similarity: 45.37

Results for OsAGO14 vs OsAGO18 :

Alignment length: 1149
Identical residues: 396
Similar residues: 150
Percent identity: 34.46
Percent similarity: 47.52

Results for OsAGO14 vs OsMEL1 :

Alignment length: 1082
Identical residues: 597
Similar residues: 119
Percent identity: 55.18
Percent similarity: 66.17

Results for OsAGO14 vs OsPNH1 :

Alignment length: 1089
Identical residues: 495
Similar residues: 146
Percent identity: 45.45
Percent similarity: 58.86

Results for OsAGO14 vs AtAGO1 :

Alignment length: 1106
Identical residues: 502
Similar residues: 146
Percent identity: 45.39
Percent similarity: 58.59

Results for OsAGO14 vs AtAGO2 :

Alignment length: 1154
Identical residues: 282
Similar residues: 167
Percent identity: 24.44
Percent similarity: 38.91

Results for OsAGO14 vs AtAGO3 :

Alignment length: 1278
Identical residues: 290
Similar residues: 171
Percent identity: 22.69
Percent similarity: 36.07

Results for OsAGO14 vs AtAGO4 :

Alignment length: 1134
Identical residues: 299
Similar residues: 141
Percent identity: 26.37
Percent similarity: 38.80

Results for OsAGO14 vs AtAGO5 :

Alignment length: 1074
Identical residues: 509
Similar residues: 139
Percent identity: 47.39
Percent similarity: 60.34

Results for OsAGO14 vs AtAGO6 :

Alignment length: 1122
Identical residues: 287
Similar residues: 125
Percent identity: 25.58
Percent similarity: 36.72

Results for OsAGO14 vs AtAGO7 :

Alignment length: 1135
Identical residues: 295
Similar residues: 144
Percent identity: 25.99
Percent similarity: 38.68

Results for OsAGO14 vs AtAGO8 :

Alignment length: 1115
Identical residues: 269
Similar residues: 117
Percent identity: 24.13
Percent similarity: 34.62

Results for OsAGO14 vs AtAGO9 :

Alignment length: 1125
Identical residues: 287
Similar residues: 143
Percent identity: 25.51
Percent similarity: 38.22

Results for OsAGO14 vs AtAGO10 :

Alignment length: 1097
Identical residues: 467
Similar residues: 144
Percent identity: 42.57
Percent similarity: 55.70

Results for OsAGO14 vs HvAGO4A :

Alignment length: 1132
Identical residues: 288
Similar residues: 147
Percent identity: 25.44
Percent similarity: 38.43

Results for OsAGO14 vs HvAGO4B :

Alignment length: 1134
Identical residues: 261
Similar residues: 121
Percent identity: 23.02
Percent similarity: 33.69

Results for OsAGO14 vs HvAGO7 :

Alignment length: 1134
Identical residues: 305
Similar residues: 159
Percent identity: 26.90
Percent similarity: 40.92

Results for OsAGO14 vs HvAGO1D :
Alignment length: 1146
Identical residues: 487
Similar residues: 144
Percent identity: 42.50
Percent similarity: 55.06

Results for OsAGO14 vs HvAGO5B :
Alignment length: 1065
Identical residues: 570
Similar residues: 93
Percent identity: 53.52
Percent similarity: 62.25

Results for OsAGO14 vs HvAGO6 :
Alignment length: 1127
Identical residues: 289
Similar residues: 127
Percent identity: 25.64
Percent similarity: 36.91

Results for OsAGO14 vs HvAGO2 :
Alignment length: 1158
Identical residues: 305
Similar residues: 147
Percent identity: 26.34
Percent similarity: 39.03

Results for OsAGO14 vs HvAGO18 :
Alignment length: 1122
Identical residues: 430
Similar residues: 141
Percent identity: 38.32
Percent similarity: 50.89

Results for OsAGO14 vs HvAGO5A :
Alignment length: 1083
Identical residues: 510
Similar residues: 147
Percent identity: 47.09
Percent similarity: 60.66

Results for OsAGO14 vs HvAGO10 :
Alignment length: 1088
Identical residues: 464
Similar residues: 136
Percent identity: 42.65
Percent similarity: 55.15

Results for OsAGO14 vs HvAGO1A :
Alignment length: 1264
Identical residues: 488
Similar residues: 142
Percent identity: 38.61
Percent similarity: 49.84

Results for OsAGO15 vs OsAGO16 :
Alignment length: 913
Identical residues: 460
Similar residues: 120
Percent identity: 50.38
Percent similarity: 63.53

Results for OsAGO15 vs OsAGO17 :
Alignment length: 971
Identical residues: 245
Similar residues: 151
Percent identity: 25.23
Percent similarity: 40.78

Results for OsAGO15 vs OsAGO18 :
Alignment length: 1170
Identical residues: 267
Similar residues: 145
Percent identity: 22.82
Percent similarity: 35.21

Results for OsAGO15 vs OsMEL1 :
Alignment length: 1123
Identical residues: 284
Similar residues: 140
Percent identity: 25.29
Percent similarity: 37.76

Results for OsAGO15 vs OsPNH1 :
Alignment length: 1042
Identical residues: 293
Similar residues: 137
Percent identity: 28.12
Percent similarity: 41.27

Results for OsAGO15 vs AtAGO1 :
Alignment length: 1111
Identical residues: 299
Similar residues: 122
Percent identity: 26.91
Percent similarity: 37.89

Results for OsAGO15 vs AtAGO2 :
Alignment length: 1101
Identical residues: 226
Similar residues: 132
Percent identity: 20.53
Percent similarity: 32.52

Results for OsAGO15 vs AtAGO3 :
Alignment length: 1273
Identical residues: 225
Similar residues: 138
Percent identity: 17.67
Percent similarity: 28.52

Results for OsAGO15 vs AtAGO4 :
Alignment length: 941
Identical residues: 511
Similar residues: 121
Percent identity: 54.30
Percent similarity: 67.16

Results for OsAGO15 vs AtAGO5 :
Alignment length: 1073
Identical residues: 272
Similar residues: 131
Percent identity: 25.35
Percent similarity: 37.56

Results for OsAGO15 vs AtAGO6 :
Alignment length: 918
Identical residues: 442
Similar residues: 100
Percent identity: 48.15
Percent similarity: 59.04

Results for OsAGO15 vs AtAGO7 :
Alignment length: 1078
Identical residues: 244
Similar residues: 137
Percent identity: 22.63
Percent similarity: 35.34

Results for OsAGO15 vs AtAGO8 :
Alignment length: 918
Identical residues: 436
Similar residues: 113
Percent identity: 47.49
Percent similarity: 59.80

Results for OsAGO15 vs AtAGO9 :
Alignment length: 924
Identical residues: 489
Similar residues: 114
Percent identity: 52.92
Percent similarity: 65.26

Results for OsAGO15 vs AtAGO10 :
Alignment length: 1045
Identical residues: 286
Similar residues: 137
Percent identity: 27.37
Percent similarity: 40.48

Results for OsAGO15 vs HvAGO4A :
Alignment length: 941
Identical residues: 551
Similar residues: 108
Percent identity: 58.55
Percent similarity: 70.03

Results for OsAGO15 vs HvAGO4B :
Alignment length: 912
Identical residues: 481
Similar residues: 87
Percent identity: 52.74
Percent similarity: 62.28

Results for OsAGO15 vs HvAGO7 :
Alignment length: 1085
Identical residues: 237
Similar residues: 153
Percent identity: 21.84
Percent similarity: 35.94

Results for OsAGO15 vs HvAGO1D :
Alignment length: 1123
Identical residues: 289
Similar residues: 136
Percent identity: 25.73
Percent similarity: 37.85

Results for OsAGO15 vs HvAGO5B :
Alignment length: 950
Identical residues: 264

Similar residues: 145
Percent identity: 27.79
Percent similarity: 43.05

Results for OsAGO15 vs HvAGO6 :
Alignment length: 913
Identical residues: 453
Similar residues: 127
Percent identity: 49.62
Percent similarity: 63.53

Results for OsAGO15 vs HvAGO2 :
Alignment length: 1143
Identical residues: 235
Similar residues: 134
Percent identity: 20.56
Percent similarity: 32.28

Results for OsAGO15 vs HvAGO18 :
Alignment length: 1133
Identical residues: 293
Similar residues: 138
Percent identity: 25.86
Percent similarity: 38.04

Results for OsAGO15 vs HvAGO5A :
Alignment length: 1110
Identical residues: 292
Similar residues: 129
Percent identity: 26.31
Percent similarity: 37.93

Results for OsAGO15 vs HvAGO10 :
Alignment length: 1017
Identical residues: 278
Similar residues: 132
Percent identity: 27.34
Percent similarity: 40.31

Results for OsAGO15 vs HvAGO1A :
Alignment length: 1272
Identical residues: 279
Similar residues: 128
Percent identity: 21.93
Percent similarity: 32.00

Results for OsAGO16 vs OsAGO17 :
Alignment length: 964
Identical residues: 253
Similar residues: 144
Percent identity: 26.24
Percent similarity: 41.18

Results for OsAGO16 vs OsAGO18 :
Alignment length: 1165
Identical residues: 266
Similar residues: 142
Percent identity: 22.83
Percent similarity: 35.02

Results for OsAGO16 vs OsMEL1 :
Alignment length: 1115
Identical residues: 296
Similar residues: 148
Percent identity: 26.55
Percent similarity: 39.82

Results for OsAGO16 vs OsPNH1 :
Alignment length: 1035
Identical residues: 301
Similar residues: 135
Percent identity: 29.08
Percent similarity: 42.13

Results for OsAGO16 vs AtAGO1 :
Alignment length: 1105
Identical residues: 300
Similar residues: 138
Percent identity: 27.15
Percent similarity: 39.64

Results for OsAGO16 vs AtAGO2 :
Alignment length: 1095
Identical residues: 234
Similar residues: 125
Percent identity: 21.37
Percent similarity: 32.79

Results for OsAGO16 vs AtAGO3 :
Alignment length: 1265
Identical residues: 241
Similar residues: 127
Percent identity: 19.05
Percent similarity: 29.09

Results for OsAGO16 vs AtAGO4 :
Alignment length: 926
Identical residues: 510
Similar residues: 112
Percent identity: 55.08
Percent similarity: 67.17

Results for OsAGO16 vs AtAGO5 :
Alignment length: 1060
Identical residues: 296
Similar residues: 124
Percent identity: 27.92
Percent similarity: 39.62

Results for OsAGO16 vs AtAGO6 :
Alignment length: 893
Identical residues: 528
Similar residues: 93
Percent identity: 59.13
Percent similarity: 69.54

Results for OsAGO16 vs AtAGO7 :
Alignment length: 1070
Identical residues: 247
Similar residues: 146
Percent identity: 23.08
Percent similarity: 36.73

Results for OsAGO16 vs AtAGO8 :
Alignment length: 896
Identical residues: 455
Similar residues: 108
Percent identity: 50.78
Percent similarity: 62.83

Results for OsAGO16 vs AtAGO9 :
Alignment length: 904
Identical residues: 511
Similar residues: 109
Percent identity: 56.53
Percent similarity: 68.58

Results for OsAGO16 vs AtAGO10 :
Alignment length: 1038
Identical residues: 290
Similar residues: 133
Percent identity: 27.94
Percent similarity: 40.75

Results for OsAGO16 vs HvAGO4A :
Alignment length: 927
Identical residues: 525
Similar residues: 100
Percent identity: 56.63
Percent similarity: 67.42

Results for OsAGO16 vs HvAGO4B :
Alignment length: 910
Identical residues: 424
Similar residues: 105
Percent identity: 46.59
Percent similarity: 58.13

Results for OsAGO16 vs HvAGO7 :
Alignment length: 1077
Identical residues: 242
Similar residues: 155
Percent identity: 22.47
Percent similarity: 36.86

Results for OsAGO16 vs HvAGO1D :
Alignment length: 1115
Identical residues: 295
Similar residues: 139
Percent identity: 26.46
Percent similarity: 38.92

Results for OsAGO16 vs HvAGO5B :
Alignment length: 943
Identical residues: 272
Similar residues: 135
Percent identity: 28.84
Percent similarity: 43.16

Results for OsAGO16 vs HvAGO6 :
Alignment length: 883
Identical residues: 758
Similar residues: 50
Percent identity: 85.84
Percent similarity: 91.51

Results for OsAGO16 vs HvAGO2 :
Alignment length: 1137
Identical residues: 245
Similar residues: 122
Percent identity: 21.55
Percent similarity: 32.28

Results for OsAGO16 vs HvAGO18 :
Alignment length: 1128
Identical residues: 279
Similar residues: 133
Percent identity: 24.73

Percent similarity: 36.52

Results for OsAGO16 vs HvAGO5A :

Alignment length: 1098
Identical residues: 296
Similar residues: 125
Percent identity: 26.96
Percent similarity: 38.34

Results for OsAGO16 vs HvAGO10 :

Alignment length: 1010
Identical residues: 298
Similar residues: 128
Percent identity: 29.50
Percent similarity: 42.18

Results for OsAGO16 vs HvAGO1A :

Alignment length: 1264
Identical residues: 280
Similar residues: 142
Percent identity: 22.15
Percent similarity: 33.39

Results for OsAGO17 vs OsAGO18 :

Alignment length: 1143
Identical residues: 328
Similar residues: 136
Percent identity: 28.70
Percent similarity: 40.59

Results for OsAGO17 vs OsMEL1 :

Alignment length: 1079
Identical residues: 378
Similar residues: 134
Percent identity: 35.03
Percent similarity: 47.45

Results for OsAGO17 vs OsPNH1 :

Alignment length: 991
Identical residues: 441
Similar residues: 131
Percent identity: 44.50
Percent similarity: 57.72

Results for OsAGO17 vs AtAGO1 :

Alignment length: 1059
Identical residues: 446
Similar residues: 116
Percent identity: 42.12
Percent similarity: 53.07

Results for OsAGO17 vs AtAGO2 :

Alignment length: 1083
Identical residues: 218
Similar residues: 147
Percent identity: 20.13
Percent similarity: 33.70

Results for OsAGO17 vs AtAGO3 :

Alignment length: 1260
Identical residues: 215
Similar residues: 141
Percent identity: 17.06
Percent similarity: 28.25

Results for OsAGO17 vs AtAGO4 :

Alignment length: 981
Identical residues: 254
Similar residues: 149
Percent identity: 25.89
Percent similarity: 41.08

Results for OsAGO17 vs AtAGO5 :

Alignment length: 1032
Identical residues: 370
Similar residues: 128
Percent identity: 35.85
Percent similarity: 48.26

Results for OsAGO17 vs AtAGO6 :

Alignment length: 965
Identical residues: 245
Similar residues: 132
Percent identity: 25.39
Percent similarity: 39.07

Results for OsAGO17 vs AtAGO7 :

Alignment length: 1065
Identical residues: 262
Similar residues: 113
Percent identity: 24.60
Percent similarity: 35.21

Results for OsAGO17 vs AtAGO8 :

Alignment length: 955
Identical residues: 233
Similar residues: 121
Percent identity: 24.40
Percent similarity: 37.07

Results for OsAGO17 vs AtAGO9 :

Alignment length: 974
Identical residues: 255
Similar residues: 141
Percent identity: 26.18
Percent similarity: 40.66

Results for OsAGO17 vs AtAGO10 :

Alignment length: 995
Identical residues: 437
Similar residues: 130
Percent identity: 43.92
Percent similarity: 56.98

Results for OsAGO17 vs HvAGO4A :

Alignment length: 990
Identical residues: 250
Similar residues: 148
Percent identity: 25.25
Percent similarity: 40.20

Results for OsAGO17 vs HvAGO4B :

Alignment length: 955
Identical residues: 226
Similar residues: 137
Percent identity: 23.66
Percent similarity: 38.01

Results for OsAGO17 vs HvAGO7 :

Alignment length: 1064
Identical residues: 249
Similar residues: 136
Percent identity: 23.40
Percent similarity: 36.18

Results for OsAGO17 vs HvAGO1D :

Alignment length: 1075
Identical residues: 436
Similar residues: 124
Percent identity: 40.56
Percent similarity: 52.09

Results for OsAGO17 vs HvAGO5B :

Alignment length: 921
Identical residues: 361
Similar residues: 134
Percent identity: 39.20
Percent similarity: 53.75

Results for OsAGO17 vs HvAGO6 :

Alignment length: 964
Identical residues: 251
Similar residues: 143
Percent identity: 26.04
Percent similarity: 40.87

Results for OsAGO17 vs HvAGO2 :

Alignment length: 1124
Identical residues: 220
Similar residues: 132
Percent identity: 19.57
Percent similarity: 31.32

Results for OsAGO17 vs HvAGO18 :

Alignment length: 1104
Identical residues: 347
Similar residues: 141
Percent identity: 31.43
Percent similarity: 44.20

Results for OsAGO17 vs HvAGO5A :

Alignment length: 1058
Identical residues: 349
Similar residues: 149
Percent identity: 32.99
Percent similarity: 47.07

Results for OsAGO17 vs HvAGO10 :

Alignment length: 967
Identical residues: 427
Similar residues: 131
Percent identity: 44.16
Percent similarity: 57.70

Results for OsAGO17 vs HvAGO1A :

Alignment length: 1222
Identical residues: 443
Similar residues: 121
Percent identity: 36.25
Percent similarity: 46.15

Results for OsAGO18 vs OsMEL1 :

Alignment length: 1143
Identical residues: 400
Similar residues: 151
Percent identity: 35.00
Percent similarity: 48.21

Results for OsAGO18 vs OsPNH1 :

Alignment length: 1135

Identical residues: 432

Similar residues: 140

Percent identity: 38.06

Percent similarity: 50.40

Results for OsAGO18 vs AtAGO1 :

Alignment length: 1163

Identical residues: 449

Similar residues: 135

Percent identity: 38.61

Percent similarity: 50.21

Results for OsAGO18 vs AtAGO2 :

Alignment length: 1164

Identical residues: 277

Similar residues: 164

Percent identity: 23.80

Percent similarity: 37.89

Results for OsAGO18 vs AtAGO3 :

Alignment length: 1271

Identical residues: 303

Similar residues: 157

Percent identity: 23.84

Percent similarity: 36.19

Results for OsAGO18 vs AtAGO4 :

Alignment length: 1177

Identical residues: 272

Similar residues: 155

Percent identity: 23.11

Percent similarity: 36.28

Results for OsAGO18 vs AtAGO5 :

Alignment length: 1139

Identical residues: 400

Similar residues: 135

Percent identity: 35.12

Percent similarity: 46.97

Results for OsAGO18 vs AtAGO6 :

Alignment length: 1162

Identical residues: 272

Similar residues: 139

Percent identity: 23.41

Percent similarity: 35.37

Results for OsAGO18 vs AtAGO7 :

Alignment length: 1165

Identical residues: 288

Similar residues: 147

Percent identity: 24.72

Percent similarity: 37.34

Results for OsAGO18 vs AtAGO8 :

Alignment length: 1152

Identical residues: 258

Similar residues: 127

Percent identity: 22.40

Percent similarity: 33.42

Results for OsAGO18 vs AtAGO9 :

Alignment length: 1162

Identical residues: 266

Similar residues: 163

Percent identity: 22.89

Percent similarity: 36.92

Results for OsAGO18 vs AtAGO10 :

Alignment length: 1140

Identical residues: 418

Similar residues: 131

Percent identity: 36.67

Percent similarity: 48.16

Results for OsAGO18 vs HvAGO4A :

Alignment length: 1169

Identical residues: 268

Similar residues: 161

Percent identity: 22.93

Percent similarity: 36.70

Results for OsAGO18 vs HvAGO4B :

Alignment length: 1171

Identical residues: 244

Similar residues: 144

Percent identity: 20.84

Percent similarity: 33.13

Results for OsAGO18 vs HvAGO7 :

Alignment length: 1162

Identical residues: 290

Similar residues: 152

Percent identity: 24.96

Percent similarity: 38.04

Results for OsAGO18 vs HvAGO1D :

Alignment length: 1164

Identical residues: 436

Similar residues: 159

Percent identity: 37.46

Percent similarity: 51.12

Results for OsAGO18 vs HvAGO5B :

Alignment length: 1132

Identical residues: 360

Similar residues: 130

Percent identity: 31.80

Percent similarity: 43.29

Results for OsAGO18 vs HvAGO6 :

Alignment length: 1165

Identical residues: 277

Similar residues: 133

Percent identity: 23.78

Percent similarity: 35.19

Results for OsAGO18 vs HvAGO2 :

Alignment length: 1158

Identical residues: 297

Similar residues: 155

Percent identity: 25.65

Percent similarity: 39.03

Results for OsAGO18 vs HvAGO18 :

Alignment length: 1109

Identical residues: 586

Similar residues: 116

Percent identity: 52.84

Percent similarity: 63.30

Results for OsAGO18 vs HvAGO5A :

Alignment length: 1141

Identical residues: 413

Similar residues: 158

Percent identity: 36.20

Percent similarity: 50.04

Results for OsAGO18 vs HvAGO10 :

Alignment length: 1134

Identical residues: 407

Similar residues: 149

Percent identity: 35.89

Percent similarity: 49.03

Results for OsAGO18 vs HvAGO1A :

Alignment length: 1304

Identical residues: 428

Similar residues: 137

Percent identity: 32.82

Percent similarity: 43.33

Results for OsMEL1 vs OsPNH1 :

Alignment length: 1076

Identical residues: 520

Similar residues: 132

Percent identity: 48.33

Percent similarity: 60.59

Results for OsMEL1 vs AtAGO1 :

Alignment length: 1095

Identical residues: 547

Similar residues: 132

Percent identity: 49.95

Percent similarity: 62.01

Results for OsMEL1 vs AtAGO2 :

Alignment length: 1137

Identical residues: 287

Similar residues: 164

Percent identity: 25.24

Percent similarity: 39.67

Results for OsMEL1 vs AtAGO3 :

Alignment length: 1260

Identical residues: 283

Similar residues: 166

Percent identity: 22.46

Percent similarity: 35.63

Results for OsMEL1 vs AtAGO4 :

Alignment length: 1121

Identical residues: 302

Similar residues: 152

Percent identity: 26.94

Percent similarity: 40.50

Results for OsMEL1 vs AtAGO5 :

Alignment length: 1067

Identical residues: 561

Similar residues: 133

Percent identity: 52.58

Percent similarity: 65.04

Results for OsMEL1 vs AtAGO6 :

Alignment length: 1111

Identical residues: 289
Similar residues: 139
Percent identity: 26.01
Percent similarity: 38.52

Results for OsMEL1 vs AtAGO7 :
Alignment length: 1124
Identical residues: 314
Similar residues: 148
Percent identity: 27.94
Percent similarity: 41.10

Results for OsMEL1 vs AtAGO8 :
Alignment length: 1102
Identical residues: 283
Similar residues: 124
Percent identity: 25.68
Percent similarity: 36.93

Results for OsMEL1 vs AtAGO9 :
Alignment length: 1113
Identical residues: 300
Similar residues: 150
Percent identity: 26.95
Percent similarity: 40.43

Results for OsMEL1 vs AtAGO10 :
Alignment length: 1084
Identical residues: 500
Similar residues: 149
Percent identity: 46.13
Percent similarity: 59.87

Results for OsMEL1 vs HvAGO4A :
Alignment length: 1119
Identical residues: 301
Similar residues: 152
Percent identity: 26.90
Percent similarity: 40.48

Results for OsMEL1 vs HvAGO4B :
Alignment length: 1120
Identical residues: 277
Similar residues: 125
Percent identity: 24.73
Percent similarity: 35.89

Results for OsMEL1 vs HvAGO7 :
Alignment length: 1123
Identical residues: 326
Similar residues: 162
Percent identity: 29.03
Percent similarity: 43.46

Results for OsMEL1 vs HvAGO1D :
Alignment length: 1130
Identical residues: 533
Similar residues: 138
Percent identity: 47.17
Percent similarity: 59.38

Results for OsMEL1 vs HvAGO5B :
Alignment length: 1071
Identical residues: 576
Similar residues: 94
Percent identity: 53.78
Percent similarity: 62.56

Results for OsMEL1 vs HvAGO6 :
Alignment length: 1115
Identical residues: 303
Similar residues: 145
Percent identity: 27.17
Percent similarity: 40.18

Results for OsMEL1 vs HvAGO2 :
Alignment length: 1143
Identical residues: 302
Similar residues: 150
Percent identity: 26.42
Percent similarity: 39.55

Results for OsMEL1 vs HvAGO18 :
Alignment length: 1112
Identical residues: 443
Similar residues: 148
Percent identity: 39.84
Percent similarity: 53.15

Results for OsMEL1 vs HvAGO5A :
Alignment length: 1071
Identical residues: 549
Similar residues: 152
Percent identity: 51.26
Percent similarity: 65.45

Results for OsMEL1 vs HvAGO10 :
Alignment length: 1075
Identical residues: 491
Similar residues: 139
Percent identity: 45.67
Percent similarity: 58.60

Results for OsMEL1 vs HvAGO1A :
Alignment length: 1246
Identical residues: 517
Similar residues: 136
Percent identity: 41.49
Percent similarity: 52.41

Results for OsPNH1 vs AtAGO1 :
Alignment length: 1067
Identical residues: 665
Similar residues: 89
Percent identity: 62.32
Percent similarity: 70.67

Results for OsPNH1 vs AtAGO2 :
Alignment length: 1111
Identical residues: 285
Similar residues: 167
Percent identity: 25.65
Percent similarity: 40.68

Results for OsPNH1 vs AtAGO3 :
Alignment length: 1255
Identical residues: 293
Similar residues: 158
Percent identity: 23.35
Percent similarity: 35.94

Results for OsPNH1 vs AtAGO4 :
Alignment length: 1043
Identical residues: 301
Similar residues: 163
Percent identity: 28.86
Percent similarity: 44.49

Results for OsPNH1 vs AtAGO5 :
Alignment length: 1043
Identical residues: 499
Similar residues: 138
Percent identity: 47.84
Percent similarity: 61.07

Results for OsPNH1 vs AtAGO6 :
Alignment length: 1033
Identical residues: 302
Similar residues: 127
Percent identity: 29.24
Percent similarity: 41.53

Results for OsPNH1 vs AtAGO7 :
Alignment length: 1108
Identical residues: 316
Similar residues: 138
Percent identity: 28.52
Percent similarity: 40.97

Results for OsPNH1 vs AtAGO8 :
Alignment length: 1023
Identical residues: 275
Similar residues: 133
Percent identity: 26.88
Percent similarity: 39.88

Results for OsPNH1 vs AtAGO9 :
Alignment length: 1034
Identical residues: 290
Similar residues: 163
Percent identity: 28.05
Percent similarity: 43.81

Results for OsPNH1 vs AtAGO10 :
Alignment length: 991
Identical residues: 721
Similar residues: 98
Percent identity: 72.75
Percent similarity: 82.64

Results for OsPNH1 vs HvAGO4A :
Alignment length: 1053
Identical residues: 301
Similar residues: 147
Percent identity: 28.58
Percent similarity: 42.55

Results for OsPNH1 vs HvAGO4B :
Alignment length: 1039
Identical residues: 272
Similar residues: 126
Percent identity: 26.18
Percent similarity: 38.31

Results for OsPNH1 vs HvAGO7 :
Alignment length: 1100
Identical residues: 314
Similar residues: 148

Percent identity: 28.55
Percent similarity: 42.00

Results for OsPNH1 vs HvAGO1D :
Alignment length: 1075
Identical residues: 630
Similar residues: 115
Percent identity: 58.60
Percent similarity: 69.30

Results for OsPNH1 vs HvAGO5B :
Alignment length: 1002
Identical residues: 478
Similar residues: 121
Percent identity: 47.70
Percent similarity: 59.78

Results for OsPNH1 vs HvAGO6 :
Alignment length: 1035
Identical residues: 298
Similar residues: 139
Percent identity: 28.79
Percent similarity: 42.22

Results for OsPNH1 vs HvAGO2 :
Alignment length: 1136
Identical residues: 280
Similar residues: 153
Percent identity: 24.65
Percent similarity: 38.12

Results for OsPNH1 vs HvAGO18 :
Alignment length: 1101
Identical residues: 470
Similar residues: 136
Percent identity: 42.69
Percent similarity: 55.04

Results for OsPNH1 vs HvAGO5A :
Alignment length: 1069
Identical residues: 479
Similar residues: 162
Percent identity: 44.81
Percent similarity: 59.96

Results for OsPNH1 vs HvAGO10 :
Alignment length: 980
Identical residues: 749
Similar residues: 77
Percent identity: 76.43
Percent similarity: 84.29

Results for OsPNH1 vs HvAGO1A :
Alignment length: 1233
Identical residues: 636
Similar residues: 100
Percent identity: 51.58
Percent similarity: 59.69

Results for AtAGO1 vs AtAGO2 :
Alignment length: 1142
Identical residues: 297
Similar residues: 161
Percent identity: 26.01
Percent similarity: 40.11

Results for AtAGO1 vs AtAGO3 :
Alignment length: 1268
Identical residues: 288
Similar residues: 167
Percent identity: 22.71
Percent similarity: 35.88

Results for AtAGO1 vs AtAGO4 :
Alignment length: 1111
Identical residues: 303
Similar residues: 161
Percent identity: 27.27
Percent similarity: 41.76

Results for AtAGO1 vs AtAGO5 :
Alignment length: 1084
Identical residues: 536
Similar residues: 126
Percent identity: 49.45
Percent similarity: 61.07

Results for AtAGO1 vs AtAGO6 :
Alignment length: 1103
Identical residues: 304
Similar residues: 131
Percent identity: 27.56
Percent similarity: 39.44

Results for AtAGO1 vs AtAGO7 :
Alignment length: 1138
Identical residues: 323
Similar residues: 136
Percent identity: 28.38
Percent similarity: 40.33

Results for AtAGO1 vs AtAGO8 :
Alignment length: 1094
Identical residues: 282
Similar residues: 136
Percent identity: 25.78
Percent similarity: 38.21

Results for AtAGO1 vs AtAGO9 :
Alignment length: 1103
Identical residues: 304
Similar residues: 155
Percent identity: 27.56
Percent similarity: 41.61

Results for AtAGO1 vs AtAGO10 :
Alignment length: 1071
Identical residues: 659
Similar residues: 83
Percent identity: 61.53
Percent similarity: 69.28

Results for AtAGO1 vs HvAGO4A :
Alignment length: 1112
Identical residues: 300
Similar residues: 144
Percent identity: 26.98
Percent similarity: 39.93

Results for AtAGO1 vs HvAGO4B :
Alignment length: 1106
Identical residues: 276
Similar residues: 133
Percent identity: 24.95
Percent similarity: 36.98

Results for AtAGO1 vs HvAGO7 :
Alignment length: 1136
Identical residues: 323
Similar residues: 159
Percent identity: 28.43
Percent similarity: 42.43

Results for AtAGO1 vs HvAGO1D :
Alignment length: 1112
Identical residues: 672
Similar residues: 97
Percent identity: 60.43
Percent similarity: 69.15

Results for AtAGO1 vs HvAGO5B :
Alignment length: 1063
Identical residues: 488
Similar residues: 120
Percent identity: 45.91
Percent similarity: 57.20

Results for AtAGO1 vs HvAGO6 :
Alignment length: 1105
Identical residues: 305
Similar residues: 139
Percent identity: 27.60
Percent similarity: 40.18

Results for AtAGO1 vs HvAGO2 :
Alignment length: 1163
Identical residues: 309
Similar residues: 152
Percent identity: 26.57
Percent similarity: 39.64

Results for AtAGO1 vs HvAGO18 :
Alignment length: 1135
Identical residues: 484
Similar residues: 128
Percent identity: 42.64
Percent similarity: 53.92

Results for AtAGO1 vs HvAGO5A :
Alignment length: 1087
Identical residues: 515
Similar residues: 155
Percent identity: 47.38
Percent similarity: 61.64

Results for AtAGO1 vs HvAGO10 :
Alignment length: 1064
Identical residues: 603
Similar residues: 106
Percent identity: 56.67
Percent similarity: 66.64

Results for AtAGO1 vs HvAGO1A :
Alignment length: 1224
Identical residues: 756
Similar residues: 74
Percent identity: 61.76
Percent similarity: 67.81

Results for AtAGO2 vs AtAGO3 :
Alignment length: 1203
Identical residues: 725
Similar residues: 95
Percent identity: 60.27
Percent similarity: 68.16

Results for AtAGO2 vs AtAGO4 :
Alignment length: 1102
Identical residues: 239
Similar residues: 156
Percent identity: 21.69
Percent similarity: 35.84

Results for AtAGO2 vs AtAGO5 :
Alignment length: 1129
Identical residues: 285
Similar residues: 160
Percent identity: 25.24
Percent similarity: 39.42

Results for AtAGO2 vs AtAGO6 :
Alignment length: 1092
Identical residues: 239
Similar residues: 137
Percent identity: 21.89
Percent similarity: 34.43

Results for AtAGO2 vs AtAGO7 :
Alignment length: 1078
Identical residues: 291
Similar residues: 166
Percent identity: 26.99
Percent similarity: 42.39

Results for AtAGO2 vs AtAGO8 :
Alignment length: 1078
Identical residues: 219
Similar residues: 138
Percent identity: 20.32
Percent similarity: 33.12

Results for AtAGO2 vs AtAGO9 :
Alignment length: 1091
Identical residues: 239
Similar residues: 149
Percent identity: 21.91
Percent similarity: 35.56

Results for AtAGO2 vs AtAGO10 :
Alignment length: 1116
Identical residues: 281
Similar residues: 161
Percent identity: 25.18
Percent similarity: 39.61

Results for AtAGO2 vs HvAGO4A :
Alignment length: 1097
Identical residues: 238
Similar residues: 140
Percent identity: 21.70
Percent similarity: 34.46

Results for AtAGO2 vs HvAGO4B :
Alignment length: 1099
Identical residues: 198
Similar residues: 136
Percent identity: 18.02
Percent similarity: 30.39

Results for AtAGO2 vs HvAGO7 :
Alignment length: 1081
Identical residues: 307
Similar residues: 158
Percent identity: 28.40
Percent similarity: 43.02

Results for AtAGO2 vs HvAGO1D :
Alignment length: 1158
Identical residues: 277
Similar residues: 173
Percent identity: 23.92
Percent similarity: 38.86

Results for AtAGO2 vs HvAGO5B :
Alignment length: 1078
Identical residues: 266
Similar residues: 147
Percent identity: 24.68
Percent similarity: 38.31

Results for AtAGO2 vs HvAGO6 :
Alignment length: 1095
Identical residues: 235
Similar residues: 131
Percent identity: 21.46
Percent similarity: 33.42

Results for AtAGO2 vs HvAGO2 :
Alignment length: 1090
Identical residues: 387
Similar residues: 165
Percent identity: 35.50
Percent similarity: 50.64

Results for AtAGO2 vs HvAGO18 :
Alignment length: 1135
Identical residues: 293
Similar residues: 167
Percent identity: 25.81
Percent similarity: 40.53

Results for AtAGO2 vs HvAGO5A :
Alignment length: 1135
Identical residues: 284
Similar residues: 155
Percent identity: 25.02
Percent similarity: 38.68

Results for AtAGO2 vs HvAGO10 :
Alignment length: 1097
Identical residues: 274
Similar residues: 167
Percent identity: 24.98
Percent similarity: 40.20

Results for AtAGO2 vs HvAGO1A :
Alignment length: 1304
Identical residues: 281
Similar residues: 155
Percent identity: 21.55
Percent similarity: 33.44

Results for AtAGO3 vs AtAGO4 :
Alignment length: 1272
Identical residues: 230
Similar residues: 153
Percent identity: 18.08
Percent similarity: 30.11

Results for AtAGO3 vs AtAGO5 :
Alignment length: 1255
Identical residues: 274
Similar residues: 158
Percent identity: 21.83
Percent similarity: 34.42

Results for AtAGO3 vs AtAGO6 :
Alignment length: 1263
Identical residues: 251
Similar residues: 127
Percent identity: 19.87
Percent similarity: 29.93

Results for AtAGO3 vs AtAGO7 :
Alignment length: 1234
Identical residues: 302
Similar residues: 170
Percent identity: 24.47
Percent similarity: 38.25

Results for AtAGO3 vs AtAGO8 :
Alignment length: 1249
Identical residues: 227
Similar residues: 134
Percent identity: 18.17
Percent similarity: 28.90

Results for AtAGO3 vs AtAGO9 :
Alignment length: 1263
Identical residues: 239
Similar residues: 150
Percent identity: 18.92
Percent similarity: 30.80

Results for AtAGO3 vs AtAGO10 :
Alignment length: 1262
Identical residues: 274
Similar residues: 155
Percent identity: 21.71
Percent similarity: 33.99

Results for AtAGO3 vs HvAGO4A :
Alignment length: 1273
Identical residues: 235
Similar residues: 148
Percent identity: 18.46
Percent similarity: 30.09

Results for AtAGO3 vs HvAGO4B :
Alignment length: 1273
Identical residues: 200
Similar residues: 133
Percent identity: 15.71
Percent similarity: 26.16

Results for AtAGO3 vs HvAGO7 :

Alignment length: 1234
Identical residues: 313
Similar residues: 157
Percent identity: 25.36
Percent similarity: 38.09

Results for AtAGO3 vs HvAGO1D :
Alignment length: 1290
Identical residues: 277
Similar residues: 176
Percent identity: 21.47
Percent similarity: 35.12

Results for AtAGO3 vs HvAGO5B :
Alignment length: 1251
Identical residues: 259
Similar residues: 135
Percent identity: 20.70
Percent similarity: 31.49

Results for AtAGO3 vs HvAGO6 :
Alignment length: 1265
Identical residues: 237
Similar residues: 136
Percent identity: 18.74
Percent similarity: 29.49

Results for AtAGO3 vs HvAGO2 :
Alignment length: 1215
Identical residues: 400
Similar residues: 167
Percent identity: 32.92
Percent similarity: 46.67

Results for AtAGO3 vs HvAGO18 :
Alignment length: 1256
Identical residues: 301
Similar residues: 162
Percent identity: 23.96
Percent similarity: 36.86

Results for AtAGO3 vs HvAGO5A :
Alignment length: 1261
Identical residues: 293
Similar residues: 154
Percent identity: 23.24
Percent similarity: 35.45

Results for AtAGO3 vs HvAGO10 :
Alignment length: 1254
Identical residues: 273
Similar residues: 156
Percent identity: 21.77
Percent similarity: 34.21

Results for AtAGO3 vs HvAGO1A :
Alignment length: 1318
Identical residues: 295
Similar residues: 173
Percent identity: 22.38
Percent similarity: 35.51

Results for AtAGO4 vs AtAGO5 :
Alignment length: 1074
Identical residues: 306
Similar residues: 141
Percent identity: 28.49
Percent similarity: 41.62

Results for AtAGO4 vs AtAGO6 :
Alignment length: 930
Identical residues: 473
Similar residues: 105
Percent identity: 50.86
Percent similarity: 62.15

Results for AtAGO4 vs AtAGO7 :
Alignment length: 1077
Identical residues: 256
Similar residues: 147
Percent identity: 23.77
Percent similarity: 37.42

Results for AtAGO4 vs AtAGO8 :
Alignment length: 930
Identical residues: 538
Similar residues: 90
Percent identity: 57.85
Percent similarity: 67.53

Results for AtAGO4 vs AtAGO9 :
Alignment length: 931
Identical residues: 610
Similar residues: 103
Percent identity: 65.52
Percent similarity: 76.58

Results for AtAGO4 vs AtAGO10 :
Alignment length: 1046
Identical residues: 294
Similar residues: 157
Percent identity: 28.11
Percent similarity: 43.12

Results for AtAGO4 vs HvAGO4A :
Alignment length: 943
Identical residues: 596
Similar residues: 113
Percent identity: 63.20
Percent similarity: 75.19

Results for AtAGO4 vs HvAGO4B :
Alignment length: 946
Identical residues: 450
Similar residues: 111
Percent identity: 47.57
Percent similarity: 59.30

Results for AtAGO4 vs HvAGO7 :
Alignment length: 1085
Identical residues: 245
Similar residues: 154
Percent identity: 22.58
Percent similarity: 36.77

Results for AtAGO4 vs HvAGO1D :
Alignment length: 1122
Identical residues: 294
Similar residues: 159
Percent identity: 26.20
Percent similarity: 40.37

Results for AtAGO4 vs HvAGO5B :
Alignment length: 984
Identical residues: 285
Similar residues: 144
Percent identity: 28.96
Percent similarity: 43.60

Results for AtAGO4 vs HvAGO6 :
Alignment length: 926
Identical residues: 505
Similar residues: 121
Percent identity: 54.54
Percent similarity: 67.60

Results for AtAGO4 vs HvAGO2 :
Alignment length: 1146
Identical residues: 250
Similar residues: 136
Percent identity: 21.82
Percent similarity: 33.68

Results for AtAGO4 vs HvAGO18 :
Alignment length: 1135
Identical residues: 294
Similar residues: 158
Percent identity: 25.90
Percent similarity: 39.82

Results for AtAGO4 vs HvAGO5A :
Alignment length: 1109
Identical residues: 291
Similar residues: 161
Percent identity: 26.24
Percent similarity: 40.76

Results for AtAGO4 vs HvAGO10 :
Alignment length: 1020
Identical residues: 291
Similar residues: 159
Percent identity: 28.53
Percent similarity: 44.12

Results for AtAGO4 vs HvAGO1A :
Alignment length: 1271
Identical residues: 286
Similar residues: 162
Percent identity: 22.50
Percent similarity: 35.25

Results for AtAGO5 vs AtAGO6 :
Alignment length: 1056
Identical residues: 303
Similar residues: 130
Percent identity: 28.69
Percent similarity: 41.00

Results for AtAGO5 vs AtAGO7 :
Alignment length: 1104
Identical residues: 312
Similar residues: 140
Percent identity: 28.26
Percent similarity: 40.94

Results for AtAGO5 vs AtAGO8 :
Alignment length: 1052
Identical residues: 287

Similar residues: 117
Percent identity: 27.28
Percent similarity: 38.40

Results for AtAGO5 vs AtAGO9 :
Alignment length: 1064
Identical residues: 301
Similar residues: 139
Percent identity: 28.29
Percent similarity: 41.35

Results for AtAGO5 vs AtAGO10 :
Alignment length: 1051
Identical residues: 490
Similar residues: 136
Percent identity: 46.62
Percent similarity: 59.56

Results for AtAGO5 vs HvAGO4A :
Alignment length: 1083
Identical residues: 290
Similar residues: 135
Percent identity: 26.78
Percent similarity: 39.24

Results for AtAGO5 vs HvAGO4B :
Alignment length: 1066
Identical residues: 260
Similar residues: 130
Percent identity: 24.39
Percent similarity: 36.59

Results for AtAGO5 vs HvAGO7 :
Alignment length: 1099
Identical residues: 309
Similar residues: 152
Percent identity: 28.12
Percent similarity: 41.95

Results for AtAGO5 vs HvAGO1D :
Alignment length: 1110
Identical residues: 502
Similar residues: 133
Percent identity: 45.23
Percent similarity: 57.21

Results for AtAGO5 vs HvAGO5B :
Alignment length: 1015
Identical residues: 489
Similar residues: 121
Percent identity: 48.18
Percent similarity: 60.10

Results for AtAGO5 vs HvAGO6 :
Alignment length: 1060
Identical residues: 299
Similar residues: 120
Percent identity: 28.21
Percent similarity: 39.53

Results for AtAGO5 vs HvAGO2 :
Alignment length: 1134
Identical residues: 286
Similar residues: 143
Percent identity: 25.22
Percent similarity: 37.83

Results for AtAGO5 vs HvAGO18 :
Alignment length: 1105
Identical residues: 420
Similar residues: 136
Percent identity: 38.01
Percent similarity: 50.32

Results for AtAGO5 vs HvAGO5A :
Alignment length: 1059
Identical residues: 495
Similar residues: 167
Percent identity: 46.74
Percent similarity: 62.51

Results for AtAGO5 vs HvAGO10 :
Alignment length: 1039
Identical residues: 481
Similar residues: 127
Percent identity: 46.29
Percent similarity: 58.52

Results for AtAGO5 vs HvAGO1A :
Alignment length: 1245
Identical residues: 499
Similar residues: 132
Percent identity: 40.08
Percent similarity: 50.68

Results for AtAGO6 vs AtAGO7 :
Alignment length: 1065
Identical residues: 243
Similar residues: 134
Percent identity: 22.82
Percent similarity: 35.40

Results for AtAGO6 vs AtAGO8 :
Alignment length: 902
Identical residues: 428
Similar residues: 111
Percent identity: 47.45
Percent similarity: 59.76

Results for AtAGO6 vs AtAGO9 :
Alignment length: 908
Identical residues: 470
Similar residues: 116
Percent identity: 51.76
Percent similarity: 64.54

Results for AtAGO6 vs AtAGO10 :
Alignment length: 1038
Identical residues: 301
Similar residues: 118
Percent identity: 29.00
Percent similarity: 40.37

Results for AtAGO6 vs HvAGO4A :
Alignment length: 931
Identical residues: 478
Similar residues: 113
Percent identity: 51.34
Percent similarity: 63.48

Results for AtAGO6 vs HvAGO4B :
Alignment length: 915
Identical residues: 407
Similar residues: 89
Percent identity: 44.48
Percent similarity: 54.21

Results for AtAGO6 vs HvAGO7 :
Alignment length: 1072
Identical residues: 244
Similar residues: 146
Percent identity: 22.76
Percent similarity: 36.38

Results for AtAGO6 vs HvAGO1D :
Alignment length: 1115
Identical residues: 297
Similar residues: 134
Percent identity: 26.64
Percent similarity: 38.65

Results for AtAGO6 vs HvAGO5B :
Alignment length: 943
Identical residues: 279
Similar residues: 134
Percent identity: 29.59
Percent similarity: 43.80

Results for AtAGO6 vs HvAGO6 :
Alignment length: 893
Identical residues: 523
Similar residues: 99
Percent identity: 58.57
Percent similarity: 69.65

Results for AtAGO6 vs HvAGO2 :
Alignment length: 1133
Identical residues: 249
Similar residues: 130
Percent identity: 21.98
Percent similarity: 33.45

Results for AtAGO6 vs HvAGO18 :
Alignment length: 1125
Identical residues: 277
Similar residues: 135
Percent identity: 24.62
Percent similarity: 36.62

Results for AtAGO6 vs HvAGO5A :
Alignment length: 1091
Identical residues: 296
Similar residues: 122
Percent identity: 27.13
Percent similarity: 38.31

Results for AtAGO6 vs HvAGO10 :
Alignment length: 1008
Identical residues: 290
Similar residues: 126
Percent identity: 28.77
Percent similarity: 41.27

Results for AtAGO6 vs HvAGO1A :
Alignment length: 1263
Identical residues: 284
Similar residues: 138
Percent identity: 22.49

Percent similarity: 33.41

Results for AtAGO7 vs AtAGO8 :

Alignment length: 1060
Identical residues: 228
Similar residues: 126
Percent identity: 21.51
Percent similarity: 33.40

Results for AtAGO7 vs AtAGO9 :

Alignment length: 1067
Identical residues: 251
Similar residues: 139
Percent identity: 23.52
Percent similarity: 36.55

Results for AtAGO7 vs AtAGO10 :

Alignment length: 1112
Identical residues: 313
Similar residues: 130
Percent identity: 28.15
Percent similarity: 39.84

Results for AtAGO7 vs HvAGO4A :

Alignment length: 1076
Identical residues: 250
Similar residues: 144
Percent identity: 23.23
Percent similarity: 36.62

Results for AtAGO7 vs HvAGO4B :

Alignment length: 1074
Identical residues: 225
Similar residues: 127
Percent identity: 20.95
Percent similarity: 32.77

Results for AtAGO7 vs HvAGO7 :

Alignment length: 1025
Identical residues: 539
Similar residues: 134
Percent identity: 52.59
Percent similarity: 65.66

Results for AtAGO7 vs HvAGO1D :

Alignment length: 1167
Identical residues: 305
Similar residues: 153
Percent identity: 26.14
Percent similarity: 39.25

Results for AtAGO7 vs HvAGO5B :

Alignment length: 1063
Identical residues: 308
Similar residues: 116
Percent identity: 28.97
Percent similarity: 39.89

Results for AtAGO7 vs HvAGO6 :

Alignment length: 1070
Identical residues: 252
Similar residues: 142
Percent identity: 23.55
Percent similarity: 36.82

Results for AtAGO7 vs HvAGO2 :

Alignment length: 1103
Identical residues: 301
Similar residues: 147
Percent identity: 27.29
Percent similarity: 40.62

Results for AtAGO7 vs HvAGO18 :

Alignment length: 1126
Identical residues: 285
Similar residues: 163
Percent identity: 25.31
Percent similarity: 39.79

Results for AtAGO7 vs HvAGO5A :

Alignment length: 1118
Identical residues: 320
Similar residues: 138
Percent identity: 28.62
Percent similarity: 40.97

Results for AtAGO7 vs HvAGO10 :

Alignment length: 1092
Identical residues: 298
Similar residues: 147
Percent identity: 27.29
Percent similarity: 40.75

Results for AtAGO7 vs HvAGO1A :

Alignment length: 1288
Identical residues: 300
Similar residues: 136
Percent identity: 23.29
Percent similarity: 33.85

Results for AtAGO8 vs AtAGO9 :

Alignment length: 903
Identical residues: 593
Similar residues: 80
Percent identity: 65.67
Percent similarity: 74.53

Results for AtAGO8 vs AtAGO10 :

Alignment length: 1026
Identical residues: 284
Similar residues: 134
Percent identity: 27.68
Percent similarity: 40.74

Results for AtAGO8 vs HvAGO4A :

Alignment length: 928
Identical residues: 506
Similar residues: 102
Percent identity: 54.53
Percent similarity: 65.52

Results for AtAGO8 vs HvAGO4B :

Alignment length: 916
Identical residues: 396
Similar residues: 97
Percent identity: 43.23
Percent similarity: 53.82

Results for AtAGO8 vs HvAGO7 :

Alignment length: 1067
Identical residues: 229
Similar residues: 139
Percent identity: 21.46
Percent similarity: 34.49

Results for AtAGO8 vs HvAGO1D :

Alignment length: 1102
Identical residues: 276
Similar residues: 127
Percent identity: 25.05
Percent similarity: 36.57

Results for AtAGO8 vs HvAGO5B :

Alignment length: 934
Identical residues: 261
Similar residues: 128
Percent identity: 27.94
Percent similarity: 41.65

Results for AtAGO8 vs HvAGO6 :

Alignment length: 896
Identical residues: 451
Similar residues: 118
Percent identity: 50.33
Percent similarity: 63.50

Results for AtAGO8 vs HvAGO2 :

Alignment length: 1122
Identical residues: 239
Similar residues: 119
Percent identity: 21.30
Percent similarity: 31.91

Results for AtAGO8 vs HvAGO18 :

Alignment length: 1114
Identical residues: 262
Similar residues: 135
Percent identity: 23.52
Percent similarity: 35.64

Results for AtAGO8 vs HvAGO5A :

Alignment length: 1088
Identical residues: 277
Similar residues: 130
Percent identity: 25.46
Percent similarity: 37.41

Results for AtAGO8 vs HvAGO10 :

Alignment length: 998
Identical residues: 273
Similar residues: 129
Percent identity: 27.35
Percent similarity: 40.28

Results for AtAGO8 vs HvAGO1A :

Alignment length: 1253
Identical residues: 265
Similar residues: 136
Percent identity: 21.15
Percent similarity: 32.00

Results for AtAGO9 vs AtAGO10 :

Alignment length: 1037
Identical residues: 298
Similar residues: 154
Percent identity: 28.74
Percent similarity: 43.59

Results for AtAGO9 vs HvAGO4A :
Alignment length: 932
Identical residues: 577
Similar residues: 105
Percent identity: 61.91
Percent similarity: 73.18

Results for AtAGO9 vs HvAGO4B :
Alignment length: 927
Identical residues: 433
Similar residues: 112
Percent identity: 46.71
Percent similarity: 58.79

Results for AtAGO9 vs HvAGO7 :
Alignment length: 1075
Identical residues: 244
Similar residues: 167
Percent identity: 22.70
Percent similarity: 38.23

Results for AtAGO9 vs HvAGO1D :
Alignment length: 1114
Identical residues: 297
Similar residues: 149
Percent identity: 26.66
Percent similarity: 40.04

Results for AtAGO9 vs HvAGO5B :
Alignment length: 957
Identical residues: 285
Similar residues: 147
Percent identity: 29.78
Percent similarity: 45.14

Results for AtAGO9 vs HvAGO6 :
Alignment length: 904
Identical residues: 505
Similar residues: 115
Percent identity: 55.86
Percent similarity: 68.58

Results for AtAGO9 vs HvAGO2 :
Alignment length: 1134
Identical residues: 258
Similar residues: 142
Percent identity: 22.75
Percent similarity: 35.27

Results for AtAGO9 vs HvAGO18 :
Alignment length: 1125
Identical residues: 283
Similar residues: 149
Percent identity: 25.16
Percent similarity: 38.40

Results for AtAGO9 vs HvAGO5A :
Alignment length: 1100
Identical residues: 289
Similar residues: 153
Percent identity: 26.27
Percent similarity: 40.18

Results for AtAGO9 vs HvAGO10 :
Alignment length: 1009
Identical residues: 286
Similar residues: 155
Percent identity: 28.34
Percent similarity: 43.71

Results for AtAGO9 vs HvAGO1A :
Alignment length: 1263
Identical residues: 279
Similar residues: 158
Percent identity: 22.09
Percent similarity: 34.60

Results for AtAGO10 vs HvAGO4A :
Alignment length: 1056
Identical residues: 293
Similar residues: 153
Percent identity: 27.75
Percent similarity: 42.23

Results for AtAGO10 vs HvAGO4B :
Alignment length: 1045
Identical residues: 269
Similar residues: 127
Percent identity: 25.74
Percent similarity: 37.89

Results for AtAGO10 vs HvAGO7 :
Alignment length: 1104
Identical residues: 300
Similar residues: 139
Percent identity: 27.17
Percent similarity: 39.76

Results for AtAGO10 vs HvAGO1D :
Alignment length: 1078
Identical residues: 611
Similar residues: 114
Percent identity: 56.68
Percent similarity: 67.25

Results for AtAGO10 vs HvAGO5B :
Alignment length: 1011
Identical residues: 466
Similar residues: 131
Percent identity: 46.09
Percent similarity: 59.05

Results for AtAGO10 vs HvAGO6 :
Alignment length: 1038
Identical residues: 293
Similar residues: 136
Percent identity: 28.23
Percent similarity: 41.33

Results for AtAGO10 vs HvAGO2 :
Alignment length: 1144
Identical residues: 270
Similar residues: 140
Percent identity: 23.60
Percent similarity: 35.84

Results for AtAGO10 vs HvAGO18 :
Alignment length: 1106
Identical residues: 450
Similar residues: 127
Percent identity: 40.69
Percent similarity: 52.17

Results for AtAGO10 vs HvAGO5A :
Alignment length: 1080
Identical residues: 466
Similar residues: 154
Percent identity: 43.15
Percent similarity: 57.41

Results for AtAGO10 vs HvAGO10 :
Alignment length: 989
Identical residues: 646
Similar residues: 112
Percent identity: 65.32
Percent similarity: 76.64

Results for AtAGO10 vs HvAGO1A :
Alignment length: 1236
Identical residues: 628
Similar residues: 106
Percent identity: 50.81
Percent similarity: 59.39

Results for HvAGO4A vs HvAGO4B :
Alignment length: 945
Identical residues: 484
Similar residues: 102
Percent identity: 51.22
Percent similarity: 62.01

Results for HvAGO4A vs HvAGO7 :
Alignment length: 1082
Identical residues: 243
Similar residues: 161
Percent identity: 22.46
Percent similarity: 37.34

Results for HvAGO4A vs HvAGO1D :
Alignment length: 1121
Identical residues: 297
Similar residues: 147
Percent identity: 26.49
Percent similarity: 39.61

Results for HvAGO4A vs HvAGO5B :
Alignment length: 983
Identical residues: 271
Similar residues: 145
Percent identity: 27.57
Percent similarity: 42.32

Results for HvAGO4A vs HvAGO6 :
Alignment length: 927
Identical residues: 521
Similar residues: 108
Percent identity: 56.20
Percent similarity: 67.85

Results for HvAGO4A vs HvAGO2 :
Alignment length: 1147
Identical residues: 256
Similar residues: 136
Percent identity: 22.32
Percent similarity: 34.18

Results for HvAGO4A vs HvAGO18 :
Alignment length: 1134

Identical residues: 284
Similar residues: 150
Percent identity: 25.04
Percent similarity: 38.27

Results for HvAGO4A vs HvAGO5A :
Alignment length: 1109
Identical residues: 292
Similar residues: 144
Percent identity: 26.33
Percent similarity: 39.31

Results for HvAGO4A vs HvAGO10 :
Alignment length: 1031
Identical residues: 288
Similar residues: 145
Percent identity: 27.93
Percent similarity: 42.00

Results for HvAGO4A vs HvAGO1A :
Alignment length: 1271
Identical residues: 285
Similar residues: 150
Percent identity: 22.42
Percent similarity: 34.23

Results for HvAGO4B vs HvAGO7 :
Alignment length: 1082
Identical residues: 234
Similar residues: 138
Percent identity: 21.63
Percent similarity: 34.38

Results for HvAGO4B vs HvAGO1D :
Alignment length: 1122
Identical residues: 275
Similar residues: 135
Percent identity: 24.51
Percent similarity: 36.54

Results for HvAGO4B vs HvAGO5B :
Alignment length: 921
Identical residues: 258
Similar residues: 134
Percent identity: 28.01
Percent similarity: 42.56

Results for HvAGO4B vs HvAGO6 :
Alignment length: 910
Identical residues: 420
Similar residues: 101
Percent identity: 46.15
Percent similarity: 57.25

Results for HvAGO4B vs HvAGO2 :
Alignment length: 1138
Identical residues: 220
Similar residues: 127
Percent identity: 19.33
Percent similarity: 30.49

Results for HvAGO4B vs HvAGO18 :
Alignment length: 1136
Identical residues: 268
Similar residues: 129
Percent identity: 23.59
Percent similarity: 34.95

Results for HvAGO4B vs HvAGO5A :
Alignment length: 1095
Identical residues: 269
Similar residues: 122
Percent identity: 24.57
Percent similarity: 35.71

Results for HvAGO4B vs HvAGO10 :
Alignment length: 1014
Identical residues: 263
Similar residues: 129
Percent identity: 25.94
Percent similarity: 38.66

Results for HvAGO4B vs HvAGO1A :
Alignment length: 1269
Identical residues: 260
Similar residues: 133
Percent identity: 20.49
Percent similarity: 30.97

Results for HvAGO7 vs HvAGO1D :
Alignment length: 1160
Identical residues: 315
Similar residues: 159
Percent identity: 27.16
Percent similarity: 40.86

Results for HvAGO7 vs HvAGO5B :
Alignment length: 1067
Identical residues: 306
Similar residues: 131
Percent identity: 28.68
Percent similarity: 40.96

Results for HvAGO7 vs HvAGO6 :
Alignment length: 1077
Identical residues: 234
Similar residues: 160
Percent identity: 21.73
Percent similarity: 36.58

Results for HvAGO7 vs HvAGO2 :
Alignment length: 1105
Identical residues: 313
Similar residues: 157
Percent identity: 28.33
Percent similarity: 42.53

Results for HvAGO7 vs HvAGO18 :
Alignment length: 1128
Identical residues: 303
Similar residues: 153
Percent identity: 26.86
Percent similarity: 40.43

Results for HvAGO7 vs HvAGO5A :
Alignment length: 1119
Identical residues: 319
Similar residues: 141
Percent identity: 28.51
Percent similarity: 41.11

Results for HvAGO7 vs HvAGO10 :
Alignment length: 1087
Identical residues: 296
Similar residues: 149
Percent identity: 27.23
Percent similarity: 40.94

Results for HvAGO7 vs HvAGO1A :
Alignment length: 1287
Identical residues: 298
Similar residues: 144
Percent identity: 23.15
Percent similarity: 34.34

Results for HvAGO1D vs HvAGO5B :
Alignment length: 1083
Identical residues: 464
Similar residues: 128
Percent identity: 42.84
Percent similarity: 54.66

Results for HvAGO1D vs HvAGO6 :
Alignment length: 1115
Identical residues: 299
Similar residues: 135
Percent identity: 26.82
Percent similarity: 38.92

Results for HvAGO1D vs HvAGO2 :
Alignment length: 1186
Identical residues: 294
Similar residues: 158
Percent identity: 24.79
Percent similarity: 38.11

Results for HvAGO1D vs HvAGO18 :
Alignment length: 1147
Identical residues: 472
Similar residues: 138
Percent identity: 41.15
Percent similarity: 53.18

Results for HvAGO1D vs HvAGO5A :
Alignment length: 1126
Identical residues: 497
Similar residues: 157
Percent identity: 44.14
Percent similarity: 58.08

Results for HvAGO1D vs HvAGO10 :
Alignment length: 1071
Identical residues: 581
Similar residues: 118
Percent identity: 54.25
Percent similarity: 65.27

Results for HvAGO1D vs HvAGO1A :
Alignment length: 1269
Identical residues: 663
Similar residues: 107
Percent identity: 52.25
Percent similarity: 60.68

Results for HvAGO5B vs HvAGO6 :
Alignment length: 943
Identical residues: 281
Similar residues: 128

Percent identity: 29.80
Percent similarity: 43.37

Results for HvAGO5B vs HvAGO2 :
Alignment length: 1115
Identical residues: 274
Similar residues: 134
Percent identity: 24.57
Percent similarity: 36.59

Results for HvAGO5B vs HvAGO18 :
Alignment length: 1099
Identical residues: 395
Similar residues: 126
Percent identity: 35.94
Percent similarity: 47.41

Results for HvAGO5B vs HvAGO5A :
Alignment length: 1048
Identical residues: 484
Similar residues: 123
Percent identity: 46.18
Percent similarity: 57.92

Results for HvAGO5B vs HvAGO10 :
Alignment length: 978
Identical residues: 449
Similar residues: 133
Percent identity: 45.91
Percent similarity: 59.51

Results for HvAGO5B vs HvAGO1A :
Alignment length: 1233
Identical residues: 453
Similar residues: 120
Percent identity: 36.74
Percent similarity: 46.47

Results for HvAGO6 vs HvAGO2 :
Alignment length: 1137
Identical residues: 243
Similar residues: 125
Percent identity: 21.37
Percent similarity: 32.37

Results for HvAGO6 vs HvAGO18 :
Alignment length: 1128
Identical residues: 285
Similar residues: 132
Percent identity: 25.27
Percent similarity: 36.97

Results for HvAGO6 vs HvAGO5A :
Alignment length: 1098
Identical residues: 302
Similar residues: 121
Percent identity: 27.50
Percent similarity: 38.52

Results for HvAGO6 vs HvAGO10 :
Alignment length: 1010
Identical residues: 300
Similar residues: 132
Percent identity: 29.70
Percent similarity: 42.77

Results for HvAGO6 vs HvAGO1A :
Alignment length: 1264
Identical residues: 283
Similar residues: 138
Percent identity: 22.39
Percent similarity: 33.31

Results for HvAGO2 vs HvAGO18 :
Alignment length: 1142
Identical residues: 294
Similar residues: 164
Percent identity: 25.74
Percent similarity: 40.11

Results for HvAGO2 vs HvAGO5A :
Alignment length: 1138
Identical residues: 305
Similar residues: 141
Percent identity: 26.80
Percent similarity: 39.19

Results for HvAGO2 vs HvAGO10 :
Alignment length: 1131
Identical residues: 266
Similar residues: 148
Percent identity: 23.52
Percent similarity: 36.60

Results for HvAGO2 vs HvAGO1A :
Alignment length: 1310
Identical residues: 295
Similar residues: 151
Percent identity: 22.52
Percent similarity: 34.05

Results for HvAGO18 vs HvAGO5A :
Alignment length: 1112
Identical residues: 436
Similar residues: 146
Percent identity: 39.21
Percent similarity: 52.34

Results for HvAGO18 vs HvAGO10 :
Alignment length: 1094
Identical residues: 427
Similar residues: 145
Percent identity: 39.03
Percent similarity: 52.29

Results for HvAGO18 vs HvAGO1A :
Alignment length: 1279
Identical residues: 446
Similar residues: 140
Percent identity: 34.87
Percent similarity: 45.82

Results for HvAGO5A vs HvAGO10 :
Alignment length: 1070
Identical residues: 454
Similar residues: 153
Percent identity: 42.43
Percent similarity: 56.73

Results for HvAGO5A vs HvAGO1A :
Alignment length: 1246
Identical residues: 484
Similar residues: 161
Percent identity: 38.84
Percent similarity: 51.77

Results for HvAGO10 vs HvAGO1A :
Alignment length: 1228
Identical residues: 580
Similar residues: 108
Percent identity: 47.23
Percent similarity: 56.03
