# Supplementary material for: Genome-Wide Identification of RNA Silencing-Related Genes and Their Expressional Analysis in Response to Heat Stress in Barley (Hordeum vulgare L.)
Source: Biomolecules. 2020 Jun 18;10(6):929. doi: 10.3390/biom10060929 (PMC7356095; doi:10.3390/biom10060929)
Supplement: Supplementary file 1 [file biomolecules-10-00929-s001.zip › Supplementary_tables.pdf]

Table S1. List of primers used for semiquantitative PCR

primer name	sequence	Tm (°C)	product length (bp, with cDNA template)	product length (bp, with gDNA template)
Hv_actin_Fw	AATGGAACCGGAATGGTCAAG			
Hv_actin_Rv	CTCGTAGCTCTTCTCAACTGAGGAG	66	690	690
HvAGO1a_Fw	CACCTGAATATCAACAGCGTGACTAC			
HvAGO1a_Rv	CTGGCAGTAGGCAATTCCCGT	66,7	1290	1418
HvAGO1d_Fw	GTTCCACATCACCGTGCTCGA			
HvAGO1d_Rv	CAGGCGCTCGCTGATCTTAATAC	68	871	2457
HvAGO2_Fw	ATGTCAAGCCAGGGTCCAGCT			
HvAGO2_Rv	GATCACCTTGCCATACTGCTGAG	67,2	826	826
HvAGO4_Fw	CATGAGTGCTATTGCAATGGC			
HvAGO4_Rv	ACTTTGTGGACTTGGGTGGAG	68,5	170	286
HvAGO5a_Fw	GTCGAGCAAGTCGACGGAGTTC			
HvAGO5a_Rv	AGCGATATCAAGAGCCTGGAGAGTG	72	447	2485
HvAGO5b_Fw	CATATGATGGCAGCAAGAGCC			
HvAGO5b_Rv	TCCTCAGATTCAGACACTCCTGAA	67	437	769
HvAGO6_Fw	GTCCATCAAATCTGATGACGATCAGG			
HvAGO6_Rv	TGGAGTCCACGACAACCACTGACT	70,5	494	1420
HvAGO7_Fw	CAGTCATGTTACTGATGGCTCCAAG			
HvAGO7_Rv	CTGACCTCTCAAGGTACAGCCTG	67	1184	1184
HvAGO10_Fw	GTGAAGACCAACCACTTCCTC			
HvAGO10_Rv	CTTTAAATCACGTAGGGCCTTC	63	659	1144
HvAGO18_Fw	CTACAGGTGCTAGACATTGTGTTGC			
HvAGO18_Rv	GCTGTCCTGGAACTATATTGCAGAC	69	584	1034
HvDCL1_Fw	GTAGACGACGGATTCGTCGCC			
HvDCL1_Rv	CAGCAGCTTCCATTTGTTTGATCTG	69,7	1554	2793
HvDCL2_Fw	GACTCAGCCGTGACAGAGGAAGC			
HvDCL2_Rv	GATCTGCGGCGTCATGACAAG	70,5	360	444
HvDCL3_Fw	CTCAAGAGGTCATCCGAGTCATG			
HvDCL3_Rv	GGTGAGGCCGTCATACCAAAC	67,8	569	1368
HvDCL5_Fw	cGAGGTGATTCGGGAGTACAC			
HvDCL5_Rv	GTAGCAACAGGAGATGCTGTCATTC	66	284	1057
HvDCL4_Fw	GAAGACCCATATAGCCGTGCTG			
HvDCL4_Rv	GAAACAATGCCGCAAACTATGC	66,9	271	751
HvRDR1a_Fw	CTTCAGGTGTCAGGATTTCCTG			
HvRDR1a_Rv	GTGATGTTAACATGTGGCGTGA	68	1030	1464
HvRDR1b_Fw	CTGTCAATGCTGACCATGTCAAG			
HvRDR1b_Rv	CCACTGTGATGTTGGATCAATAGC	68,2	1780	2269
HvRDR2_Fw	TCACGATTCTCAGATGTTCAAG			
HvRDR2_Rv	CTCTCAGAGATCTTCCCGATAC	64	1200	4500
HvRDR3_Fw	CAGTTGCAATGGAGGTTGACA			
HvRDR3_Rv	CAAACCCAAGATGATGCCATC	69	881	2126
HvRDR4_Fw	GCAAGATAACTTGGGTCCTGTCAGAG			
HvRDR4_Rv	AATGAAGCATGGAAAGGTACTTCAAGG	71,7	767	943
HvRDR6a_Fw	GTGTTCAAGCTCGACTGCTCG			
HvRDR6a_Rv	GTGGACCTTATAGCCAACAGCAATG	70,8	1637	1637
HvRDR6b_Fw	GTGACGACGCAAGTGATCGTC			
HvRDR6b_Rv	GATTGTGACACGCATCCATGC	71,5	1220	1220

Table S2: List of primers used for RT-qPCR

primer name	sequence	Tm (°C)	product length (bp, with cDNA template)	product length (bp, with gDNA template)
Hv_actin_qFw	CGACAATGGAACCGGAATG			
Hv_actin_qRv	CCCTTGGCGCATCATCTC	58	56	157
HvAGO1a_qFw	AACAAGAAAATGGTCAATGGTGG			
HvAGO1a_qRv	GACAGAAAACCTTGGCAGCA	60	94	-
HvAGO2_qFw	CCTTGCAGGGGAGAAATAGCT			
HvAGO2_qRv	TGTCAGCGAGGGAGGAGTAA	60	96	-
HvAGO6_qFw	AGCTACACACAACAACATGGAA			
HvAGO6_qRv	TCACTGCTTCCACTCCGAAC	59.9	99	513
HvDCL1_qFw	CCGCAGTTAAGATATTAGAAGCC			
HvDCL1_qRv	TCTACTCACGACCCATTTCAGG	59.5	110	-
HvDCL3_qFw	AGAGGGCCTAAGGTTCTTGG			
HvDCL3_qRv	ACCCCAAACTACCTCCAAATCA	59.3	90	-
HvRDR2_qFw	TGAAGCTGTCTATGACCATGG			
HvRDR2_qRv	AGAGGTCACCGTCCAAATCG	59.7	122	358
HvRDR6a_qFw	TTGCAGAGGACAGAACGGAC			
HvRDR6a_qRv	GCATAGGTCGACGAGAAGCA	59.9	127	127

Table S3. number of exons in barley RNAi genes and their *Arabidopsis* homologues

Barley RNAi factor	nr. of exons	*Arabidopsis* RNAi factor	nr. of exons
HvAGO1a	23	*AtAGO1*	22
HvAGO1d	22	*AtAGO1*	22
HvAGO2	3	*AtAGO2*	3
HvAGO4a	23	*AtAGO4*	20
HvAGO4b	22	-	
HvAGO5a	22	*AtAGO5*	20
HvAGO5b	22	*AtAGO5*	20
HvAGO6	23	*AtAGO6*	23
HvAGO7	3	*AtAGO7*	3
HvAGO10	21	*AtAGO10*	20
HvAGO18	21	-	-
HvDCL1	19	*AtDCL1*	20
HvDCL2	20	*AtDCL2*	23
HvDCL3	26	*AtDCL3*	24
HvDCL5	26	*AtDCL3*	24
HvDCL4	25	*AtDCL4*	25
HvRDR1a	5	*AtRDR1*	4
HvRDR1b	5	*AtRDR1*	4
HvRDR2	4	*AtRDR2*	4
HvRDR3	19	*AtRDR3a*	18
HvRDR4	19	*AtRDR3b*	18
HvRDR6a	2	*AtRDR6*	2
HvRDR6b	6	*AtRDR6*	2

Table S4. Domain organisation of barley RNAi-related proteins according to Pfam

HvDCL1	Family	Description	Entry	Clan	Envelope		Alignment		HMM		HMM	E-value
			type		Start	End	Start	End	From	To	length	
	ResIII	Type III restriction enzyme, res subunit	Family	CL0023	307	485	323	483	17	169	171	2.2e-13
	Helicase_C	Helicase conserved C-terminal domain	Family	CL0023	716	837	719	834	4	108	111	7.2e-17
	Dicer_dimer	Dicer dimerisation domain	Domain	CL0196	908	999	908	996	1	88	92	2.2e-20
	PAZ	PAZ domain	Domain	CL0638	1279	1434	1288	1432	14	135	137	1.8e-24
	Ribonuclease_3	Ribonuclease III domain	Family	CL0539	1469	1615	1469	1615	1	105	105	4.1e-29
	Ribonuclease_3	Ribonuclease III domain	Family	CL0539	1690	1803	1690	1803	1	105	105	4.1e-23

	DND1_DSRM	double strand RNA binding domain from DE…	Domain	CL0196	1911	1986	1913	1985	3	79	80	2.3e-14

HvDCL2	Family	Description	Entry	Clan	Envelope		Alignment		HMM		HMM	E-value
			type		Start	End	Start	End	From	To	length	
	DEAD	DEAD/DEAH box helicase	Domain	CL0023	104	279	106	273	3	170	176	2.3e-15
	Helicase_C	Helicase conserved C-terminal domain	Family	CL0023	449	570	451	568	3	109	111	3.6e-15
	Dicer_dimer	Dicer dimerisation domain	Domain	CL0196	638	719	638	717	1	88	92	5.3e-16
	PAZ	PAZ domain	Domain	CL0638	891	1037	903	1037	15	137	137	2,00E-16
	Ribonuclease_3	Ribonuclease III domain	Family	CL0539	1077	1195	1077	1191	1	101	105	1.6e-22
	Ribonuclease_3	Ribonuclease III domain	Family	CL0539	1268	1380	1268	1379	1	104	105	1.7e-19
	dsrm	Double-stranded RNA binding motif	Domain	CL0196	1407	1470	1407	1470	1	67	67	8.4e-06

HvDCL3	Family	Description	Entry	Clan	Envelope		Alignment		HMM		HMM	E-value
			type		Start	End	Start	End	From	To	length	
	DEAD	DEAD/DEAH box helicase	Domain	CL0023	90	257	93	251	4	167	176	3.5e-14
	Helicase_C	Helicase conserved C-terminal domain	Family	CL0023	456	576	469	573	13	107	111	6.2e-18
	Dicer_dimer	Dicer dimerisation domain	Domain	CL0196	644	728	644	724	1	85	92	2.8e-20
	PAZ	PAZ domain	Domain	CL0638	941	1088	952	1087	25	136	137	6.9e-14
	Ribonuclease_3	Ribonuclease III domain	Family	CL0539	1126	1254	1126	1254	1	105	105	1.6e-23
	Ribonuclease_3	Ribonuclease III domain	Family	CL0539	1330	1442	1330	1441	1	104	105	1.3e-23

HvDCL4	Family	Description	Entry	Clan	Envelope		Alignment		HMM		HMM	E-value
			type		Start	End	Start	End	From	To	length	
	DEAD	DEAD/DEAH box helicase	Domain	CL0023	39	204	40	201	3	167	176	4.7e-13
	Helicase_C	Helicase conserved C-terminal domain	Family	CL0023	381	502	382	500	2	109	111	7.3e-18
	Dicer_dimer	Dicer dimerisation domain	Domain	CL0196	566	653	566	649	1	88	92	1.6e-21
	PAZ	PAZ domain	Domain	CL0638	852	980	860	978	25	135	137	7.1e-10
	Ribonuclease_3	Ribonuclease III domain	Family	CL0539	1021	1146	1021	1146	1	105	105	2.7e-20
	Ribonuclease_3	Ribonuclease III domain	Family	CL0539	1222	1331	1222	1331	1	105	105	9.5e-23
	DND1_DSRM	double strand RNA binding domain from DE…	Domain	CL0196	1547	1623	1548	1623	2	80	80	1.3e-14

HvDCL5	Family	Description	Entry	Clan	Envelope		Alignment		HMM		HMM	E-value
			type		Start	End	Start	End	From	To	length	
	DEAD	DEAD/DEAH box helicase	Domain	CL0023	88	250	89	225	2	136	176	1.6e-17
	Helicase_C	Helicase conserved C-terminal domain	Family	CL0023	441	563	442	561	2	109	111	2.7e-18
	Dicer_dimer	Dicer dimerisation domain	Domain	CL0196	631	718	632	717	2	91	92	2.2e-24
	PAZ	PAZ domain	Domain	CL0638	929	1076	943	1074	26	135	137	6.3e-12
	Ribonuclease_3	Ribonuclease III domain	Family	CL0539	1113	1248	1113	1247	1	104	105	6.5e-22
	Ribonuclease_3	Ribonuclease III domain	Family	CL0539	1325	1437	1325	1436	1	104	105	3.6e-20
	dsrm	Double-stranded RNA binding motif	Domain	CL0196	1464	1527	1464	1527	1	67	67	0.00013

HvAGO1a	Family	Description	Entry	Clan	Envelope		Alignment		HMM		HMM	E-value

			type		Start	End	Start	End	From	To	length	
	Gly-rich_Ago1	Glycine-rich region of argonaut	Domain	n/a	226	336	229	336	4	106	106	5.7e-36
	ArgoN	N-terminal domain of argonaute	Domain	n/a	354	493	355	493	2	138	138	1.9e-32
	ArgoL1	Argonaute linker 1 domain	Domain	n/a	503	553	503	552	1	51	52	1.3e-21
	PAZ	PAZ domain	Domain	CL0638	558	686	571	684	12	135	137	5.2e-27
	ArgoL2	Argonaute linker 2 domain	Family	n/a	695	741	695	741	1	47	47	8.2e-17
	ArgoMid	Mid domain of argonaute	Domain	n/a	751	830	752	825	2	78	84	2.7e-08
	Piwi	Piwi domain	Family	CL0219	845	1167	846	1166	2	301	302	5.3e-87

**HvAGO1d**	**Family**	**Description**	**Entry**	**Clan**	**Envelope**		**Alignment**		**HMM**		**HMM**	**E-value**
			type		Start	End	Start	End	From	To	length	
	ArgoN	N-terminal domain of argonaute	Domain	n/a	209	343	210	343	2	138	138	5.8e-32
	ArgoL1	Argonaute linker 1 domain	Domain	n/a	353	403	355	402	3	51	52	1,00E-19
	PAZ	PAZ domain	Domain	CL0638	408	536	414	531	7	132	137	2.8e-25
	ArgoL2	Argonaute linker 2 domain	Family	n/a	545	591	545	591	1	47	47	5.1e-15
	ArgoMid	Mid domain of argonaute	Domain	n/a	601	678	602	673	2	76	84	4.7e-09
	Piwi	Piwi domain	Family	CL0219	695	1016	696	1015	2	301	302	2.8e-110

**HvAGO2**	**Family**	**Description**	**Entry**	**Clan**	**Envelope**		**Alignment**		**HMM**		**HMM**	**E-value**
			type		Start	End	Start	End	From	To	length	
	ArgoN	N-terminal domain of argonaute	Domain	n/a	215	340	216	340	2	138	138	1.4e-13
	ArgoL1	Argonaute linker 1 domain	Domain	n/a	351	398	352	398	3	52	52	4.7e-08

	PAZ	PAZ domain	Domain	CL0638	403	533	417	531	11	133	137	5.2e-21
	ArgoL2	Argonaute linker 2 domain	Family	n/a	543	590	543	589	1	46	47	1.1e-07
	ArgoMid	Mid domain of argonaute	Domain	n/a	603	679	603	670	1	66	84	1.7e-06
	Piwi	Piwi domain	Family	CL0219	703	1004	710	1003	9	301	302	3.7e-89

**HvAGO4a**	**Family**	**Description**	**Entry**	**Clan**	**Envelope**		**Alignment**		**HMM**		**HMM**	**E-value**
			type		Start	End	Start	End	From	To	length	
	ArgoN	N-terminal domain of argonaute	Domain	n/a	67	226	68	226	2	138	138	6.8e-29
	ArgoL1	Argonaute linker 1 domain	Domain	n/a	237	287	238	286	2	51	52	2.5e-17
	PAZ	PAZ domain	Domain	CL0638	292	422	293	421	2	136	137	2,00E-25
	ArgoL2	Argonaute linker 2 domain	Family	n/a	431	477	431	476	1	46	47	4.5e-11
	Piwi	Piwi domain	Family	CL0219	574	882	575	882	2	302	302	1.5e-101

**HvAGO4bb**	**Family**	**Description**	**Entry**	**Clan**	**Envelop**		**Alignmentnt**		**HMM**		**HMMM**	**E-value**
	Piwi	Piwi domain	Family	CL0219	469	783	470	782	2	301	302	4.3e-86
	PAZ	PAZ domain	Domain	CL0638n/a	192	317	193	316	2	136	137	2.5e-22
	ArgoL1	Argonaute-linker 2 domain	Domain	n/a	138	187	139	186	2	51	52	8.3e-15
			Family									
			Domain									
	ArgoL2	Argonaute linker 2 domain	Family	n/a	326	372	326	370	1	45	47	5.7e-13
	ArgoN	N-terminal domain	Domain	n/a	3	127	13	127	35	138	138	5.1e-12

**HvAGO5a**	**Family**	**Description**	**Entry**	**Clan**	**Envelope**		**Alignment**		**HMM**		**HMM**	**E-value**
			type		Start	End	Start	End	From	To	length	

	ArgoN	N-terminal domain of argonaute	Domain	n/a	185	317	185	317	1	138	138	2,00E-28
	ArgoL1	Argonaute linker 1 domain	Domain	n/a	327	377	327	377	1	52	52	4.8e-21
	PAZ	PAZ domain	Domain	CL0638	382	509	395	508	11	136	137	1.1e-22
	ArgoL2	Argonaute linker 2 domain	Family	n/a	518	564	519	564	2	47	47	1.5e-11
	ArgoMid	Mid domain of argonaute	Domain	n/a	574	653	575	646	2	76	84	2.8e-12
	Piwi	Piwi domain	Family	CL0219	670	993	671	991	2	300	302	1.2e-105

**HvAGO5b**	**Family**	**Description**	**Entry**	**Clan**	**Envelope**		**Alignment**		**HMM**		**HMM**	**E-value**
			type		Start	End	Start	End	From	To	length	
	ArgoN	N-terminal domain of argonaute	Domain	n/a	4	132	4	132	1	138	138	8.5e-30
	ArgoL1	Argonaute linker 1 domain	Domain	n/a	142	191	143	191	2	52	52	7.3e-19
	PAZ	PAZ domain	Domain	CL0638	193	325	199	322	4	134	137	4.6e-23
	ArgoL2	Argonaute linker 2 domain	Family	n/a	334	380	334	380	1	47	47	1,00E-15
	ArgoMid	Mid domain of argonaute	Domain	n/a	390	469	391	468	2	83	84	7.7e-12
	Piwi	Piwi domain	Family	CL0219	475	794	476	792	2	300	302	4.2e-108

**HvAGO6**	**Family**	**Description**	**Entry**	**Clan**	**Envelope**		**Alignment**		**HMM**		**HMM**	**E-value**
			type		Start	End	Start	End	From	To	length	
	ArgoN	N-terminal domain of argonaute	Domain	n/a	32	190	32	190	1	138	138	4.1e-27
	ArgoL1	Argonaute linker 1 domain	Domain	n/a	201	251	202	249	2	50	52	2.7e-13
	PAZ	PAZ domain	Domain	CL0638	256	383	257	382	2	136	137	3.3e-23
	ArgoL2	Argonaute linker 2 domain	Family	n/a	392	438	392	436	1	45	47	4.6e-17

	Piwi	Piwi domain	Family	CL0219	535	844	536	844	2	302	302	2.5e-100

HvAGO7	Family	Description	Entry	Clan	Envelope		Alignment		HMM		HMM	E-value
			type		Start	End	Start	End	From	To	length	
	ArgoN	N-terminal domain of argonaute	Domain	n/a	165	310	167	310	3	138	138	1.2e-22
	ArgoL1	Argonaute linker 1 domain	Domain	n/a	320	370	321	369	2	51	52	8.8e-14
	PAZ	PAZ domain	Domain	CL0638	382	505	383	504	2	136	137	1.1e-21
	ArgoL2	Argonaute linker 2 domain	Family	n/a	514	561	514	561	1	47	47	2,00E-07
	Piwi	Piwi domain	Family	CL0219	667	975	668	972	2	299	302	2.3e-92

HvAGO10	Family	Description	Entry	Clan	Envelope		Alignment		HMM		HMM	E-value
			type		Start	End	Start	End	From	To	length	
	ArgoN	N-terminal domain of argonaute	Domain	n/a	110	246	111	246	2	138	138	5.5e-29
	ArgoL1	Argonaute linker 1 domain	Domain	n/a	256	306	256	305	1	51	52	1,00E-19
	PAZ	PAZ domain	Domain	CL0638	311	437	314	436	4	136	137	2.2e-24
	ArgoL2	Argonaute linker 2 domain	Family	n/a	446	492	448	492	3	47	47	2,00E-14
	ArgoMid	Mid domain of argonaute	Domain	n/a	502	582	503	581	2	83	84	5,00E-12
	Piwi	Piwi domain	Family	CL0219	594	915	594	913	1	300	302	6.2e-113

HvAGO18	Family	Description	Entry	Clan	Envelope		Alignment		HMM		HMM	E-value
			type		Start	End	Start	End	From	To	length	
	ArgoN	N-terminal domain of argonaute	Domain	n/a	233	368	235	368	3	138	138	5.1e-30
	ArgoL1	Argonaute linker 1 domain	Domain	n/a	383	433	383	432	1	51	52	1.1e-20

	PAZ	PAZ domain	Domain	CL0638	438	565	439	564	2	136	137	2.8e-26
	ArgoL2	Argonaute linker 2 domain	Family	n/a	574	620	575	620	2	47	47	7,00E-11
	ArgoMid	Mid domain of argonaute	Domain	n/a	627	706	628	705	2	83	84	3.4e-13
	Piwi	Piwi domain	Family	CL0219	714	1017	715	1015	2	300	302	7.9e-101

HvRDR1a	Family	Description	Entry	Clan	Envelope		Alignment		HMM		HMM	E-value
			type		Start	End	Start	End	From	To	length	
	RdRP	RNA dependent RNA polymerase	Family	n/a	377	936	377	936	1	586	586	2.3e-179

HvRDR1b	Family	Description	Entry	Clan	Envelope		Alignment		HMM		HMM	E-value
			type		Start	End	Start	End	From	To	length	
	RdRP	RNA dependent RNA polymerase	Family	n/a	443	1002	443	1002	1	586	586	2.1e-181

HvRDR2	Family	Description	Entry	Clan	Envelope		Alignment		HMM		HMM	E-value
			type		Start	End	Start	End	From	To	length	
	RdRP	RNA dependent RNA polymerase	Family	n/a	391	972	391	971	1	585	586	2.7e-170

HvRDR3	Family	Description	Entry	Clan	Envelope		Alignment		HMM		HMM	E-value
			type		Start	End	Start	End	From	To	length	
	RdRP	RNA dependent RNA polymerase	Family	n/a	400	1036	403	1035	5	585	586	2.2e-117

HvRDR4	Family	Description	Entry	Clan	Envelope		Alignment		HMM		HMM	E-value
			type		Start	End	Start	End	From	To	length	
	RdRP	RNA dependent	Family	n/a	363	1016	367	1015	6	585	586	1.3e-105

		RNA polymerase										

HvRDR6a	Family	Description	Entry	Clan	Envelope		Alignment		HMM		HMM	E-value
			type		Start	End	Start	End	From	To	length	
	RdRP	RNA dependent RNA polymerase	Family	n/a	440	1026	440	1026	1	586	586	4.1e-182

HvRDR6b	Family	Description	Entry	Clan	Envelope		Alignment		HMM		HMM	E-value
			type		Start	End	Start	End	From	To	length	
	RdRP	RNA dependent RNA polymerase	Family	n/a	439	1015	439	1015	1	586	586	4.3e-181

Table S5. Conserved motifs of *Hv*DCLs identified by MEME

No	Motif length (aa)	Amino acid sequence	Domain
1	66	QRCQETFCYZRLEFLGDAVLKYLVTRHLFLKYPDLHEGQLTDLRSAAVNNENLAQLAVKKNLQGYJ	Rnase 3
2	72	EIVDKFRSGKVNLLVATSVAEEGLDVPSCNLVIRFDLPKTVCSYIQSRGRARMPNSDYILMVERGNVSQZTL	Helicase C
3	95	VFLVPTVVLVTQQAEVIREHTDLRVGQYCGEMGVDFWDAARWRREVESKZVLVMTPQILLDALRHSFLR LDDIALLIFDECHHATGNHPYACIMK	DEAD
4	69	CKAPKVLGDIVESIAGAIFLDSGFDLDVVWKVFKPLLEPLVTPETLELPPVRELQELCQQQGYFLEYKK	not found
5	41	HRWLCSKTIADVVEALIGAYYVEGGEKAAFAFMKWJGIBVE	not found
6	41	ARRYQLEVLERAKRGNTIAFLETGAGKTLIAVLLJREYGHR	not found
7	42	NIYRVESTGATVTLNSSVSLIHFYCSKLPSDRYFIPKPEFIV	not found
8	58	EHSDSGILEAVKDGYLSPKVQALIKILLSYRHSEDVRCIIFVERVITARVLERLLAEJ	not found
9	200	FYHTTPKDKRPAVFGMTASPVNLKGVTSQEDCAIKIRNLESKLDSVVCTIKDRKELEKHVPMPLEVVVQY DKAATLWSLHEQIKQMEAAVEEAALSSSKRTKWQFMGARDAGSRDELRLVYGVSERTESDGAANLIQKLRAI N	not found
10	199	YALGELGQWCAYKVAQSFLTALQNDERANYQVDVKFQESYLKKVVDLLHCQLTEGAA DHLEVSNGIKYFHEMQVPIGVVYLLLPLVSGKIDWCSIKFSSSPIYEANNKDMRHCHSCKDIDLLQTKDGPF CRCILKNSIVCTPHNNIFYVISGFLDLDANSLLPQHDGSVLTYKDYFKTRHGLTLTFENQPLLAGRKHVEVR NFLHKCYYKKEKEPGDRYSVELPPELCRIIMSPVSANTLHIFSYIPSIMYRIQCM	PAZ
11	164	EVLSTTMDLFVARTMITKASLVFRGPIEITESQLVLLKSFHVRLMSIVLDVDVDPSTTPWDPAKAYLFVPVG AEKCMDPLREIDWTLVNNIVNTDAWNNPLQRARPDVYLGTNERTLGGDRREYGFGKLRHGTAFGQKAHP TYGIRGAIAEFDIVKASGLVPAR	not found
12	29	SISLAKQAVCLKACKKLHELGALTDHLLP	Dicer dimer
13	41	YATYADYYKQKYGIELSHPEQPLLKAKQSHNLHNLLSSRFE	PAZ
14	106	REELHEMLIPLVLKPSWIKESSSVNLHFYYIDFJPDPEDRRYAGFGLFMENPLPDEAEKLEVDLHLAPNRIVK AGITPLGKIZFBKEQLMLGKLFQEMFLKVJFDR	not found
15	45	LIVIDLSKDIJRSFYLLPSIMHRLESLLLASQLKDEISYSVPEAS	not found
16	28	IBVEELEALLGYKFKDKGLLLEALTHPS	not found
17	29	EIAGAANPNKKSAKKLAAQLLLEDLKERG	not found
18	29	KGGPRSALFELCKRLQWPMPZFECVEEEG	not found
19	29	FYSGSDKRPRIFGMTASPVIRKGASSKLD	not found
20	118	CEAQIAQLELLLDAKIYIVEDRNEJESFSPPAKIVNKYYDPYLVDFEDLKSKLQILYEEYDALJVELQESPPNK FKDTDBILETSRKSLSRYHGKILYCLBDLGPIITSEVVKIYIES	not found

Table S6. Conservative motifs of *Hv*AGOs identified by MEME

No	Motif length (aa)	Amino acid sequence	Domain

1	80	NILPGTVVDSKICHPTEFDFYLCSHAGIQGTSRPAHYHVLWDENNFTADGLQTLTNNLCYTYARCTRSVSIVPP AYYAHL	Piwi
2	29	QRIIFYRDGVSEGQFYQVLLYELDAIRKA	Piwi
3	46	THPHPGEDSSPSIAAVVASQDWPEITKYAGLVCAQAHRQELIQDLF	Piwi
4	41	SWRGFYQSIRPTQMGLSLNIDMSSTAFIEPLPVIDFVAQLL	Argonaute linker 1
5	41	QVGNQQRPNYLPMEVCKIVEGQRYSKRLNEKQITALLKVTC	PAZ
6	80	AYYEDPYAQEFGIKIDEQLASVEARVLPPPRLKYHDSGREKDVLPRVGQWNMMNKKMVNGGRVSHWACINF SRNVQDNAA	Argonaute linker 2
7	23	PPVTFVVVQKRHHTRLFANNHND	not found
8	23	NNGSLYGDLKRICETELGIVSQC	not found
9	29	KMSKQYLANVALKINVKVGGRNTVLVDAL	not found
10	25	AYDGRKSLYTAGPLPFDSKEFVVNL	not found
11	42	KIKKALRGVKVEVTHRGNMRRKYRISGLTSQATRELSFPVDD	PAZ
12	29	ARSMRGAGSVAVRPLPALKENVKRVMFYC	not found
13	29	RCIVKANHFFAELPDKDLHHYDVTITPEV	not found
14	21	QVLDIVLREFPTARYCPVGRS	not found
15	41	IMCQISGMDFALEPVLPPLTARPEHVERALKARYQDAMNML	not found
16	15	PLVSDIPTIIFGADV	not found
17	63	MSVLNDALRHSNYDSDPMLRASGISIAQNFTQVEGRVLQPPKLKAGNGEDIFPRNGRWNFNNK	Argonaute linker 2
18	34	RQFRVVIKYAARADLHHLAMFLAGRQPDAPQEAL	not found
19	8	GGGGGRGG	not found
20	21	VTGGMIRELLISFRKATGQKP	not found

Table S7. Conserved motifs of *Hv*RDRs identified by MEME

No	Motif length (aa)	Amino acid sequence	Domain
1	114	PMDYTPARPKTLDHDVTIEEIQEYFVBYMVNDSLGIISNAHVVHADRSPLKAESPECIZLAELFSIAVDFPKTGVP AEMPPALRPKEYPDFMEKLDKPTYISEGVJGKLYREIK	RdRP
2	78	IKLSLRPSMKKFESENTMLEVLAWSKYQPCFLNRQJITLLSTLGVRDEIFEQMQZEMVSELBRMLTDPEAALEVJ TKM	RdRP
3	59	KTALYNRVLSVLSEGITIGGKKFEFLAFSASQLRDNSAWMFASNGGLTAEDIREWMGNF	RdRP
4	79	GEETNTAAEMLLAGYEPDTEPYLSMMLQAFRASQLLELRTKSRIFVPKGRWLMGCLDETGILEYGQVFIRASKPS KENK	RdRP
5	29	PHPBECSGSDLDGDIYFVSWDPKLJPPRK	RdRP
6	57	RVQITPSKVYCLGPEVEVSNRVVRHYSAYADNFLRVTFVDEDMEKLNSNALSPRSAP	RdRP
7	29	ITGKVVVAKNPCLHPGDIRVLEAVYVPAL	RdRP
8	29	DTNGTKYIFSDGIGKISEDLAKEVAKKIG	not found
9	29	IRNVAKYAARMGQSFSSSYETLKVHPHEV	not found
10	21	FPPSAFQIRYGGYKGVVAVDP	RdRP
11	47	WDMDEPYILSGKTIGZARMLFMHVHTAPTLAKYMARFALILSKTITL	RdRP
12	34	YDFKLGNLMDYYGIKSEAEILTGCILKMAKYFTK	not found
13	80	MIKVNADKSLSGVQSFNSLEIVTTSNPPKRTLTSKNLIALLSYGGVPEEFFLEJLQNAJEEAENAFYDYRAALNIAFN YG	RdRP
14	51	PLDEPYLQHRLSFLLKQERKGJKQGKLPIDECYYLMGTVDPTGELKPNEVC	RdRP
15	113	IEDVLSVDYIRSLKYLSMAQFESEIWRTFGHKYIAASDRAKNLDSDPGMTKVYHCNVEIRGDSVVKIFKGPYIENTRT HLQKVVGDDNVLVVKFMGISSDTETDFSTYLEHYH	not found
16	25	EDDDDPWIRTTDFTPSGSIGRCSIL	not found
17	21	EEAYEAKASAWYHVTYHPEYW	not found
18	56	EKKEIKKKMLKLVDJYYDALDAPKTGNKVNJPRDLKVKQYPHYMERDPSYHSTSIL	RdRP
19	37	VPHAEGVPYEVLFLVNSLVHNGVLSGPHLTPEFFGLL	not found
20	15	HHLVDCVVFPQKGER	not found

Table S8. Function of *Arabidopsis* RNAi components with barley homologues

**Barley RNAi factor**	***Arabidopsis* homologue**	**Function of *Arabidopsis* homologue according to UniProt**
*Hv*AGO1a, *Hv*AGO1d	*At*AGO1	component of miRNA pathway, mRNA cleavage, PTGS
*Hv*AGO2	*At*AGO2	mRNA cleavage, PTGS
*Hv*AGO4	*At*AGO4	TGS, DNA-methylation, histone modification, siRNA pathway
*Hv*AGO5a, *Hv*AGO5b	*At*AGO5	PTGS, siRNA, miRNA binding, probable role in antiviral silencing
*Hv*AGO6 *Hv*AGO6b	*At*AGO6	PTGS, TGS, siRNA pathway, RNA-directed DNA-methylation. May be redundant with AGO4
*Hv*AGO7	*At*AGO7	PTGS, miRNA, siRNA binding
*Hv*AGO10	*At*AGO10	PTGS, essential for developmental processes, SAM-organisation
*Hv*DCL1	*At*DCL1	PTGS, miRNA biogenesis, flowering
*Hv*DCL2	*At*DCL2	PTGS, siRNA processing, transgene and antiviral silencing
*Hv*DCL3, *Hv*DCL5	*At*DCL3	PTGS, siRNA pathway, antiviral silencing. May be redundant with DCL1
*Hv*DCL4	*At*DCL4	PTGS, ta-siRNA biogenesis, leaf development timing, antiviral silencing
*Hv*RDR1a, *Hv*RDR1b	*At*RDR1	antiviral silencing, involved in 21-26 nt sRNA biogenesis
*Hv*RDR2	*At*RDR2	TGS, siRNA production
*Hv*RDR3	*At*RDR3a	probable siRNA generation
*Hv*RDR4	*At*RDR3b	probable siRNA generation
*Hv*RDR5	*At*RDR3c	probable siRNA generation
*Hv*RDR6a, *Hv*RDR6b	*At*RDR6	PTGS, transgene silencing, leaf developmental timing
